# Origin and evolution of HIV-1 subtype A6

**DOI:** 10.1371/journal.pone.0260604

**Published:** 2021-12-13

**Authors:** Syed Hani Abidi, Lazzat Aibekova, Salima Davlidova, Aidana Amangeldiyeva, Brian Foley, Syed Ali

**Affiliations:** 1 Department of Biological and Biomedical Sciences, Aga Khan University, Karachi, Pakistan; 2 Department of Biology, School of Sciences and Humanities, Nazarbayev University, Nur-Sultan, Kazakhstan; 3 Department of Biomedical Sciences, Nazarbayev University School of Medicine, Nazarbayev University, Nur-Sultan, Kazakhstan; 4 Theoretical Biology and Biophysics Group, Los Alamos National Laboratory, Los Alamos, New Mexico, United States of America; Centers for Disease Control and Prevention, UNITED STATES

## Abstract

**Background:**

HIV outbreaks in the Former Soviet Union (FSU) countries were characterized by repeated transmission of the HIV variant AFSU, which is now classified as a distinct subtype A sub-subtype called A6. The current study used phylogenetic/phylodynamic and signature mutation analyses to determine likely evolutionary relationship between subtype A6 and other subtype A sub-subtypes.

**Methods:**

For this study, an initial Maximum Likelihood phylogenetic analysis was performed using a total of 553 full-length, publicly available, reverse transcriptase sequences, from A1, A2, A3, A4, A5, and A6 sub-subtypes of subtype A. For phylogenetic clustering and signature mutation analysis, a total of 5961 and 3959 pol and env sequences, respectively, were used.

**Results:**

Phylogenetic and signature mutation analysis showed that HIV-1 sub-subtype A6 likely originated from sub-subtype A1 of African origin. A6 and A1 pol and env genes shared several signature mutations that indicate genetic similarity between the two subtypes. For A6, tMRCA dated to 1975, 15 years later than that of A1.

**Conclusion:**

The current study provides insights into the evolution and diversification of A6 in the backdrop of FSU countries and indicates that A6 in FSU countries evolved from A1 of African origin and is getting bridged outside the FSU region.

## Introduction

Since 2010, cases of new HIV infections have declined by 23% worldwide, whereas these numbers in Eastern Europe and Central Asia have escalated by 72% between 2010 and 2019, reaching 1.7 million HIV positive people by 2019 [1]. Among the Former Soviet Union (FSU) countries, Russia has the highest prevalence of HIV positive people [1]. Collapse of Soviet Union in the early 90s led to massive transmigration within the FSU countries, which in turn led to spread of infectious diseases. The epidemic in the FSU countries was characterized by repeated transmissions of a variant of HIV, subtype A6 (previously known as A_FSU_), that initially emerged among people who inject drugs (PWID) and heterosexual populations [2, 3]. A6/A_FSU_ was originally recognized as a sister clade of A1, until it was recently re-classified as a new HIV-1 sub-subtype, A6 [4, 5]. Previous phylogeographic analyses have shown the origin of this variant to be the Demographic Republic of Congo [3]. Currently, A6 is responsible for the fastest growing epidemic in the FSU countries. While travel and trade thrives within FSU countries, and flexible immigration laws facilitate cross-border migrant mobility, risk of HIV transmission continues to grow within this region [6, 7]. Furthermore, considering the A6 epidemic has now been expanding within FSU for almost three decades, chances are that this variant has now been transmitted into countries outside the FSU region.

The present study used phylogenetic/phylodynamic and signature mutation analyses to explore the origin, transmission, and evolution of subtype A6.

## Methodology

### Collection of sequences, alignment and editing

For this analysis, two different datasets were collected. For initial phylogenetic analysis, a total of 841 full-length reverse transcriptase sequences, from A1, A2, A3, A4, A6 and newly described A8 [8] sub-subtypes of subtype A, spanning the corresponding nucleotides 2550→3869 in HXB2 genome, were retrieved from the Los Alamos HIV database (https://www.hiv.lanl.gov/content/index). After removing the duplicate sequences, 557 sequences were used for initial phylogenetic analysis (S1 Table).

For phylogenetic clustering and signature mutation analysis, a total of 5961 and 3959 *pol* sequences of, respectively, HIV-1 subtype A1 and subtype A6 (S2 Table), spanning 2253→3254 corresponding nucleotides in HXB2 genome were retrieved from HIV Los Alamos database (http://www.hiv.lanl.gov). From the same database, 1075 and 703 *env* sequences of, respectively, HIV-1 subtype A1 and subtype A6 (S2 Table), spanning 6213→8795 corresponding nucleotides in HXB2 genome (complete *env* CDS) were also retrieved. The said genomic regions were selected, since they were represented most in the HIV Los Alamos database. The sequences were aligned and edited using *MEGA 7*.*0* software. Editing involved removal of non-nucleotide characters and trimming of the ends to bring all the sequences to same length. The final length after editing was 1002 bp for *pol* and 2575 bp for *env*.

### Analysis of phylogenetic relationship among different HIV-1 A sub-subtypes

To analyze phylogenetic relationship among different A sub-subtypes, a Maximum Likelihood (ML) phylogenetic tree was constructed using PhyML 3.0 software [9] using a total of 557 *pol* sequences. ML tree was constructed using the following parameters: substitution model: general time reversible (GTR) with empirical equilibrium frequencies, transition / transversion ratio = 4 (default), estimated proportion of invariable sites, estimated gamma shape parameters and number of substitution rate categories = 8 (default); starting trees = BIONJ; Tree optimization = Tree topology and branch length; Tree improvement = Nearest Neighbor Interchange (NNI); Branch Support = approximate likelihood-ratio test, Shimodaira–Hasegawa-like (aLRT SH-like). The resulting tree was refined and visualized using *Figtree 1*.*4*.*2* software (http://tree.bio.ed.ac.uk/software/figtree/). Significant clusters were identified by SH value of ≥ 0.85. The final tree was color-coded using the Rainbow Tree software [10]. Phylogenetic relationship among different A sub-subtypes was also confirmed using a Neighbor-joining phylogenetic tree constructed using *TreeMaker* software (https://www.hiv.lanl.gov/components/sequence/HIV/treemaker/treemaker.html) using 1850 *gag* sequences (A1 = 1606, A2 = 56, A3 = 14, A4 = 3, A6 = 171). The genetic distance between A1 and A6 sequences were determined using Bioedit tool, Neighbor-joining distance tree algorithm [11].

### Analysis of phylogenetic relationships between A1 and A6 sequences

HIV-1 subtype A1 and A6 *pol* (5961 and 3959, respectively) and *env* (1075 and 703, respectively) sequences were used to construct two separate phylogenetic trees using the *TreeMaker* software, using Neighbor-Joining algorithm. The final tree was visualized using the *Figtree 1*.*4*.*2* software (http://tree.bio.ed.ac.uk/software/figtree/).

### Effective population size and the time to the most recent common ancestor (tMRCA) for A1 and A6 sequences

Bayesian Markov Chain Monte Carlo (MCMC) inference was applied to estimate the effective population size and tMRCA of HIV subtypes A1 and A6, using BEAST v1.10.4 software [12]. For this analysis, a total of 231 A1 and A6 *pol* sequences were used that were sampled during 1985 to 2017. Sequences were selected using the approach described previously [13, 14], where a limit was set to 5–6 sequences per country and per year. If 5 or less sequences were represented in a country all of them were included in the analysis. Prior to Bayesian analysis, best model for the representative dataset was determined using model selection employed in MEGA 7 software. Using this analysis, Hasegawa–Kishono–Yano (HKY) nucleotide + Gamma + invariant sites (HKY+G+I) was found to best describe the substitution patterns based on its low Bayesian Information Criterion (BIC) scores. Hence, Bayesian analysis was performed using the following parameters: uncorrelated lognormal relaxed molecular clock; Hasegawa–Kishono–Yano (HKY) nucleotide substitution model; estimated base frequencies, and gamma distribution model + invariant sites for heterogeneity among nucleotide sites. The analysis was performed using Bayesian skygrid plot as tree prior [15]. The MCMC chain length was set at 2×10^8^, which gave an effective sample size (ESS) of >200. MCMC sample analysis and Bayesian skygrid plot construction was performed using Tracer v1.7.1.

### Analysis of A1 and A6 amino acid signatures

For signature mutation analysis, a total of 5961 *pol* and 1075 *env* sequences for HIV-1 subtype A1; and 3959 *pol* and 703 *env* sequences for HIV-1 subtype A6 were used.

The sequences were translated into protein sequences using the *MEGA 7*.*0* software, and the translated sequences were used to perform signature analysis using the *VESPA* (Viral Epidemiology Signature pattern analysis) tool (https://www.hiv.lanl.gov/content/sequence/VESPA/vespa.html) [16]. The software calculates the frequency of each amino acid at each position in an alignment for the query and background set and selects the positions (signature positions) for which the most common character in the query set differs from that in the background set. For this analysis, A6 and A1 sequences were used, respectively, as query and background. Additionally, both *pol* and *env* A1 and A6 sequences sharing nodes were also analyzed separately for signature mutations.

## Results

### Analysis of phylogenetic relationships between different HIV-1 A sub-subtypes

**Fig 1:**
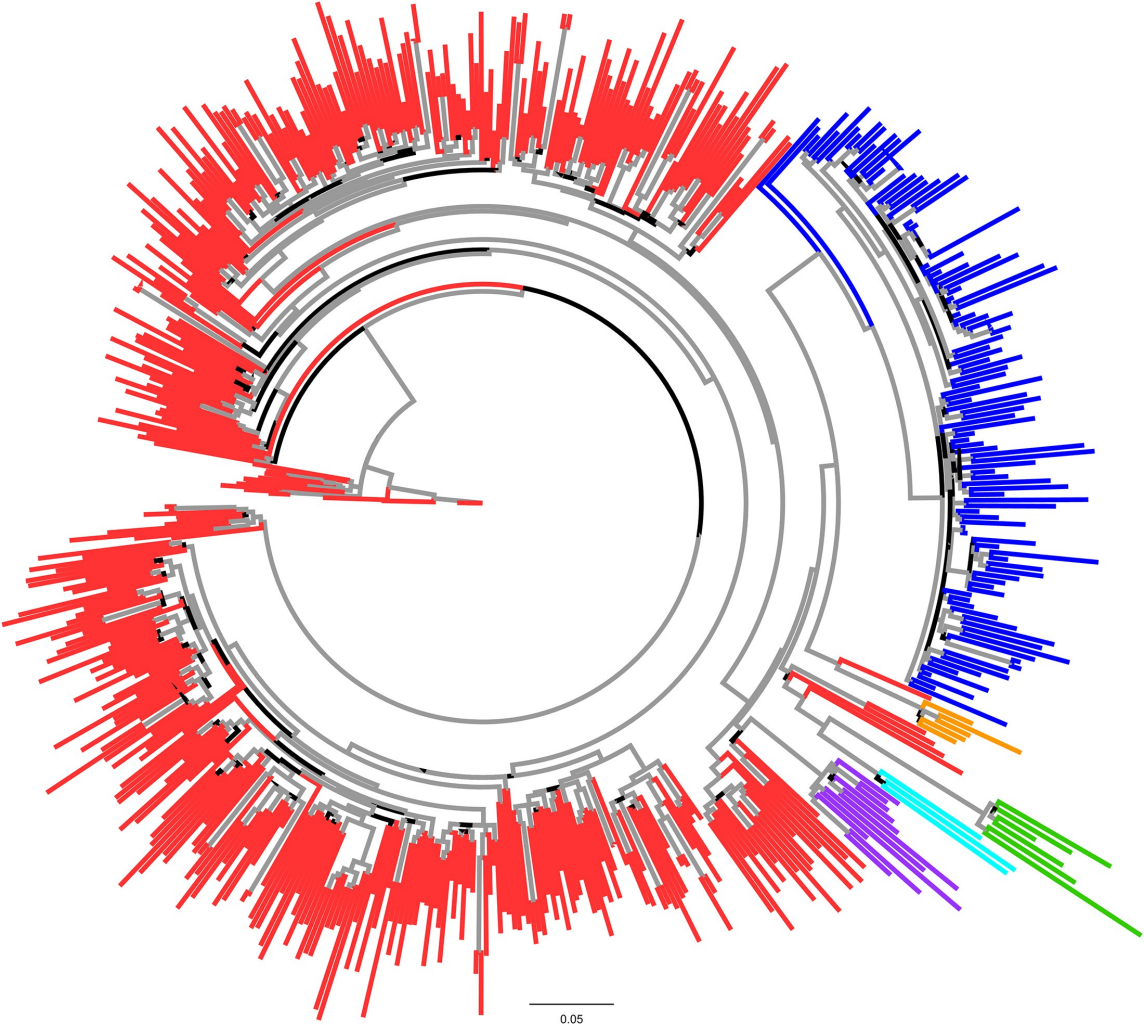
Maximum Likelihood phylogenetic tree of full-length RT HIV sub-subtype A sequences: The tree was constructed using full-length RT sequences. HIV-1A sub-subtypes are shown in red (A1), green (A2), purple (A3), turquoise (A4), blue (A6) and orange (A8) colors. Grey colored branches signify ≥ 0.9 support value.

An ML phylogenetic tree was constructed to study the relationship among HIV-1 A sub-subtypes. A6 sequences formed a discrete cluster with a strong (>0.90) node support value (Fig 1, blue colored sequences). Ancestral node of A6 cluster was shared by an A1 sequence from Switzerland (accession number JQ403028.1) with a strong node support value (>0.90) indicating the likely evolution of A6 sub-subtype from A1. Rest of the A sub-subtypes, especially sub-subtype A3 and A8 were found to cluster with A1 sequences at different discrete nodes (Fig 1).

The observation was further corroborated by ML phylogenetic tree based on *gag* sequences, where A6 sequences formed a discrete cluster with a strong (>0.90) node support value. The ancestral node of A6 cluster was shared by an A1 sequence of African (Kenyan; accession numbers GQ430197 and GQ430049) origin (S1 Fig, blue colored sequences) with a strong node support value (>0.90). Furthermore, several Kenyan A1 *gag* sequences exhibited mixing with A6 sequences (S1 Fig, blue colored sequences) indicating close phylogenetic relationship between A6 sub-subtype from A1. Here also, rest of the A sub-subtypes, especially sub-subtype A3 were found to cluster with A1 sequences (S1 Fig), however, the node did not exhibit significant statistical support value.

A1 and A6 clustering was confirmed by a distance tree (S2 Fig, black arrow), where ancestral A1 sequence from Switzerland (accession number JQ403028.1) shared ancestral node of A6 cluster, where genetic distance between A6 and A1 was found to be 0.0715 (S2 Fig).

### Effective population size and time of the most recent common ancestor (tMRCA) for A1 and A6 sequences

**Fig 2:**
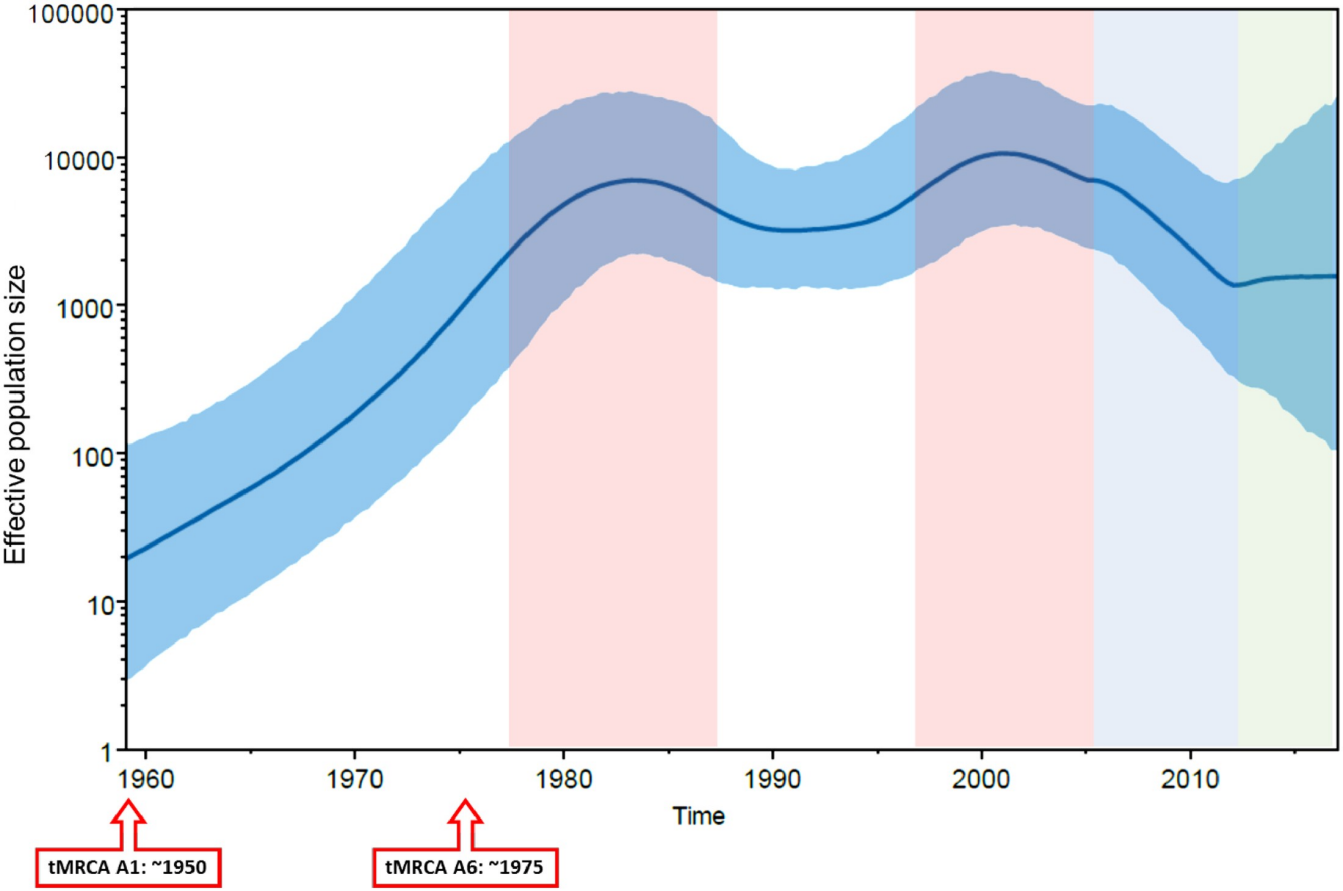
Effective population size and time to most recent common ancestor for HIV-1 subtype A1 and A6. Bayesian Skyline plot, based on a ‘relaxed clock’ coalescent framework analysis, was constructed using 231 sequences (representing all years and countries). X-axis represents time in years, while Y-axis shows the effective population size. The thick blue line and solid grid (blue) represents mean 95% lower posterior density (HPD) intervals. The tMRCA of HIV-1 subtype A1 and A6 are indicated with red boxed arrows. Pink, light blue and green shaded areas represent the period of increase in viral effective population size, decline and plateau phase, respectively.

Since the previous analysis showed A6 sub-subtype to possibly have evolved from A1 sequences (Fig 1), we used Bayesian Skygrid analysis to determine tMRCA of A1 and A6 subtypes. Using this analysis, tMRCA of HIV-1 subtype A1 and A6 was estimated to be around 1950 and 1975, respectively (Fig 2, red boxed arrows). Compared to the origin of tMRCA, the Bayesian Skygrid plot identified a growth in viral effective population size (correlating with effective number of infections and/or transmission opportunities [17]) especially from 1975 to 1985 (Fig 2, pink shaded area). Effective population size declined from 2005 onwards (Fig 2, light blue shaded area) and became stationary from 2010 onwards (Fig 2, green shaded area). The mean substitution rate from this plot was determined to be 2.3 x 10^−3^.

### Analysis of the A1 and A6 phylogenetic clustering

**Fig 3:**
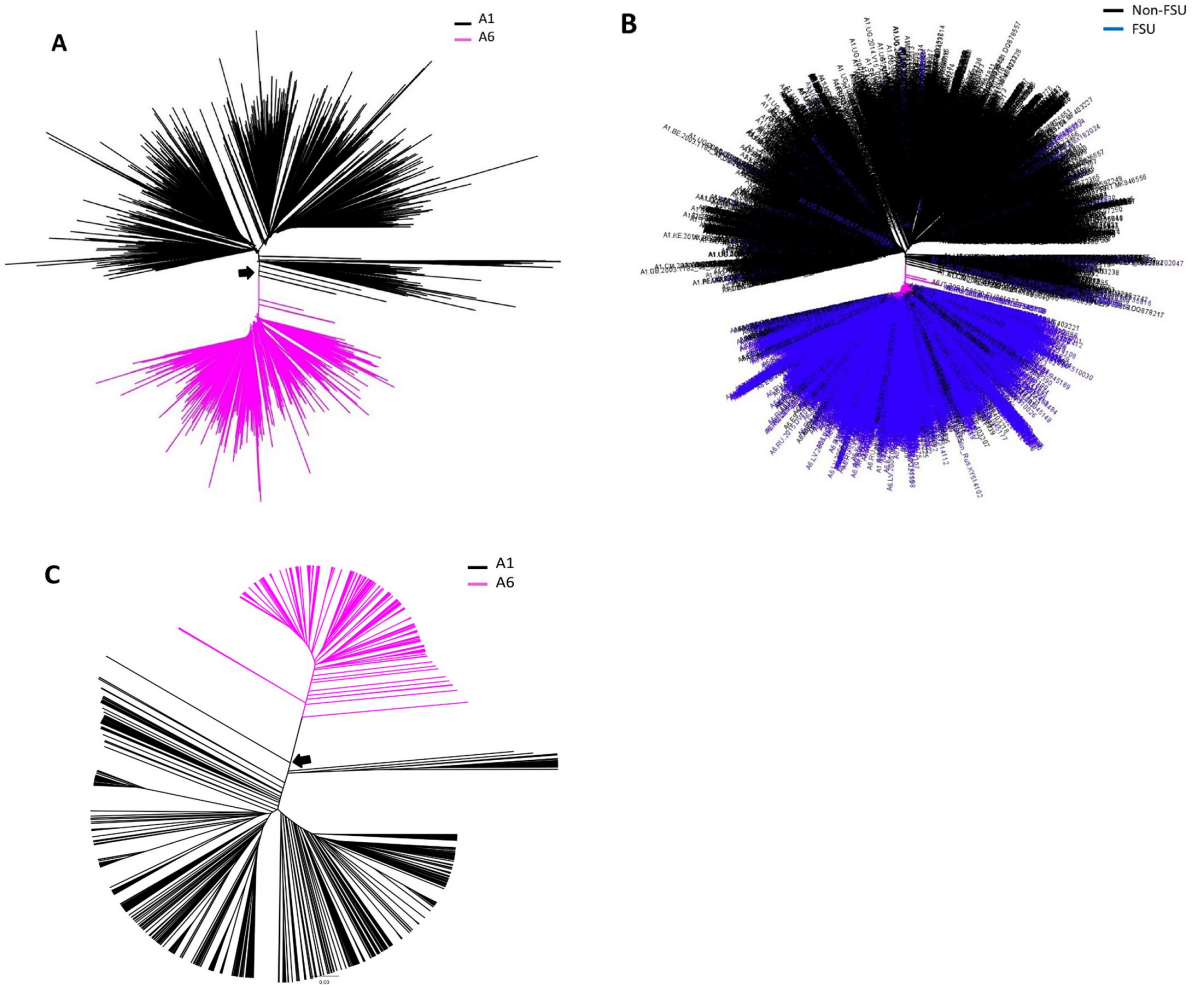
Phylogenetic analysis of HIV subtype A1 and A6 *pol* and *env* sequences: Using NJ method, HIV A and B) *pol* and C) *env* sequences, representing sub-subtypes A1 and A6, were analyzed. **A)** A1 and A6 sequences are shown in black and magenta, respectively. **B)** *pol* sequences from FSU and non-FSU countries are shown in blue and black colors, respectively. **A** and **C)** Black arrow points to sequences at the node shared (defined as nodes where A1 and A6 sequence(es) converge) between A1 and A6 clusters. Significant nodes had *aLRT* support value of ≥ 0.85.

Phylogenetic relationships among the HIV subtype A1 and A6 pol sequences was inferred using a NJ tree. Analysis of the tree revealed that HIV-1 subtype A1 and A6 *pol* sequences from different countries separated into distinct clusters (Fig 3A, magenta and black branches). The A1 cluster primarily comprised of sequences from non-FSU countries, with few FSU sequences (from Belarus, Ukraine, and Russian Federation). In contrast, A6 cluster primarily comprised of sequences from FSU counties, mainly from Russia and Ukraine, while few non-FSU sequences (from Bulgaria, Cyprus, Czech Republic, Mongolia, Poland, Sweden, Slovenia, Turkey, and United States) were also present (Fig 3B). Interestingly, A1 nodes conjoining with A6 cluster were occupied by sequences from Cameroon (accession numbers AY444189 and AY444189) and the Democratic Republic of the Congo (accession number FR666640), both of African origin (Fig 3A, black arrow)—likely indicating African transmission networks. This observation was confirmed by earlier tree (Fig 1), where ancestral nodes of A1 was occupied by sequences from Cameroon (accession numbers AM279375, AM279372, AM279371 and GU201516) and the Democratic Republic of the Congo (accession numbers MH705163, AF286240, AF286238, AM000053).

Similar analysis of A1 and A6 *env* sequences also revealed two completely distinct A1 and A6 clusters, where A1 nodes conjoining with A6 cluster were occupied by sequences also of African origin, namely from the Democratic Republic of the Congo (Fig 3C, black arrow; accession numbers AJ401040 and HM623589) supporting the observation of African transmission networks in A6 prevalent countries (Fig 3A and 3C).

### Analysis of A1 and A6 amino acid signatures

**Fig 4:**
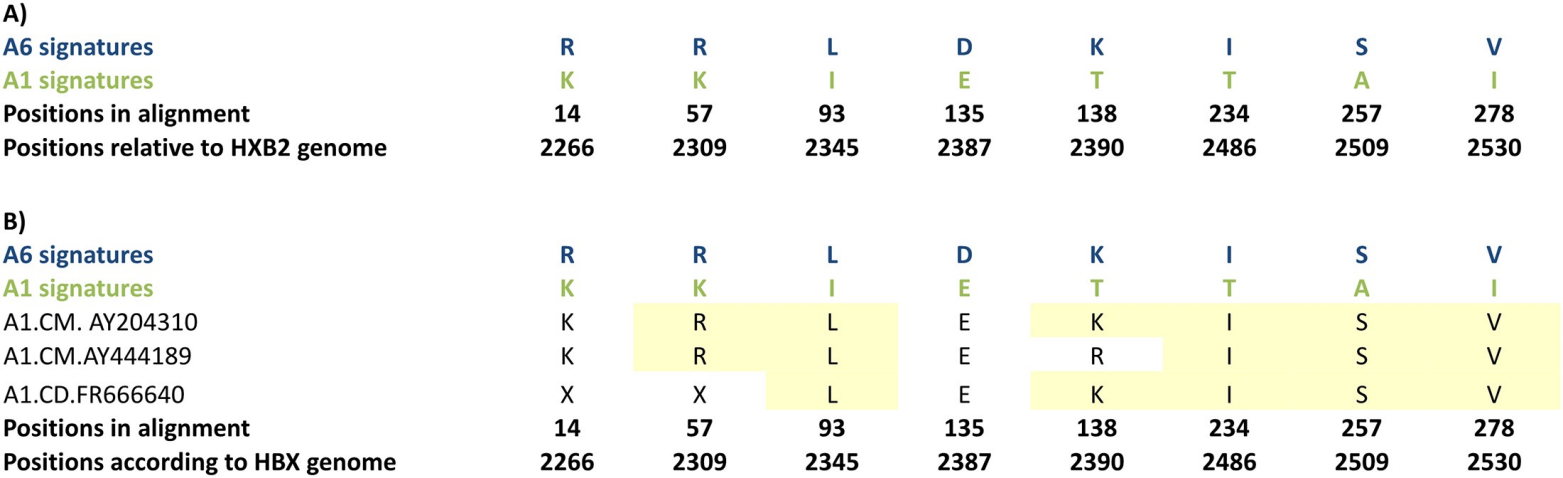
Distinguishing Pol signature mutations in subtypes A1 and A6. The A1 and A6 sequences were compared using VESPA. A1 and A6 sequences were used as background and query, respectively. The atypical (signature) amino acids are shown in **A)** A1 and A6 sequences, and **B)** A1 sequences from two countries (Cameroon and the Democratic Republic of the Congo) sharing ancestral node with the A6 cluster (Fig 3A). Yellow highlights show A6 signature amino acids in A1 sequences.

**Fig 5:**
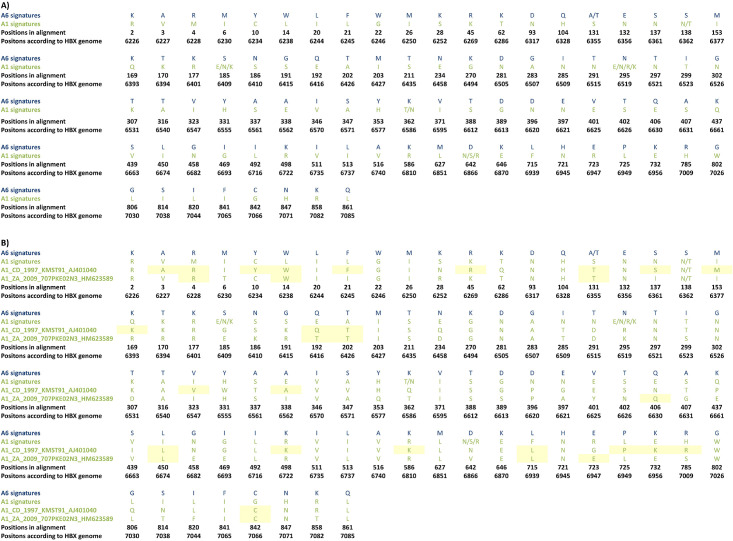
Distinguishing Env signature mutations between subtypes A1 and A6. A1 and A6 sequences were compared using VESPA. A1 and A6 sequences were used as background and query, respectively. The atypical (signature) amino acids are shown in **A)** A1 and A6 sequences, and **B)** A1 sequences from two countries sharing ancestral node with the A6 cluster (Fig 3B). Yellow highlights show A6 signature amino acids in A1 sequences.

We employed VESPA software to analyze Pol and Env amino acid sequences to identify amino acid signatures that distinguish A6 from A1 subtype (Figs 4 and 5 and S3 Table). For effective representation, only the dominant amino acids residues at each position are displayed (Figs 4 and 5). Analysis of the Pol sequences revealed 8 sites carrying mutations distinguishing between the subtypes A6 and A1 sequences (Fig 4A). As two A1 sequences from Cameron, and 1 A1 sequence from the Democratic Republic of the Congo occupied the A6 ancestral nodes (Fig 3A, black arrows) A6 signatures were specifically compared with these three sequences. Signature analysis of these A1 Pol sequences revealed presence of A6 signatures at 6/8 sites in sequences from Cameroon, and 5/8 sites in sequence from the Democratic Republic of the Congo (Fig 4B, yellow highlights).

Signature mutation analysis of full-length A6 an A1 Env sequences revealed 88 sites to be unique between the two sub-subtypes (Fig 5A). As two A1 sequences from the Democratic Republic of the Congo occupied the A6 ancestral nodes (Fig 3C, black arrows), A6 signatures were compared with these two sequences. Signature analysis of A1 Env sequences from these two countries revealed A6 signatures at 24 (out of 88) different sites (Fig 5B, yellow highlights).

## Discussion

Here we performed phylogenetic/phylodynamic and signature mutation analysis to show that FSU-indigenous HIV-1 sub-subtype A6 likely originated from sub-subtype A1. A6 carried signature mutations that distinguished it from A1, while tMRCA for A6 dated to 1975, 15 years later than that of A1.

Phylogenetic analysis of HIV-1 sub-subtype A RT and *gag* sequences revealed that A6 formed a discrete cluster whose ancestral node was shared by A1 sequences, indicating that although A6 has diverged into a distinct sub-subtype, phylogenetically it is related most closely to (and most likely originated from) sub-subtype A1. These observations corroborate the findings in previously published reports on subtype A6, where it has been suggested that A6 was an indigenously, and predominantly, circulating strain in Former Soviet Union (FSU) countries, mainly representing the initial HIV epidemics in this region [4, 6, 18]. For the same reason, A6, originally perceived as a variant of subtype A, was referred to as A_FSU_ because of its homogeneous distribution in the FSU population [4, 6].

Phylogenetic analysis of A1 and A6 sequences only revealed that few A1 sequences, of African origin, occupied the A6 ancestral nodes—likely indicating involvement of African transmission networks in the evolution of A6. These observations are supported by previous reports on A1 and A6 global transmission and phylogenetics, where most of the subtype A1 transmissions were attributed to African strains [3, 13]. In the HIV Los Alamos Database, the high-risk behavior associated with HIV transmission is reported mainly for FSU sequences from Russia and Ukraine. The main high-risk groups are injection drug users, men who have sex with men and heterosexual contact. However, no information exists about sequences from non-FSU countries, except Mongolia, where high-risk behavior was heterosexual contact. In this scenario, the HIV transmission routes can only be speculated.

Back in the 1950s Soviet Union established political relationship with African countries, such as Angola, Algeria, Ghana, Guinea, Mali, Sudan, Morocco and Libya, with aims to implement socialism [19, 20]. Subsequent to these political alliances, back and forth migrant mobility might have led to introduction of the founder strains of A6. Additionally, A6 cluster had sequences from several non-FSU countries, such as Bulgaria, Cyprus, Czech Republic, Mongolia, Poland, Slovenia, and Turkey. A6 sequences from most of these non-FSU countries, except Czech Republic (where A6 sequences were from 1998–2000), were found to be relatively recent, deposited between 2002–2017 (https://www.hiv.lanl.gov/content/index). Analysis of the papers where these samples were originally reported gave insights into A6 transmissions between FSU and non-FSU countries. For example, Bulgaria, Cyprus, Mongolia, Czech Republic and Turkey all reported A6 transmission involving heterosexual and homosexual contact with individuals from Russia and Ukraine [21–25]. Another study reported transmission in Bulgaria and Eastern Europe through PWID networks in Russia and Ukraine [26]. These transmissions might be responsible for the bridging of A6 into Eastern and Central European countries.

Next, we constructed a Bayesian Skygrid plot to deduce tMRCA and transmission dynamics of A1 and A6. The tMRCA of HIV-1 subtype A1 and A6 was estimated to be around 1950 and 1975, respectively, aligning with the hypothesis that A6 originated from A1. The tMRCA for A1 is in agreement with the one previously estimated [13]. *Tongo et al*, suggested that A1 epidemic, initiating within the Congo Basin in late 1950s subtype, expanded to other countries and also diversified to give rise to recombinant forms. The high frequency of subtype A1 in Congo Basin might have contributed to the spread of A1 and A1-derived sequences during the period of 1960–70 [27]. Whereas tMRCA for A6 differed from the tMRCA reported for A_FSU_ (1984) but matched the tMRCA for African ancestor of A_FSU_ (1970) reported by Diez-Fuertes *et al*. [28]. The difference might be due to calculation of tMRCA for A1 and A6 using a single dataset (with A1 and A6 defined as separate taxa) which might have considered A1 ancestral traits. Nonetheless, the tMRCA calculated in this study aligns with the time when initial cases of HIV in the FSU region were reported [12, 18, 20, 29, 30]. Bayesian Skygrid plot identified a growth in viral effective population size (correlating with effective number of infections and/or transmission opportunities [17]) from 1975 to 1985, and then from late 90s to 2005 –corresponding to the collapse of the Soviet Union followed by increased trans-border migrant mobility within FSU region. The effective population size declined from 2005 onwards and became stationary after 2010. This trend explains the previously reported HIV outbreaks in USSR/FSU countries, where initial HIV cases were reported in late 1980s, followed by a massive increase in HIV cases during mid-1990s stabilizing from 2011 onwards [18].

Signature mutation analysis of Pol and Env sequences revealed, respectively, 8 and 88 sites that carried mutations distinguishing between sub-subtypes A6 and A1 sequences. Aptly, A1 *pol* and *env* sequences conjoining with A6 ancestral nodes (Fig 3A and 3C) exhibited A6 signatures at several sites (Figs 4 and 5). These observations further strengthened the hypothesis that A6 diversified into a sub-subtype distinct from A1. To the best of our knowledge, signatures between HIV subtype A sub-subtypes have not been previously explored. In an amino acid or nucleic acid alignment, signature sites are the variables distinctly represented in query sequences when compared to a background set [31]. Signature mutations can be often unique to one or a small number of viral genetic subtypes/variants, as in the case of a diversified variant, or they may be dominant characters present in the majority of sequences, as in the case of epidemic strains [31]. It is being increasingly recognized that viral subtype-specific genetic variation or signature polymorphisms/mutations can have significant impact on evolution and diversification [32, 33]. For example, signature amino acid residues identified in HIV-1 Tat protein are evolve in a subtype-specific manner (unique for Indian HIV-1C) and can be used to design tailored vaccines against the Indian HIV-1C [34].

Taken together, the current study provides insights into origin, evolution and diversification of A6 in the backdrop of FSU countries. Our observations indicate that A6 in FSU countries likely originated from A1 strains of African origin, and is now bridging into non-FSU countries.

## Supporting information

S1 Fig Maximum Likelihood phylogenetic tree of full-length *gag* HIV sub-subtype A sequences: HIV-1A sub-subtypes are shown in red (A1), green (A2), purple (A3), turquoise (A4), blue (A6) colors. Grey-colored branches signify ≥0.9 support value. For clarity, unrelated subtype A1 clusters have been collapsed. The black arrow indicates the A6 ancestral node shared by A1 sequences. SIV *gag* sequence from chimpanzee was used as an outlier to root the tree.
(TIFF)

S2 Fig Distance tree of full-length RT HIV sub-subtype A sequences: The tree was constructed using full-length RT sequences. HIV-1A sub-subtypes are shown in red (A1), green (A2), purple (A3), turquoise (A4), blue (A6) colors. The black arrow indicates the A6 ancestral node shared by A1 sequences. Values on the nodes show genetic distances.
(TIFF)

S1 Table HIV-1 A1, A2, A3, A4 and A6 RT sequences used in the study. (DOCX)

S2 Table Accession numbers of HIV-1 A1 and A6 sequences used in the study. HIV-1 A1 pol sequences were from countries located in South America, the Middle East, Asia, Caribbean, Oceania, Former USSR, Europe, North America, Africa, Sub-Saharan Africa, while HIV A6 pol sequences were from countries located in the Middle East, Asia, Oceania, Former USSR, Europe, and North America. Similarly, HIV-1 A1 env sequences were from countries located in Asia, Oceania, Europe, North America, Africa, and Sub-Saharan Africa, while A6 sequences were from countries located in Former USSR and Europe.
(DOCX)

S3 Table Frequency of all Pol and Env residues, including signature mutations between subtypes A1 and A6. A1 and A6 sequences were compared using VESPA. A1 and A6 sequences were used as background and query, respectively.
(DOCX)
