# Supplementary figures and images for: Origin and evolution of HIV-1 subtype A6
Source: PLoS One. 2021 Dec 13;16(12):e0260604. doi: 10.1371/journal.pone.0260604 (PMC8668117; doi:10.1371/journal.pone.0260604)

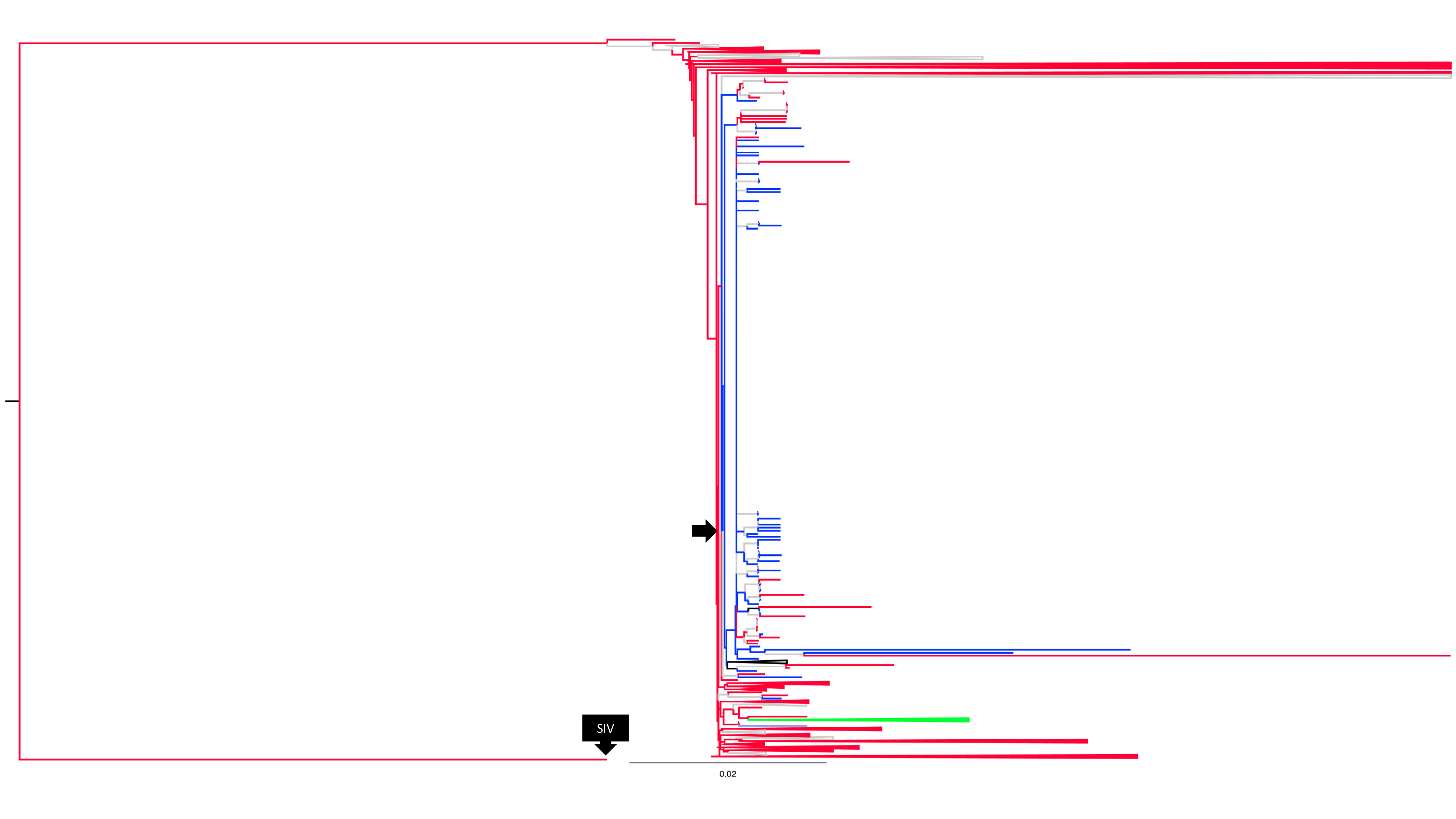

Supplement: S1 Fig — Grey-colored branches signify ≥0.9 support value. For clarity, unrelated subtype A1 clusters have been collapsed. The black arrow indicates the A6 ancestral node shared by A1 sequences. SIV gag sequence from chimpanzee was used as an outlier to root the tree. (TIFF) [file pone.0260604.s001.tiff]

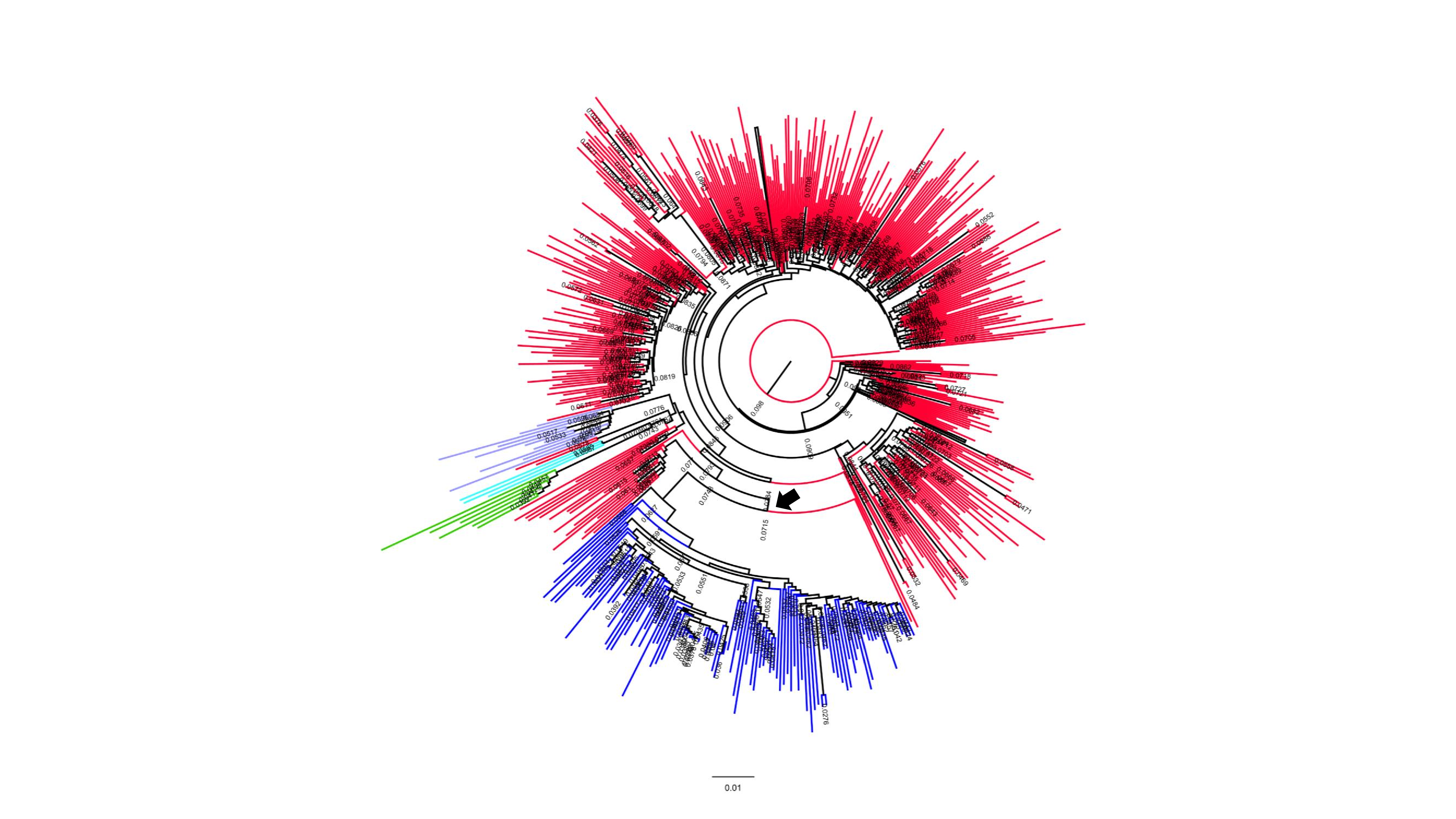

Supplement: S2 Fig — HIV-1A sub-subtypes are shown in red (A1), green (A2), purple (A3), turquoise (A4), blue (A6) colors. The black arrow indicates the A6 ancestral node shared by A1 sequences. Values on the nodes show genetic distances. (TIFF) [file pone.0260604.s002.tiff]
