# Supplementary material for: Origin and evolution of HIV-1 subtype A6
Source: PLoS One. 2021 Dec 13;16(12):e0260604. doi: 10.1371/journal.pone.0260604 (PMC8668117; doi:10.1371/journal.pone.0260604)
Supplement: S1 Table — (DOCX) [file pone.0260604.s003.docx]

**S1 Table**

| Subtypes | Final number of sequences used | |
| --- | --- | --- |
| A1 | 420 | |
| A2 | 6 | |
| A3 | 11 | |
| A4 | 3 | |
| A6 | 112 | |
|  |  |  |
| Subtypes | Countries | Sample date |
| A1 | AU | 2003 |
|  | BE | 2000 |
|  | BE | 2001 |
|  | BE | 2003 |
|  | CA | - |
|  | CD | 2002 |
|  | CH | 2003 |
|  | CM | 1997 |
|  | CM | 1997 |
|  | CM | 1997 |
|  | CM | 1999 |
|  | CM | 1999 |
|  | CM | 2003 |
|  | CM | 2007 |
|  | CM | 2007 |
|  | CM | 2008 |
|  | CY | 2005 |
|  | CY | 2005 |
|  | CY | 2005 |
|  | CY | 2005 |
|  | CY | 2005 |
|  | CY | 2005 |
|  | CY | 2005 |
|  | CY | 2005 |
|  | CY | 2006 |
|  | CY | 2006 |
|  | CY | 2007 |
|  | CY | 2007 |
|  | CY | 2007 |
|  | CY | 2007 |
|  | CY | 2008 |
|  | CY | 2008 |
|  | CY | 2008 |
|  | CY | 2008 |
|  | CY | 2009 |
|  | ES | 2005 |
|  | ES | 2006 |
|  | ES | 2015 |
|  | GB | 2013 |
|  | GB | 2013 |
|  | GB | 2013 |
|  | GB | 2014 |
|  | GB | 2014 |
|  | GB | 2014 |
|  | GB | 2014 |
|  | IN | 1997 |
|  | IN | 1999 |
|  | IN | 1999 |
|  | IN | 1999 |
|  | IN | 2000 |
|  | IN | 2004 |
|  | IN | 2009 |
|  | IN | 2009 |
|  | IN | 2010 |
|  | KE | 1986 |
|  | KE | 1986 |
|  | KE | 1994 |
|  | KE | 1997 |
|  | KE | 1997 |
|  | KE | 1999 |
|  | KE | 1999 |
|  | KE | 1999 |
|  | KE | 2000 |
|  | KE | 2000 |
|  | KE | 2000 |
|  | KE | 2000 |
|  | KE | 2000 |
|  | KE | 2000 |
|  | KE | 2000 |
|  | KE | 2000 |
|  | KE | 2000 |
|  | KE | 2000 |
|  | KE | 2000 |
|  | KE | 2000 |
|  | KE | 2000 |
|  | KE | 2000 |
|  | KE | 2000 |
|  | KE | 2000 |
|  | KE | 2000 |
|  | KE | 2001 |
|  | KE | 2001 |
|  | KE | 2001 |
|  | KE | 2001 |
|  | KE | 2002 |
|  | KE | 2002 |
|  | KE | 2002 |
|  | KE | 2002 |
|  | KE | 2002 |
|  | KE | 2004 |
|  | KE | 2004 |
|  | KE | 2004 |
|  | KE | 2004 |
|  | KE | 2004 |
|  | KE | 2004 |
|  | KE | 2004 |
|  | KE | 2004 |
|  | KE | 2005 |
|  | KE | 2005 |
|  | KE | 2005 |
|  | KE | 2005 |
|  | KE | 2005 |
|  | KE | 2005 |
|  | KE | 2005 |
|  | KE | 2006 |
|  | KE | 2006 |
|  | KE | 2006 |
|  | KE | 2006 |
|  | KE | 2006 |
|  | KE | 2006 |
|  | KE | 2006 |
|  | KE | 2006 |
|  | KE | 2006 |
|  | KE | 2006 |
|  | KE | 2006 |
|  | KE | 2006 |
|  | KE | 2006 |
|  | KE | 2006 |
|  | KE | 2006 |
|  | KE | 2006 |
|  | KE | 2006 |
|  | KE | 2006 |
|  | KE | 2006 |
|  | KE | 2006 |
|  | KE | 2011 |
|  | KE | 2011 |
|  | PK | 2014 |
|  | PK | 2014 |
|  | PK | 2014 |
|  | PK | 2014 |
|  | PK | 2014 |
|  | PK | 2014 |
|  | PK | 2014 |
|  | PK | 2014 |
|  | PK | 2014 |
|  | PK | 2014 |
|  | PK | 2014 |
|  | PK | 2015 |
|  | PK | 2015 |
|  | PK | 2015 |
|  | PK | 2015 |
|  | PK | 2015 |
|  | PK | 2015 |
|  | RW | 1992 |
|  | RW | 1992 |
|  | RW | 1993 |
|  | RW | 1993 |
|  | RW | 2006 |
|  | RW | 2007 |
|  | RW | 2007 |
|  | RW | 2007 |
|  | RW | 2007 |
|  | RW | 2008 |
|  | RW | 2011 |
|  | SE | 1993 |
|  | SE | 1994 |
|  | SE | 1994 |
|  | SE | 1995 |
|  | SE | 1995 |
|  | SE | 1995 |
|  | SE | 2012 |
|  | SE | 2012 |
|  | SE | 2013 |
|  | SE | 2013 |
|  | SN | 1998 |
|  | TZ | 1997 |
|  | TZ | 1997 |
|  | TZ | 2001 |
|  | TZ | 2001 |
|  | TZ | 2002 |
|  | TZ | 2003 |
|  | TZ | 2004 |
|  | TZ | 2004 |
|  | TZ | 2004 |
|  | TZ | 2004 |
|  | TZ | 2004 |
|  | TZ | 2005 |
|  | TZ | 2005 |
|  | TZ | 2005 |
|  | TZ | 2005 |
|  | TZ | 2005 |
|  | TZ | 2005 |
|  | TZ | 2006 |
|  | TZ | 2006 |
|  | TZ | 2006 |
|  | TZ | 2006 |
|  | TZ | 2006 |
|  | TZ | 2008 |
|  | TZ | 2008 |
|  | TZ | 2008 |
|  | UG | - |
|  | UG | - |
|  | UG | - |
|  | UG | 1985 |
|  | UG | 1992 |
|  | UG | 1992 |
|  | UG | 1998 |
|  | UG | 1998 |
|  | UG | 1998 |
|  | UG | 1998 |
|  | UG | 1999 |
|  | UG | 1999 |
|  | UG | 2002 |
|  | UG | 2002 |
|  | UG | 2002 |
|  | UG | 2002 |
|  | UG | 2002 |
|  | UG | 2002 |
|  | UG | 2002 |
|  | UG | 2002 |
|  | UG | 2002 |
|  | UG | 2002 |
|  | UG | 2002 |
|  | UG | 2002 |
|  | UG | 2002 |
|  | UG | 2002 |
|  | UG | 2002 |
|  | UG | 2002 |
|  | UG | 2002 |
|  | UG | 2002 |
|  | UG | 2002 |
|  | UG | 2002 |
|  | UG | 2002 |
|  | UG | 2005 |
|  | UG | 2005 |
|  | UG | 2005 |
|  | UG | 2005 |
|  | UG | 2005 |
|  | UG | 2005 |
|  | UG | 2005 |
|  | UG | 2005 |
|  | UG | 2005 |
|  | UG | 2005 |
|  | UG | 2005 |
|  | UG | 2005 |
|  | UG | 2005 |
|  | UG | 2005 |
|  | UG | 2005 |
|  | UG | 2005 |
|  | UG | 2005 |
|  | UG | 2005 |
|  | UG | 2005 |
|  | UG | 2006 |
|  | UG | 2006 |
|  | UG | 2006 |
|  | UG | 2006 |
|  | UG | 2006 |
|  | UG | 2006 |
|  | UG | 2006 |
|  | UG | 2006 |
|  | UG | 2006 |
|  | UG | 2006 |
|  | UG | 2006 |
|  | UG | 2006 |
|  | UG | 2006 |
|  | UG | 2006 |
|  | UG | 2006 |
|  | UG | 2006 |
|  | UG | 2006 |
|  | UG | 2006 |
|  | UG | 2006 |
|  | UG | 2006 |
|  | UG | 2006 |
|  | UG | 2006 |
|  | UG | 2006 |
|  | UG | 2006 |
|  | UG | 2006 |
|  | UG | 2006 |
|  | UG | 2006 |
|  | UG | 2006 |
|  | UG | 2006 |
|  | UG | 2006 |
|  | UG | 2006 |
|  | UG | 2006 |
|  | UG | 2006 |
|  | UG | 2006 |
|  | UG | 2006 |
|  | UG | 2006 |
|  | UG | 2006 |
|  | UG | 2006 |
|  | UG | 2006 |
|  | UG | 2006 |
|  | UG | 2006 |
|  | UG | 2006 |
|  | UG | 2006 |
|  | UG | 2006 |
|  | UG | 2006 |
|  | UG | 2006 |
|  | UG | 2006 |
|  | UG | 2006 |
|  | UG | 2007 |
|  | UG | 2007 |
|  | UG | 2007 |
|  | UG | 2007 |
|  | UG | 2007 |
|  | UG | 2007 |
|  | UG | 2007 |
|  | UG | 2007 |
|  | UG | 2007 |
|  | UG | 2007 |
|  | UG | 2007 |
|  | UG | 2007 |
|  | UG | 2007 |
|  | UG | 2007 |
|  | UG | 2007 |
|  | UG | 2007 |
|  | UG | 2007 |
|  | UG | 2007 |
|  | UG | 2007 |
|  | UG | 2007 |
|  | UG | 2007 |
|  | UG | 2007 |
|  | UG | 2007 |
|  | UG | 2007 |
|  | UG | 2007 |
|  | UG | 2007 |
|  | UG | 2007 |
|  | UG | 2007 |
|  | UG | 2007 |
|  | UG | 2007 |
|  | UG | 2007 |
|  | UG | 2007 |
|  | UG | 2007 |
|  | UG | 2007 |
|  | UG | 2007 |
|  | UG | 2007 |
|  | UG | 2007 |
|  | UG | 2007 |
|  | UG | 2007 |
|  | UG | 2007 |
|  | UG | 2007 |
|  | UG | 2007 |
|  | UG | 2007 |
|  | UG | 2007 |
|  | UG | 2007 |
|  | UG | 2007 |
|  | UG | 2007 |
|  | UG | 2007 |
|  | UG | 2007 |
|  | UG | 2007 |
|  | UG | 2007 |
|  | UG | 2007 |
|  | UG | 2007 |
|  | UG | 2007 |
|  | UG | 2007 |
|  | UG | 2007 |
|  | UG | 2007 |
|  | UG | 2007 |
|  | UG | 2007 |
|  | UG | 2007 |
|  | UG | 2007 |
|  | UG | 2007 |
|  | UG | 2007 |
|  | UG | 2007 |
|  | UG | 2007 |
|  | UG | 2007 |
|  | UG | 2007 |
|  | UG | 2007 |
|  | UG | 2007 |
|  | UG | 2007 |
|  | UG | 2007 |
|  | UG | 2007 |
|  | UG | 2007 |
|  | UG | 2007 |
|  | UG | 2007 |
|  | UG | 2008 |
|  | UG | 2008 |
|  | UG | 2008 |
|  | UG | 2008 |
|  | UG | 2008 |
|  | UG | 2008 |
|  | UG | 2008 |
|  | UG | 2008 |
|  | UG | 2008 |
|  | UG | 2008 |
|  | UG | 2008 |
|  | UG | 2008 |
|  | UG | 2008 |
|  | UG | 2008 |
|  | UG | 2008 |
|  | UG | 2008 |
|  | UG | 2008 |
|  | UG | 2008 |
|  | UG | 2008 |
|  | UG | 2008 |
|  | UG | 2008 |
|  | UG | 2008 |
|  | UG | 2008 |
|  | UG | 2008 |
|  | UG | 2008 |
|  | UG | 2008 |
|  | UG | 2008 |
|  | UG | 2008 |
|  | UG | 2008 |
|  | UG | 2008 |
|  | UG | 2008 |
|  | UG | 2008 |
|  | UG | 2008 |
|  | UG | 2008 |
|  | UG | 2009 |
|  | UG | 2009 |
|  | UG | 2009 |
|  | UG | 2009 |
|  | UG | 2009 |
|  | UG | 2009 |
|  | UG | 2009 |
|  | UG | 2009 |
|  | UG | 2009 |
|  | UG | 2009 |
|  | UG | 2009 |
|  | UG | 2009 |
|  | UG | 2009 |
|  | UG | 2009 |
|  | UG | 2009 |
|  | UG | 2010 |
|  | UG | 2011 |
|  | UG | 2011 |
|  | ZA | 2000 |
|  | ZA | 2000 |
|  | ZA | 2001 |
|  | ZA | 2004 |
| A2 | CD | 1987 |
|  | CD | 1997 |
|  | CD | 1997 |
|  | CM | 2001 |
|  | CY | 1994 |
|  | ZM | - |
| A3 | BF | - |
|  | GB | 2013 |
|  | GQ | 2008 |
|  | GQ | 2008 |
|  | ML | - |
|  | ML | - |
|  | SN | 1996 |
|  | SN | 2001 |
|  | SN | 2001 |
|  | TG | 2006 |
|  | US | - |
| A4 | CD | 1997 |
|  | CD | 1997 |
|  | CD | 2002 |
| A6 | BY | 1997 |
|  | BY | 2013 |
|  | CY | 2005 |
|  | CY | 2005 |
|  | CY | 2006 |
|  | CY | 2006 |
|  | CY | 2007 |
|  | CY | 2008 |
|  | CY | 2009 |
|  | GB | 2013 |
|  | GE | 1999 |
|  | IT | 2002 |
|  | KZ | 2002 |
|  | KZ | 2002 |
|  | KZ | 2002 |
|  | KZ | 2002 |
|  | KZ | 2002 |
|  | KZ | 2002 |
|  | RU | - |
|  | RU | 2000 |
|  | RU | 2002 |
|  | RU | 2003 |
|  | RU | 2005 |
|  | RU | 2006 |
|  | RU | 2006 |
|  | RU | 2006 |
|  | RU | 2006 |
|  | RU | 2006 |
|  | RU | 2007 |
|  | RU | 2008 |
|  | RU | 2008 |
|  | RU | 2008 |
|  | RU | 2008 |
|  | RU | 2008 |
|  | RU | 2008 |
|  | RU | 2010 |
|  | RU | 2010 |
|  | RU | 2011 |
|  | RU | 2012 |
|  | RU | 2012 |
|  | RU | 2012 |
|  | RU | 2012 |
|  | RU | 2012 |
|  | RU | 2012 |
|  | RU | 2012 |
|  | RU | 2012 |
|  | RU | 2012 |
|  | RU | 2012 |
|  | RU | 2012 |
|  | RU | 2012 |
|  | RU | 2012 |
|  | RU | 2012 |
|  | RU | 2012 |
|  | RU | 2012 |
|  | RU | 2012 |
|  | RU | 2013 |
|  | RU | 2013 |
|  | RU | 2013 |
|  | RU | 2013 |
|  | RU | 2013 |
|  | RU | 2013 |
|  | RU | 2013 |
|  | RU | 2013 |
|  | RU | 2013 |
|  | RU | 2013 |
|  | RU | 2013 |
|  | RU | 2013 |
|  | RU | 2013 |
|  | RU | 2013 |
|  | RU | 2013 |
|  | RU | 2013 |
|  | RU | 2013 |
|  | RU | 2013 |
|  | RU | 2013 |
|  | RU | 2013 |
|  | RU | 2013 |
|  | RU | 2014 |
|  | RU | 2014 |
|  | RU | 2014 |
|  | TJ | 2017 |
|  | TJ | 2017 |
|  | TJ | 2017 |
|  | TJ | 2017 |
|  | TJ | 2017 |
|  | TJ | 2017 |
|  | UA | 2000 |
|  | UA | 2001 |
|  | UA | 2001 |
|  | UA | 2001 |
|  | UA | 2001 |
|  | UA | 2001 |
|  | UA | 2001 |
|  | UA | 2001 |
|  | UA | 2001 |
|  | UA | 2001 |
|  | UA | 2011 |
|  | UA | 2011 |
|  | UA | 2011 |
|  | UA | 2012 |
|  | UA | 2012 |
|  | UA | 2012 |
|  | UA | 2012 |
|  | UA | 2012 |
|  | UA | 2012 |
|  | UZ | 2002 |
|  | UZ | 2002 |
|  | UZ | 2002 |
|  | UZ | 2002 |
|  | UZ | 2002 |
|  | UZ | 2002 |
|  | UZ | 2002 |
|  | UZ | 2002 |
| A8 | Cabo Verde | 2010-2011 |
