# Supplementary material for: Origin and evolution of HIV-1 subtype A6
Source: PLoS One. 2021 Dec 13;16(12):e0260604. doi: 10.1371/journal.pone.0260604 (PMC8668117; doi:10.1371/journal.pone.0260604)
Supplement: S2 Table — HIV-1 A1 pol sequences were from countries located in South America, the Middle East, Asia, Caribbean, Oceania, Former USSR, Europe, North America, Africa, Sub-Saharan Africa, while HIV A6 pol sequences were from countries located in the Middle East, Asia, Oceania, Former USSR, Europe, and North America. Similarly, HIV-1 A1 env sequences were from countries located in Asia, Oceania, Europe, North America, Africa, and Sub-Saharan Africa, while A6 sequences were from countries located in Former USSR and Europe. (DOCX) [file pone.0260604.s004.docx]

**S2 Table**

| A1 | KX128992 |
| --- | --- |
|  | KX129002 |
|  | KX128984 |
|  | KX128988 |
|  | KX128987 |
|  | KX128989 |
|  | KX128990 |
|  | KX128993 |
|  | KX129003 |
|  | KX128995 |
|  | KX129005 |
|  | KX129004 |
|  | KX129006 |
|  | KX129007 |
|  | KX128986 |
|  | KX128991 |
|  | KX129001 |
|  | KX128983 |
|  | KX128982 |
|  | HQ179655 |
|  | GU582000 |
|  | GU582052 |
|  | EU606759 |
|  | EU607538 |
|  | EU609876 |
|  | EU610550 |
|  | EU611422 |
|  | EU612079 |
|  | EU612281 |
|  | EU613045 |
|  | EU613119 |
|  | EU615188 |
|  | EU615324 |
|  | EU615784 |
|  | JN631016 |
|  | JN631019 |
|  | JN631020 |
|  | JN631021 |
|  | JN630985 |
|  | JN630988 |
|  | JN630989 |
|  | JN630990 |
|  | AY611702 |
|  | AY611698 |
|  | AY611710 |
|  | AY611711 |
|  | AY611703 |
|  | AY611699 |
|  | AY611704 |
|  | AY611705 |
|  | AY611695 |
|  | AY611700 |
|  | AY611712 |
|  | AY611709 |
|  | AY611713 |
|  | AY611714 |
|  | AY611654 |
|  | AY611691 |
|  | AY611692 |
|  | AY611655 |
|  | AY611656 |
|  | AY611715 |
|  | AY611694 |
|  | AY611657 |
|  | AY611689 |
|  | AY611690 |
|  | AY611658 |
|  | AY611659 |
|  | AY611652 |
|  | AY611653 |
|  | AY611696 |
|  | AY611660 |
|  | AY611697 |
|  | AY611662 |
|  | AY611706 |
|  | AY611663 |
|  | AY611664 |
|  | AY611707 |
|  | MK543533 |
|  | MK543541 |
|  | MK543542 |
|  | GQ399325 |
|  | GQ399696 |
|  | GQ399911 |
|  | GQ399917 |
|  | GQ400063 |
|  | GQ400175 |
|  | GQ400003 |
|  | JX299538 |
|  | JX300100 |
|  | JX300367 |
|  | JX300659 |
|  | DQ676872 |
|  | KC238092 |
|  | DQ676873 |
|  | KC237942 |
|  | KC238296 |
|  | KC238027 |
|  | KC238028 |
|  | KC238048 |
|  | KC238058 |
|  | KJ438641 |
|  | AF338999 |
|  | KJ438643 |
|  | AJ634690 |
|  | DQ877748 |
|  | DQ877778 |
|  | DQ877779 |
|  | EU248305 |
|  | EU248311 |
|  | EU248322 |
|  | EU248323 |
|  | EU248357 |
|  | EU248383 |
|  | EU248388 |
|  | EU248389 |
|  | EU248426 |
|  | EU248433 |
|  | EU248454 |
|  | EU248465 |
|  | EU248512 |
|  | EU248520 |
|  | EU248562 |
|  | EU248552 |
|  | EU248556 |
|  | EU248580 |
|  | EU248588 |
|  | DQ877780 |
|  | JX300549 |
|  | JX300785 |
|  | JX300883 |
|  | JX299670 |
|  | JX300512 |
|  | JX300641 |
|  | KJ936923 |
|  | KJ936924 |
|  | KY386799 |
|  | MH746259 |
|  | MH746258 |
|  | MH746253 |
|  | AM260289 |
|  | AM260313 |
|  | AM260315 |
|  | AM260322 |
|  | AM260224 |
|  | AM260240 |
|  | AM260254 |
|  | HG780855 |
|  | HG780761 |
|  | HG780783 |
|  | HG780762 |
|  | HG780802 |
|  | HG780763 |
|  | HG780823 |
|  | HG780756 |
|  | HG780768 |
|  | HG780841 |
|  | HG780649 |
|  | HG780673 |
|  | HG780710 |
|  | HG780681 |
|  | HG780618 |
|  | HG780626 |
|  | HG780616 |
|  | HG780627 |
|  | HQ267574 |
|  | HM534045 |
|  | MF278438 |
|  | DQ380550 |
|  | GQ303792 |
|  | EF368675 |
|  | EF368909 |
|  | EF368970 |
|  | EF369093 |
|  | EF369112 |
|  | KU189997 |
|  | KU189998 |
|  | KU189999 |
|  | KU190000 |
|  | KU190001 |
|  | KU190002 |
|  | KU189996 |
|  | KU190003 |
|  | KU190004 |
|  | KU190005 |
|  | KU190006 |
|  | KU190007 |
|  | KU190008 |
|  | KU190009 |
|  | KU190010 |
|  | KU190011 |
|  | KU190013 |
|  | KU190014 |
|  | KU190015 |
|  | KU190016 |
|  | KU190017 |
|  | KU190018 |
|  | KU190019 |
|  | KU190020 |
|  | KU190021 |
|  | KU190023 |
|  | KU190024 |
|  | KU190025 |
|  | KU190026 |
|  | KU190027 |
|  | KU190028 |
|  | KU190029 |
|  | KU190030 |
|  | AM041053 |
|  | AM041049 |
|  | AM040996 |
|  | KU168256 |
|  | FR666661 |
|  | FR666632 |
|  | FR666651 |
|  | FR666639 |
|  | FR666640 |
|  | FR666633 |
|  | FR666671 |
|  | FR666623 |
|  | FR666673 |
|  | FR666646 |
|  | FR666672 |
|  | FR666624 |
|  | FR666610 |
|  | FR666607 |
|  | FR666606 |
|  | FR666670 |
|  | FR666612 |
|  | FR666648 |
|  | FR666636 |
|  | KT315948 |
|  | KT315949 |
|  | KT315960 |
|  | KT315961 |
|  | KT315963 |
|  | KT315967 |
|  | KT315968 |
|  | KT315974 |
|  | KT315979 |
|  | KT315984 |
|  | KT315987 |
|  | KT315988 |
|  | KT315990 |
|  | KT315991 |
|  | KT315993 |
|  | KT315994 |
|  | KT316000 |
|  | KT316002 |
|  | KT315996 |
|  | KT315997 |
|  | JF804010 |
|  | JF803988 |
|  | LT578218 |
|  | LT578219 |
|  | LT578221 |
|  | LT578240 |
|  | LT578180 |
|  | LT578259 |
|  | LT578273 |
|  | LT578187 |
|  | LT578171 |
|  | LT578188 |
|  | LT578193 |
|  | LT578194 |
|  | LT578198 |
|  | LT578200 |
|  | LT578210 |
|  | LT578215 |
|  | FM164903 |
|  | KX691913 |
|  | KX692024 |
|  | KX691925 |
|  | KX691936 |
|  | KX691947 |
|  | KX691958 |
|  | KX692135 |
|  | KX692204 |
|  | KX692215 |
|  | KX692248 |
|  | KX692259 |
|  | GQ848149 |
|  | KX692047 |
|  | KX692058 |
|  | KX692069 |
|  | KX692080 |
|  | KX692102 |
|  | KX692124 |
|  | KX692136 |
|  | KX692147 |
|  | KX692169 |
|  | KX692191 |
|  | KX692200 |
|  | KX692201 |
|  | KX692202 |
|  | KX692203 |
|  | KX692205 |
|  | KX692206 |
|  | KX692208 |
|  | JF769785 |
|  | JF769801 |
|  | JF769806 |
|  | JF769791 |
|  | JF769813 |
|  | AY204310 |
|  | AY204318 |
|  | AY444189 |
|  | AY444190 |
|  | AY444192 |
|  | MK580576 |
|  | MK580577 |
|  | MK580567 |
|  | DQ166431 |
|  | AJ286930 |
|  | AM279370 |
|  | AM279393 |
|  | AM279371 |
|  | AJ286934 |
|  | AM279372 |
|  | AM279375 |
|  | DQ452648 |
|  | DQ452651 |
|  | GU207096 |
|  | GU207121 |
|  | GU207119 |
|  | GU207028 |
|  | GU207037 |
|  | GU207036 |
|  | GU207034 |
|  | AM279446 |
|  | KU168305 |
|  | GU366146 |
|  | GU366132 |
|  | GU366134 |
|  | GU366135 |
|  | FJ688226 |
|  | FJ688261 |
|  | GU191583 |
|  | JN639447 |
|  | KP718918 |
|  | JN639449 |
|  | JN639455 |
|  | JN639451 |
|  | JN639454 |
|  | JN639450 |
|  | HQ864323 |
|  | HQ864329 |
|  | HQ864373 |
|  | HQ864330 |
|  | KP718928 |
|  | KX894263 |
|  | JQ796172 |
|  | JQ796155 |
|  | JQ796105 |
|  | KC350321 |
|  | KC350170 |
|  | KU684945 |
|  | KU684957 |
|  | KF735816 |
|  | KU684959 |
|  | KU684927 |
|  | KU684885 |
|  | KX894163 |
|  | KX894190 |
|  | MF434888 |
|  | MF797134 |
|  | MF797124 |
|  | MF797221 |
|  | MF797135 |
|  | KU168814 |
|  | KU168793 |
|  | MK702047 |
|  | MK702050 |
|  | MK702056 |
|  | MK720354 |
|  | KX894228 |
|  | MF797452 |
|  | MF797453 |
|  | MF797489 |
|  | MF797507 |
|  | MF797505 |
|  | MF797509 |
|  | MF797506 |
|  | MF797508 |
|  | MK867703 |
|  | MK867737 |
|  | MK867742 |
|  | MK720352 |
|  | EF122563 |
|  | EF122512 |
|  | EF122561 |
|  | DQ113332 |
|  | JQ585357 |
|  | JQ585390 |
|  | JQ585511 |
|  | EU673378 |
|  | EU673379 |
|  | EU673381 |
|  | EU673384 |
|  | FJ388893 |
|  | FJ388894 |
|  | FJ388903 |
|  | FJ388909 |
|  | FJ388925 |
|  | FJ388932 |
|  | FJ388938 |
|  | EU673397 |
|  | FJ388942 |
|  | FJ388943 |
|  | FJ388946 |
|  | EU673409 |
|  | JF683737 |
|  | JF683759 |
|  | JF683760 |
|  | JF683761 |
|  | JX299577 |
|  | JX299787 |
|  | JX300787 |
|  | JX300850 |
|  | JX300978 |
|  | JF683767 |
|  | JF683779 |
|  | JF683782 |
|  | JF683783 |
|  | JF683789 |
|  | KJ635945 |
|  | KJ635949 |
|  | KJ635953 |
|  | KJ635954 |
|  | KJ635972 |
|  | KJ635995 |
|  | KJ636000 |
|  | KJ636002 |
|  | KJ636010 |
|  | KJ636016 |
|  | KJ636018 |
|  | JN229047 |
|  | EU672697 |
|  | FJ842303 |
|  | GQ999106 |
|  | HQ025849 |
|  | HQ025854 |
|  | HQ025865 |
|  | JN229106 |
|  | JN229049 |
|  | JN229144 |
|  | JN229164 |
|  | JN229088 |
|  | JN229125 |
|  | JN229187 |
|  | DQ974869 |
|  | DQ975032 |
|  | EU672528 |
|  | AY694286 |
|  | JX299669 |
|  | JX300527 |
|  | JX300674 |
|  | MH072259 |
|  | MH072525 |
|  | MH072544 |
|  | KJ769717 |
|  | KX465322 |
|  | KJ769718 |
|  | KX465326 |
|  | KX465327 |
|  | MH072277 |
|  | MH072341 |
|  | MH072617 |
|  | KJ769755 |
|  | KX465323 |
|  | MH072666 |
|  | KJ769817 |
|  | KX465435 |
|  | KJ769838 |
|  | KX465324 |
|  | GQ400160 |
|  | GQ400810 |
|  | GQ400862 |
|  | KJ769937 |
|  | KX465325 |
|  | KJ769938 |
|  | KX465328 |
|  | MH072426 |
|  | MH072648 |
|  | GQ400857 |
|  | KJ770832 |
|  | MH072192 |
|  | MH072256 |
|  | KJ770166 |
|  | KX465948 |
|  | KJ770288 |
|  | KJ770305 |
|  | MH072423 |
|  | MH072652 |
|  | MH072698 |
|  | JX300561 |
|  | KJ770364 |
|  | KX465949 |
|  | KJ770486 |
|  | KX466317 |
|  | KJ770543 |
|  | KX466353 |
|  | KJ770606 |
|  | KJ770611 |
|  | MH072529 |
|  | MH072973 |
|  | MH072991 |
|  | MH073014 |
|  | MH073023 |
|  | JX299772 |
|  | JX300737 |
|  | KJ770626 |
|  | KX466421 |
|  | KJ770732 |
|  | KX465950 |
|  | KJ770863 |
|  | KX466354 |
|  | MH072433 |
|  | MH072882 |
|  | MH072922 |
|  | MH073071 |
|  | KJ771066 |
|  | KX465951 |
|  | MH072012 |
|  | MH072047 |
|  | MH072377 |
|  | MH072682 |
|  | KY496524 |
|  | KJ771192 |
|  | KX466869 |
|  | MH072015 |
|  | MH072091 |
|  | MH072199 |
|  | KJ771541 |
|  | KX467040 |
|  | KJ771610 |
|  | KX466870 |
|  | MH072023 |
|  | KJ771735 |
|  | KX467136 |
|  | KJ771818 |
|  | KX466871 |
|  | MH073546 |
|  | MH072073 |
|  | AM933291 |
|  | AJ419430 |
|  | AJ419452 |
|  | AJ419454 |
|  | AJ419507 |
|  | AJ419509 |
|  | AJ419516 |
|  | GQ399097 |
|  | GQ399269 |
|  | GQ399536 |
|  | GQ399657 |
|  | GQ400020 |
|  | AM706404 |
|  | AM706403 |
|  | GQ399515 |
|  | GQ400067 |
|  | GQ400089 |
|  | GQ400201 |
|  | GQ400320 |
|  | GQ398860 |
|  | AM706405 |
|  | JX299695 |
|  | JX299841 |
|  | JX299878 |
|  | JX300845 |
|  | JX300677 |
|  | JX301031 |
|  | AY017467 |
|  | EF583193 |
|  | MF403234 |
|  | DQ009048 |
|  | JX428563 |
|  | JX428558 |
|  | JX140650 |
|  | GQ241047 |
|  | FJ670519 |
|  | EU255377 |
|  | JX428567 |
|  | KC340391 |
|  | JX140651 |
|  | GQ240965 |
|  | FJ670523 |
|  | EU545194 |
|  | KC340433 |
|  | MF403238 |
|  | MF403236 |
|  | JX271308 |
|  | JF929146 |
|  | GQ240990 |
|  | MK177775 |
|  | KC340460 |
|  | GU326181 |
|  | MF403223 |
|  | MK177778 |
|  | MK177779 |
|  | MF403219 |
|  | MK177715 |
|  | MK177780 |
|  | MF403227 |
|  | MF403208 |
|  | MF403216 |
|  | MF403228 |
|  | MK177677 |
|  | MK177679 |
|  | MK177782 |
|  | MK177785 |
|  | MK177787 |
|  | MK177788 |
|  | MK177789 |
|  | MF403235 |
|  | KU685561 |
|  | MK177680 |
|  | MK177681 |
|  | MN014503 |
|  | MK177682 |
|  | MK177683 |
|  | MK177685 |
|  | MN014506 |
|  | MK177686 |
|  | MK177724 |
|  | KY496622 |
|  | MN014504 |
|  | MK177688 |
|  | MK177690 |
|  | MK177691 |
|  | MK177692 |
|  | MK177736 |
|  | MK177794 |
|  | MK177795 |
|  | MK177654 |
|  | MK177655 |
|  | MK177656 |
|  | MK177668 |
|  | MK177693 |
|  | MK177694 |
|  | MK177695 |
|  | MK177750 |
|  | MK177754 |
|  | MK177829 |
|  | MK177756 |
|  | MK177657 |
|  | MK177658 |
|  | MK177671 |
|  | MK177698 |
|  | MK177699 |
|  | MK177700 |
|  | MK177797 |
|  | MK177798 |
|  | MK177799 |
|  | MK177800 |
|  | MK177801 |
|  | MK177803 |
|  | MK177804 |
|  | MK177673 |
|  | MK177805 |
|  | MK177806 |
|  | MK177807 |
|  | AB285785 |
|  | GQ398965 |
|  | JX301156 |
|  | AJ270557 |
|  | AJ287032 |
|  | AJ577738 |
|  | AJ577959 |
|  | AJ577996 |
|  | AJ577740 |
|  | FJ030651 |
|  | DQ878217 |
|  | AM903432 |
|  | AY140643 |
|  | AJ313405 |
|  | AJ313418 |
|  | HQ541875 |
|  | HQ541876 |
|  | HQ541881 |
|  | HQ541882 |
|  | HQ541892 |
|  | HQ541900 |
|  | HQ541902 |
|  | HQ541912 |
|  | HQ541921 |
|  | HQ541932 |
|  | HQ541941 |
|  | GQ462040 |
|  | GQ462067 |
|  | GQ462088 |
|  | GQ462094 |
|  | GQ462098 |
|  | GQ462100 |
|  | GQ462107 |
|  | GQ462109 |
|  | GQ462122 |
|  | GQ462129 |
|  | GQ462141 |
|  | GQ462144 |
|  | GQ462156 |
|  | GQ462164 |
|  | GQ462166 |
|  | GQ462172 |
|  | GQ462175 |
|  | GQ462183 |
|  | GQ462184 |
|  | GQ462193 |
|  | GQ462205 |
|  | GQ462207 |
|  | GQ462210 |
|  | GQ462215 |
|  | GQ462234 |
|  | GQ462251 |
|  | GQ462260 |
|  | GQ462281 |
|  | GQ462300 |
|  | GQ462333 |
|  | GQ462354 |
|  | GQ462388 |
|  | GQ462391 |
|  | GQ462403 |
|  | GQ462413 |
|  | GQ462416 |
|  | GQ462417 |
|  | GQ462440 |
|  | GQ462442 |
|  | GQ462456 |
|  | GQ462459 |
|  | GQ462463 |
|  | GQ462464 |
|  | GQ462501 |
|  | GQ462526 |
|  | GQ462528 |
|  | GQ462532 |
|  | DQ879078 |
|  | KU142977 |
|  | KU498403 |
|  | KU498404 |
|  | KU498405 |
|  | KU498407 |
|  | KU498412 |
|  | KU498414 |
|  | KU498434 |
|  | KU498435 |
|  | KU498437 |
|  | KU498443 |
|  | KU498449 |
|  | KU498452 |
|  | KU498455 |
|  | KU498460 |
|  | KU498464 |
|  | KU498319 |
|  | KU498325 |
|  | KU498327 |
|  | KU498331 |
|  | KU498334 |
|  | KU498337 |
|  | KU498342 |
|  | KU498350 |
|  | KU498307 |
|  | KU498353 |
|  | KU498359 |
|  | KU498362 |
|  | KU498367 |
|  | KU498368 |
|  | KU498369 |
|  | KU498370 |
|  | KU498372 |
|  | KU498373 |
|  | KU498376 |
|  | KU498379 |
|  | KU498389 |
|  | KU498390 |
|  | KU498391 |
|  | KU498395 |
|  | KU498397 |
|  | KU498398 |
|  | KU498303 |
|  | KU498408 |
|  | KU498409 |
|  | KU498410 |
|  | KU498411 |
|  | KU498413 |
|  | KU498417 |
|  | KU498418 |
|  | KU498419 |
|  | KU498420 |
|  | KU498421 |
|  | KU498314 |
|  | KU498422 |
|  | KU498424 |
|  | KU498425 |
|  | KU498427 |
|  | KU498428 |
|  | KU498436 |
|  | KU498439 |
|  | KU498440 |
|  | KU498441 |
|  | KU498442 |
|  | KU498447 |
|  | KU498448 |
|  | KU498450 |
|  | KU498453 |
|  | KU498456 |
|  | KU498457 |
|  | KU498458 |
|  | KU498459 |
|  | KU498461 |
|  | KU498320 |
|  | KU498304 |
|  | KU498323 |
|  | KU498324 |
|  | KU498326 |
|  | KU498329 |
|  | KU498330 |
|  | KU498305 |
|  | KU498332 |
|  | KU498335 |
|  | KU498336 |
|  | KU498338 |
|  | KU498339 |
|  | KU498340 |
|  | KU498341 |
|  | KU498343 |
|  | KU498344 |
|  | KU498345 |
|  | KU498346 |
|  | KU498349 |
|  | KU498351 |
|  | KU498352 |
|  | KU498354 |
|  | KU498355 |
|  | KU498356 |
|  | KU498357 |
|  | KU498308 |
|  | KU498363 |
|  | KU498364 |
|  | KU498365 |
|  | KU498309 |
|  | KU498374 |
|  | KU498377 |
|  | KU498380 |
|  | KU498381 |
|  | KU498310 |
|  | KU498384 |
|  | KU498385 |
|  | KU498388 |
|  | KU498311 |
|  | KU498392 |
|  | KU498393 |
|  | KU498394 |
|  | KU498462 |
|  | KU498322 |
|  | KU498347 |
|  | KU498361 |
|  | KU498371 |
|  | KU498375 |
|  | KU498386 |
|  | KU498400 |
|  | KX662412 |
|  | KX662409 |
|  | KX662405 |
|  | KX662401 |
|  | KX662394 |
|  | KX662391 |
|  | KX662390 |
|  | KX662388 |
|  | KX662381 |
|  | KX662379 |
|  | KX662375 |
|  | KX662414 |
|  | KX662410 |
|  | KX662406 |
|  | KX662404 |
|  | KX662402 |
|  | KX662397 |
|  | KX662393 |
|  | KX662392 |
|  | KX662385 |
|  | KX662384 |
|  | KX662373 |
|  | KX662413 |
|  | KX662411 |
|  | KX662407 |
|  | KX662403 |
|  | KX662383 |
|  | KX662382 |
|  | KX662377 |
|  | MF109424 |
|  | MF109502 |
|  | MF109677 |
|  | MF109679 |
|  | MF109427 |
|  | MF109530 |
|  | MF109613 |
|  | MF109623 |
|  | KT998240 |
|  | MK067032 |
|  | MK067039 |
|  | DQ878538 |
|  | DQ878557 |
|  | DQ878558 |
|  | DQ878561 |
|  | GQ399336 |
|  | GQ400043 |
|  | GQ400397 |
|  | MH757124 |
|  | MH757125 |
|  | MH757126 |
|  | MH757127 |
|  | MH757128 |
|  | MH757129 |
|  | MH757130 |
|  | MH757131 |
|  | MH757132 |
|  | MH757133 |
|  | MH757134 |
|  | MH757135 |
|  | MH757136 |
|  | MH757137 |
|  | MH757138 |
|  | MH757140 |
|  | KY927974 |
|  | KC016239 |
|  | KT152840 |
|  | KT152841 |
|  | KT152842 |
|  | KT152844 |
|  | KT152845 |
|  | KT152843 |
|  | KT152846 |
|  | KT152847 |
|  | EF186972 |
|  | DQ826634 |
|  | DQ826661 |
|  | EU158893 |
|  | EU447782 |
|  | HQ453391 |
|  | HQ456677 |
|  | KT152839 |
|  | JN639204 |
|  | KP307345 |
|  | KP307430 |
|  | KJ185199 |
|  | KJ185200 |
|  | KJ185205 |
|  | KJ185219 |
|  | KJ185223 |
|  | KJ185228 |
|  | KJ185233 |
|  | KJ185245 |
|  | KJ185256 |
|  | KJ185257 |
|  | KJ185258 |
|  | KJ185260 |
|  | KJ185266 |
|  | KJ185268 |
|  | KJ185270 |
|  | KJ185272 |
|  | KJ185282 |
|  | KJ185292 |
|  | KJ185294 |
|  | KJ185316 |
|  | KJ185317 |
|  | KJ185320 |
|  | KJ185322 |
|  | KJ185326 |
|  | KJ185334 |
|  | KJ185340 |
|  | KJ185353 |
|  | KJ185355 |
|  | KJ185366 |
|  | KR816094 |
|  | KT318921 |
|  | KY787065 |
|  | AY995410 |
|  | AF295285 |
|  | AF295286 |
|  | AY460121 |
|  | GQ400403 |
|  | GQ398848 |
|  | GQ399929 |
|  | GQ399750 |
|  | KY386726 |
|  | AB356204 |
|  | AB356216 |
|  | AB356272 |
|  | AB356290 |
|  | AB356304 |
|  | AB442239 |
|  | AB356407 |
|  | AB356480 |
|  | AB640471 |
|  | AB640202 |
|  | AB640243 |
|  | AB640397 |
|  | AB640398 |
|  | AB640429 |
|  | AB640535 |
|  | AB864008 |
|  | AB864049 |
|  | AB865268 |
|  | AB866914 |
|  | AB868632 |
|  | AB865491 |
|  | AB866183 |
|  | AB866255 |
|  | AB865751 |
|  | AB866341 |
|  | AB866379 |
|  | LC162594 |
|  | MN240772 |
|  | MN240777 |
|  | DQ136643 |
|  | DQ136633 |
|  | DQ136634 |
|  | DQ136635 |
|  | DQ136636 |
|  | DQ136637 |
|  | DQ136638 |
|  | DQ136639 |
|  | DQ136640 |
|  | DQ136641 |
|  | DQ136642 |
|  | DQ136644 |
|  | DQ136690 |
|  | DQ136691 |
|  | DQ136692 |
|  | DQ136693 |
|  | DQ136694 |
|  | DQ136695 |
|  | DQ136696 |
|  | DQ136697 |
|  | DQ136698 |
|  | DQ136699 |
|  | DQ136709 |
|  | DQ136742 |
|  | DQ136743 |
|  | DQ136744 |
|  | DQ136745 |
|  | DQ136746 |
|  | DQ136747 |
|  | DQ136748 |
|  | DQ136749 |
|  | DQ136750 |
|  | DQ136751 |
|  | DQ136752 |
|  | DQ136753 |
|  | DQ136754 |
|  | DQ136755 |
|  | DQ136761 |
|  | DQ136762 |
|  | DQ136763 |
|  | DQ136764 |
|  | DQ136765 |
|  | DQ136766 |
|  | DQ136767 |
|  | DQ136768 |
|  | DQ136769 |
|  | DQ136770 |
|  | DQ136771 |
|  | DQ136772 |
|  | DQ136773 |
|  | DQ136774 |
|  | DQ136649 |
|  | DQ136650 |
|  | DQ136651 |
|  | DQ136652 |
|  | DQ136653 |
|  | DQ136654 |
|  | DQ136656 |
|  | DQ136657 |
|  | DQ136659 |
|  | DQ136660 |
|  | DQ136661 |
|  | DQ136662 |
|  | DQ136663 |
|  | DQ136664 |
|  | DQ136665 |
|  | DQ136666 |
|  | DQ136667 |
|  | DQ136668 |
|  | DQ136794 |
|  | DQ136795 |
|  | AY322184 |
|  | AF539405 |
|  | AF004885 |
|  | AY492766 |
|  | AY492753 |
|  | AY492759 |
|  | AY322185 |
|  | AY322190 |
|  | AY322193 |
|  | AY492761 |
|  | AY492765 |
|  | AY492780 |
|  | AF457063 |
|  | AF457065 |
|  | AF457075 |
|  | AY492777 |
|  | AF457052 |
|  | AF457053 |
|  | AF457055 |
|  | AF457066 |
|  | AF457067 |
|  | AF457068 |
|  | AF457069 |
|  | AF457070 |
|  | AF457077 |
|  | AF457079 |
|  | AF457080 |
|  | AF457081 |
|  | AF457083 |
|  | AF457084 |
|  | AF457086 |
|  | AF457089 |
|  | EU110087 |
|  | EU110095 |
|  | EU110088 |
|  | EU110085 |
|  | EU110092 |
|  | EU110097 |
|  | EU110094 |
|  | KT022360 |
|  | KT022361 |
|  | KT022363 |
|  | KT022364 |
|  | KT022365 |
|  | KT022367 |
|  | KT022368 |
|  | KT022369 |
|  | HM164117 |
|  | HM164125 |
|  | KT022370 |
|  | KT022372 |
|  | KT022373 |
|  | KT022374 |
|  | KT022375 |
|  | KT022376 |
|  | KT022377 |
|  | HM164114 |
|  | KT022378 |
|  | KT022380 |
|  | KT022381 |
|  | KT022382 |
|  | KT022383 |
|  | KT022384 |
|  | FJ623487 |
|  | FJ623481 |
|  | FJ623475 |
|  | FJ623476 |
|  | FJ623480 |
|  | FJ623485 |
|  | FJ623483 |
|  | FJ623488 |
|  | FJ623478 |
|  | FJ623482 |
|  | FJ623477 |
|  | FJ623484 |
|  | FJ623486 |
|  | FJ623479 |
|  | HM164131 |
|  | HM164130 |
|  | HM164120 |
|  | HM164128 |
|  | HM164122 |
|  | KC018605 |
|  | KC018679 |
|  | KC018811 |
|  | KC018903 |
|  | KC018926 |
|  | KC018959 |
|  | KC019052 |
|  | KC019067 |
|  | KC019096 |
|  | KC019097 |
|  | KC516873 |
|  | KC516918 |
|  | KC516877 |
|  | KC516878 |
|  | KC516879 |
|  | KC516880 |
|  | KC516886 |
|  | KC516884 |
|  | KC516887 |
|  | KC516888 |
|  | KC516890 |
|  | KC516891 |
|  | KC516892 |
|  | KC516893 |
|  | KC516895 |
|  | KC516896 |
|  | KC516920 |
|  | KC516899 |
|  | KC516907 |
|  | KC516908 |
|  | KC516921 |
|  | KC516922 |
|  | KC516909 |
|  | KC516910 |
|  | KC516923 |
|  | KC516911 |
|  | KC516912 |
|  | KC516913 |
|  | KC516904 |
|  | KC516917 |
|  | KC516919 |
|  | KC517057 |
|  | KC516990 |
|  | KC516992 |
|  | KC516994 |
|  | KC516996 |
|  | KC516997 |
|  | KC516998 |
|  | KC516999 |
|  | KC517000 |
|  | KC517058 |
|  | KC517004 |
|  | KC517023 |
|  | KC517005 |
|  | KC517054 |
|  | KC517007 |
|  | KC517059 |
|  | KC517024 |
|  | KC517009 |
|  | KC517010 |
|  | KC517011 |
|  | KC517012 |
|  | KC517013 |
|  | KC517015 |
|  | KC517016 |
|  | KC517025 |
|  | KC517050 |
|  | KC517035 |
|  | KC517048 |
|  | KC517036 |
|  | KC517055 |
|  | KC517019 |
|  | KC517040 |
|  | KC517027 |
|  | KC517028 |
|  | KC517030 |
|  | KC517031 |
|  | KC517044 |
|  | KC517033 |
|  | KC517045 |
|  | KC517046 |
|  | KC517052 |
|  | KC517053 |
|  | KC517034 |
|  | KC517049 |
|  | KC516926 |
|  | KC516927 |
|  | KC516928 |
|  | KC516929 |
|  | KC516931 |
|  | KC516932 |
|  | KC516937 |
|  | KC516938 |
|  | KC516941 |
|  | KC516943 |
|  | KC516975 |
|  | KC516985 |
|  | KC516986 |
|  | KC516961 |
|  | KC516962 |
|  | KC516964 |
|  | KC516976 |
|  | KC516944 |
|  | KC516945 |
|  | KC516947 |
|  | KC516974 |
|  | KC516948 |
|  | KC516977 |
|  | KC516950 |
|  | KC516952 |
|  | KC516953 |
|  | KC516965 |
|  | KC516978 |
|  | KC516982 |
|  | KC516955 |
|  | KC516956 |
|  | KC516984 |
|  | KC516967 |
|  | KC516968 |
|  | KC516969 |
|  | KC516983 |
|  | KC516971 |
|  | HM164126 |
|  | HM164121 |
|  | HM164123 |
|  | HM164118 |
|  | KC018640 |
|  | KC018934 |
|  | KC018995 |
|  | KC019072 |
|  | HQ993572 |
|  | HQ993573 |
|  | HQ993575 |
|  | HQ993576 |
|  | HQ993581 |
|  | HQ993583 |
|  | HQ993586 |
|  | HQ993588 |
|  | HQ993589 |
|  | HQ993596 |
|  | HQ993598 |
|  | HQ993602 |
|  | HQ993603 |
|  | HQ993606 |
|  | HQ993607 |
|  | HQ993608 |
|  | HQ993609 |
|  | HQ993610 |
|  | HQ993613 |
|  | HQ993617 |
|  | HQ993618 |
|  | HQ993620 |
|  | HQ993621 |
|  | HQ993625 |
|  | HQ993627 |
|  | HQ993628 |
|  | HQ993630 |
|  | HQ993631 |
|  | HQ993633 |
|  | HQ993635 |
|  | HQ993636 |
|  | HQ993637 |
|  | HQ993639 |
|  | HQ993640 |
|  | HQ993642 |
|  | HQ993643 |
|  | HQ993645 |
|  | HQ993647 |
|  | HQ993649 |
|  | HQ993651 |
|  | JN630896 |
|  | HQ993652 |
|  | HQ993654 |
|  | HQ993655 |
|  | HQ993658 |
|  | HQ993661 |
|  | HQ993777 |
|  | HQ993780 |
|  | HQ993781 |
|  | HQ993782 |
|  | HQ993783 |
|  | HQ993785 |
|  | HQ993786 |
|  | HQ993790 |
|  | HQ993791 |
|  | HQ993792 |
|  | HQ993793 |
|  | HQ993794 |
|  | HQ993796 |
|  | HQ993798 |
|  | HQ993664 |
|  | HQ993800 |
|  | HQ993666 |
|  | HQ993667 |
|  | HQ993668 |
|  | HQ993671 |
|  | HQ993672 |
|  | HQ993675 |
|  | HQ993676 |
|  | HQ993679 |
|  | HQ993680 |
|  | HQ993683 |
|  | HQ993686 |
|  | HQ993687 |
|  | HQ993688 |
|  | HQ993691 |
|  | HQ993693 |
|  | HQ993695 |
|  | HQ993700 |
|  | HQ993703 |
|  | HQ993706 |
|  | HQ993708 |
|  | HQ993712 |
|  | HQ993713 |
|  | HQ993715 |
|  | HQ993716 |
|  | HQ993719 |
|  | HQ993720 |
|  | HQ993721 |
|  | HQ993722 |
|  | HQ993723 |
|  | HQ993724 |
|  | HQ993726 |
|  | HQ993727 |
|  | HQ993730 |
|  | HQ993731 |
|  | HQ993740 |
|  | HQ993741 |
|  | HQ993742 |
|  | HQ993743 |
|  | HQ993744 |
|  | HQ993745 |
|  | HQ993746 |
|  | HQ993747 |
|  | HQ993749 |
|  | HQ993752 |
|  | HQ993754 |
|  | HQ993756 |
|  | HQ993758 |
|  | HQ993761 |
|  | HQ993803 |
|  | HQ993804 |
|  | HQ993805 |
|  | HQ993809 |
|  | HQ993811 |
|  | HQ993762 |
|  | HQ993763 |
|  | HQ993764 |
|  | HQ993765 |
|  | HQ993812 |
|  | HQ993814 |
|  | HQ993815 |
|  | HQ993816 |
|  | HQ993817 |
|  | HQ993820 |
|  | HQ993821 |
|  | HQ993822 |
|  | HQ993767 |
|  | HQ993823 |
|  | JN630900 |
|  | HQ993825 |
|  | HQ993826 |
|  | HQ993768 |
|  | HQ993769 |
|  | JN630901 |
|  | HQ993770 |
|  | HQ993827 |
|  | HQ993828 |
|  | HQ993829 |
|  | HQ993830 |
|  | HQ993771 |
|  | HQ993774 |
|  | HQ993775 |
|  | HQ993832 |
|  | HQ993835 |
|  | HQ993839 |
|  | HQ993840 |
|  | HQ993841 |
|  | HQ993843 |
|  | HQ993845 |
|  | HQ993846 |
|  | HQ993847 |
|  | HQ993850 |
|  | HQ993851 |
|  | HQ993854 |
|  | HQ993855 |
|  | HQ993856 |
|  | HQ993857 |
|  | HQ993859 |
|  | HQ993860 |
|  | HQ993862 |
|  | HQ993864 |
|  | HQ993865 |
|  | HQ993866 |
|  | HQ993867 |
|  | HQ993870 |
|  | HQ993872 |
|  | HQ993874 |
|  | HQ993875 |
|  | HQ993876 |
|  | HQ993877 |
|  | HQ993878 |
|  | HQ993880 |
|  | HQ993882 |
|  | JN630904 |
|  | HQ993884 |
|  | HQ993885 |
|  | HQ993887 |
|  | HQ993888 |
|  | HQ993889 |
|  | HQ993890 |
|  | HQ993892 |
|  | HQ993894 |
|  | JQ480169 |
|  | HQ993897 |
|  | HQ993898 |
|  | HQ993901 |
|  | HQ993902 |
|  | HQ993904 |
|  | HQ993905 |
|  | HQ993906 |
|  | HQ993907 |
|  | HQ993908 |
|  | HQ993910 |
|  | HQ993911 |
|  | HQ993912 |
|  | HQ993915 |
|  | HQ993916 |
|  | HQ993919 |
|  | HQ993920 |
|  | HQ993923 |
|  | HQ993925 |
|  | JN630906 |
|  | HQ993928 |
|  | HQ993930 |
|  | HQ993931 |
|  | HQ993932 |
|  | HQ993933 |
|  | HQ993934 |
|  | HQ993935 |
|  | HQ993936 |
|  | JN630907 |
|  | HQ993939 |
|  | HQ993940 |
|  | HQ993941 |
|  | HQ993944 |
|  | HQ993945 |
|  | HQ993946 |
|  | HQ993950 |
|  | HQ993951 |
|  | HQ993953 |
|  | HQ993954 |
|  | HQ993956 |
|  | HQ993957 |
|  | HQ993958 |
|  | HQ993959 |
|  | JN630908 |
|  | HQ993961 |
|  | JN630909 |
|  | HQ993964 |
|  | HQ993965 |
|  | HQ993966 |
|  | JQ480170 |
|  | JN630910 |
|  | HQ993968 |
|  | HQ993970 |
|  | JQ480172 |
|  | JQ480175 |
|  | HQ993972 |
|  | JQ480176 |
|  | JQ480177 |
|  | JQ480179 |
|  | JQ480180 |
|  | JQ480181 |
|  | HQ993973 |
|  | HQ993974 |
|  | JQ480184 |
|  | JQ480186 |
|  | JQ480188 |
|  | JQ480190 |
|  | KC018567 |
|  | KC018948 |
|  | KC018953 |
|  | KC018961 |
|  | KC018962 |
|  | KC018966 |
|  | KC018991 |
|  | KC018963 |
|  | KC019083 |
|  | JN132272 |
|  | JN132273 |
|  | JN132274 |
|  | JN132302 |
|  | JN132304 |
|  | JN132362 |
|  | KC018542 |
|  | KC018559 |
|  | KC018571 |
|  | KC018573 |
|  | KC018578 |
|  | KC018634 |
|  | KC018658 |
|  | KC018750 |
|  | KC018868 |
|  | KC018885 |
|  | KC018897 |
|  | KC018918 |
|  | KC018919 |
|  | KC018751 |
|  | KC018950 |
|  | KC018951 |
|  | KC018969 |
|  | KC019000 |
|  | KC019003 |
|  | KC019043 |
|  | KC019099 |
|  | JN132359 |
|  | JN132360 |
|  | JN132361 |
|  | JN628461 |
|  | JN628464 |
|  | JN628465 |
|  | JN628467 |
|  | JN628468 |
|  | JN628470 |
|  | JN628471 |
|  | JN628473 |
|  | JN628477 |
|  | JN628478 |
|  | JN628482 |
|  | JN628485 |
|  | JN628486 |
|  | JN628488 |
|  | JN628489 |
|  | JN628491 |
|  | JN628492 |
|  | JN628494 |
|  | JN628495 |
|  | JN628496 |
|  | JN628497 |
|  | JN628498 |
|  | JN628501 |
|  | JN628502 |
|  | JN628503 |
|  | JN628504 |
|  | JN628505 |
|  | JN628506 |
|  | JN628509 |
|  | JN628510 |
|  | JN628511 |
|  | JN628512 |
|  | JN628513 |
|  | JN628516 |
|  | JN628520 |
|  | JN628522 |
|  | JN628523 |
|  | JN628524 |
|  | JN628526 |
|  | JN628528 |
|  | JN628529 |
|  | JN628530 |
|  | JN628532 |
|  | JQ625659 |
|  | JQ625658 |
|  | JQ625653 |
|  | JQ625651 |
|  | JQ625605 |
|  | JQ625631 |
|  | JQ625616 |
|  | KT370861 |
|  | KT370862 |
|  | KT370866 |
|  | KT370869 |
|  | KT370872 |
|  | KT370875 |
|  | KT370883 |
|  | KT370886 |
|  | KT370890 |
|  | KT370895 |
|  | KT370896 |
|  | KT370899 |
|  | KT370904 |
|  | KT370909 |
|  | KT370914 |
|  | KT370915 |
|  | KT370916 |
|  | KT370919 |
|  | JQ698350 |
|  | KT833391 |
|  | KT833478 |
|  | KT833393 |
|  | KC018519 |
|  | KC018538 |
|  | KC018541 |
|  | KC018544 |
|  | KC018621 |
|  | KC018642 |
|  | KC018645 |
|  | KC018651 |
|  | KC018699 |
|  | KC018767 |
|  | KC018770 |
|  | KC018842 |
|  | KC018845 |
|  | MF573215 |
|  | KC018904 |
|  | KC018913 |
|  | KC018921 |
|  | KC018922 |
|  | KC019002 |
|  | MF573217 |
|  | MF573218 |
|  | JN628466 |
|  | JN628483 |
|  | JN628519 |
|  | JQ625649 |
|  | JQ625640 |
|  | JQ625604 |
|  | JQ625607 |
|  | JQ625622 |
|  | JQ625629 |
|  | KT833476 |
|  | KC018584 |
|  | KC018624 |
|  | KC018723 |
|  | KC018726 |
|  | KC018749 |
|  | KC018853 |
|  | MF594802 |
|  | KC018977 |
|  | MF594804 |
|  | MG549751 |
|  | MG549763 |
|  | KC019106 |
|  | KF716474 |
|  | KF716475 |
|  | KR003380 |
|  | KR003381 |
|  | KR003383 |
|  | KR003384 |
|  | KR003386 |
|  | KR003387 |
|  | KR003389 |
|  | KR003390 |
|  | KR003392 |
|  | KP877944 |
|  | KP877945 |
|  | KP877946 |
|  | KP877888 |
|  | KP877921 |
|  | KP877891 |
|  | KP877893 |
|  | KP877897 |
|  | KP877927 |
|  | KP877928 |
|  | KP877929 |
|  | KP877930 |
|  | KP877934 |
|  | KP877935 |
|  | KP877936 |
|  | KP877900 |
|  | KP877901 |
|  | KP877902 |
|  | KP877903 |
|  | KP877905 |
|  | KP877906 |
|  | KP877908 |
|  | KP877911 |
|  | KP877912 |
|  | KP877914 |
|  | KP877917 |
|  | KP877954 |
|  | KP877955 |
|  | KR003400 |
|  | KR003397 |
|  | KR003398 |
|  | KR003405 |
|  | JQ625633 |
|  | KM016171 |
|  | MG549492 |
|  | MF594801 |
|  | MF573216 |
|  | MG549764 |
|  | MF573219 |
|  | KM016223 |
|  | MG549585 |
|  | MG549605 |
|  | MF573214 |
|  | MF594803 |
|  | MG597249 |
|  | MG597250 |
|  | MG597252 |
|  | MN240784 |
|  | KX155599 |
|  | KX155603 |
|  | JX299621 |
|  | HQ655139 |
|  | EU330722 |
|  | HG518335 |
|  | HG518384 |
|  | HG518346 |
|  | HG518348 |
|  | HG518363 |
|  | MF622564 |
|  | MF622565 |
|  | MF622724 |
|  | MF622768 |
|  | MF622769 |
|  | MF622780 |
|  | MF622792 |
|  | MF622823 |
|  | MF622903 |
|  | FJ931133 |
|  | FJ931153 |
|  | FJ931325 |
|  | HQ843590 |
|  | GQ399854 |
|  | GQ400099 |
|  | GQ400104 |
|  | GQ400121 |
|  | GQ400483 |
|  | GQ400523 |
|  | KY315758 |
|  | KM610170 |
|  | GQ400398 |
|  | GQ399334 |
|  | GQ400296 |
|  | GQ399832 |
|  | JX300650 |
|  | JQ430943 |
|  | JQ430985 |
|  | AB747487 |
|  | JQ011747 |
|  | JQ011748 |
|  | JQ011749 |
|  | JQ011751 |
|  | JQ011753 |
|  | JQ011754 |
|  | JQ011755 |
|  | JQ011756 |
|  | JQ011757 |
|  | JQ011758 |
|  | JQ011759 |
|  | JQ011760 |
|  | JQ011761 |
|  | JQ011762 |
|  | JQ011763 |
|  | JQ011765 |
|  | JQ011766 |
|  | JQ011767 |
|  | JQ011768 |
|  | JQ011769 |
|  | JQ011770 |
|  | JQ011771 |
|  | JQ011772 |
|  | JQ011773 |
|  | JQ011774 |
|  | JQ011776 |
|  | JQ011777 |
|  | JQ011779 |
|  | JQ011780 |
|  | JN620530 |
|  | JN620531 |
|  | JN620532 |
|  | JN620536 |
|  | JN620535 |
|  | JN620533 |
|  | JN620534 |
|  | JN620529 |
|  | JQ011625 |
|  | JQ011626 |
|  | JQ011628 |
|  | JQ011630 |
|  | JQ011632 |
|  | JQ011634 |
|  | JQ011637 |
|  | JQ011639 |
|  | JQ011642 |
|  | JQ011652 |
|  | JQ011654 |
|  | JQ011655 |
|  | JQ011656 |
|  | JQ011663 |
|  | JQ011664 |
|  | JQ011670 |
|  | JQ011673 |
|  | JQ011675 |
|  | JQ011676 |
|  | JQ011678 |
|  | JQ011681 |
|  | JQ011687 |
|  | JQ011688 |
|  | JQ011690 |
|  | JQ011691 |
|  | JQ011695 |
|  | JQ011699 |
|  | JQ011700 |
|  | JQ011702 |
|  | JQ011704 |
|  | JQ011709 |
|  | JQ011710 |
|  | JQ011714 |
|  | JQ011720 |
|  | JQ011721 |
|  | JQ011726 |
|  | JQ011729 |
|  | JQ011732 |
|  | JQ011733 |
|  | JQ011742 |
|  | JQ011743 |
|  | JN620520 |
|  | JN620507 |
|  | JN620509 |
|  | JN620510 |
|  | JN620499 |
|  | JN620500 |
|  | JN620526 |
|  | JN620518 |
|  | JN620519 |
|  | JN620527 |
|  | JN620504 |
|  | JN620514 |
|  | JN620505 |
|  | JN620506 |
|  | JN620525 |
|  | JN620501 |
|  | JN620502 |
|  | JN620503 |
|  | JN620521 |
|  | JN620522 |
|  | JN620524 |
|  | JN620512 |
|  | JN620517 |
|  | JN620511 |
|  | JN620523 |
|  | JN620516 |
|  | KU749409 |
|  | KU749410 |
|  | KU749411 |
|  | KY658714 |
|  | KX232594 |
|  | KX232595 |
|  | KX232597 |
|  | KX232600 |
|  | KX232606 |
|  | KX232607 |
|  | KX232609 |
|  | KX232610 |
|  | KX232611 |
|  | KX232613 |
|  | KX232619 |
|  | KY658715 |
|  | KY658716 |
|  | KX232614 |
|  | KX232618 |
|  | KX232620 |
|  | KX232621 |
|  | KX232624 |
|  | KX232626 |
|  | KM057329 |
|  | KM057341 |
|  | KM057343 |
|  | KM284664 |
|  | KM284418 |
|  | KT340128 |
|  | HQ700017 |
|  | HQ700065 |
|  | HQ700092 |
|  | HM102326 |
|  | HM102327 |
|  | GQ399625 |
|  | GQ400065 |
|  | GQ400427 |
|  | GQ398862 |
|  | HM102328 |
|  | KY386778 |
|  | GQ400401 |
|  | KY386677 |
|  | MK766258 |
|  | MK766263 |
|  | MK766266 |
|  | MK766267 |
|  | MK766262 |
|  | MK766265 |
|  | MK766260 |
|  | MK766261 |
|  | MK766257 |
|  | MK766264 |
|  | MK766268 |
|  | MK766256 |
|  | MK766259 |
|  | JX300370 |
|  | JX301114 |
|  | KF157525 |
|  | KF157489 |
|  | KF157491 |
|  | MK029129 |
|  | EU345848 |
|  | JX290226 |
|  | MH666607 |
|  | MK510025 |
|  | MK213240 |
|  | MK054191 |
|  | MK054192 |
|  | MK054193 |
|  | MK054194 |
|  | MK054195 |
|  | MK054197 |
|  | MK054198 |
|  | AB253422 |
|  | AB287377 |
|  | AY713406 |
|  | AB287379 |
|  | KF716499 |
|  | JQ364513 |
|  | JQ364515 |
|  | JQ364517 |
|  | JQ364518 |
|  | JQ364519 |
|  | JQ364520 |
|  | JQ364522 |
|  | JQ364523 |
|  | JQ364524 |
|  | JQ364525 |
|  | JQ364526 |
|  | JQ364528 |
|  | JQ364529 |
|  | JQ364530 |
|  | JQ364531 |
|  | JQ364532 |
|  | JQ364533 |
|  | JQ364535 |
|  | JQ364536 |
|  | JQ364538 |
|  | JQ364539 |
|  | JQ364540 |
|  | KC513702 |
|  | KC513704 |
|  | KC513706 |
|  | KC513707 |
|  | KC513708 |
|  | KC513709 |
|  | KC513712 |
|  | KC513713 |
|  | KC513714 |
|  | KC513715 |
|  | KC513716 |
|  | KC513717 |
|  | KC513718 |
|  | KC513719 |
|  | KC513720 |
|  | KC513721 |
|  | KC513722 |
|  | KC513724 |
|  | KC513725 |
|  | KC513727 |
|  | KC513728 |
|  | KC513729 |
|  | KC513730 |
|  | KC018517 |
|  | KC018540 |
|  | KC018543 |
|  | KC018659 |
|  | KC018671 |
|  | KC018736 |
|  | KC018794 |
|  | KC018827 |
|  | KC018906 |
|  | KC018923 |
|  | KC018956 |
|  | KC018972 |
|  | KC019021 |
|  | KC019042 |
|  | KC019059 |
|  | KC019062 |
|  | KC019095 |
|  | KC019105 |
|  | KU749423 |
|  | KC018524 |
|  | KC018728 |
|  | KC018739 |
|  | KC018760 |
|  | KC841661 |
|  | KC841662 |
|  | KC841664 |
|  | KC841665 |
|  | KC018792 |
|  | KC019005 |
|  | KC019026 |
|  | KC841726 |
|  | KC841727 |
|  | KC841728 |
|  | KC841729 |
|  | KC841730 |
|  | KC841731 |
|  | KC019086 |
|  | KP109528 |
|  | JX236677 |
|  | JX236678 |
|  | KJ190253 |
|  | KJ190254 |
|  | KJ190255 |
|  | KJ190256 |
|  | KJ190257 |
|  | KJ190258 |
|  | KJ190259 |
|  | KJ190260 |
|  | KJ190261 |
|  | KJ190262 |
|  | KP223729 |
|  | KP223730 |
|  | KP223731 |
|  | KP223732 |
|  | KP223733 |
|  | KP223734 |
|  | KP223735 |
|  | KP223736 |
|  | KP223737 |
|  | KP223738 |
|  | KP223739 |
|  | KP223740 |
|  | KP223741 |
|  | KP223742 |
|  | KP223743 |
|  | KP223744 |
|  | KP223745 |
|  | KP223746 |
|  | KP223747 |
|  | KP223748 |
|  | KP223749 |
|  | KP223750 |
|  | KP223751 |
|  | KP223752 |
|  | KP223753 |
|  | KP223754 |
|  | KP223755 |
|  | KP223756 |
|  | KP223757 |
|  | KP223758 |
|  | KP223768 |
|  | KP223769 |
|  | KP223770 |
|  | KP223771 |
|  | KP223772 |
|  | KP223773 |
|  | KP223774 |
|  | KP223775 |
|  | KP223776 |
|  | KP223777 |
|  | KP223778 |
|  | KP223779 |
|  | KP223780 |
|  | KP223781 |
|  | KJ190263 |
|  | KJ190264 |
|  | KJ190265 |
|  | KJ190266 |
|  | KJ190267 |
|  | KJ190268 |
|  | KJ190269 |
|  | KJ190270 |
|  | KJ190271 |
|  | KJ190273 |
|  | KJ190274 |
|  | KP223797 |
|  | KP223799 |
|  | KP223810 |
|  | KP223811 |
|  | KP223812 |
|  | KP223813 |
|  | KP223814 |
|  | KP223815 |
|  | KP223827 |
|  | KP223828 |
|  | KP223829 |
|  | KP223830 |
|  | KP223831 |
|  | KP223832 |
|  | KP223833 |
|  | KP223835 |
|  | KP223836 |
|  | KP223837 |
|  | KP223838 |
|  | KP223839 |
|  | KP223840 |
|  | KP223841 |
|  | KP223842 |
|  | KP223843 |
|  | KP223844 |
|  | KP223845 |
|  | KP223846 |
|  | KP223847 |
|  | KP223848 |
|  | KP223849 |
|  | KP223850 |
|  | KP223851 |
|  | KP223852 |
|  | KP223853 |
|  | KP223854 |
|  | KC018604 |
|  | KC018628 |
|  | KC841666 |
|  | KC841667 |
|  | KC841669 |
|  | KC841670 |
|  | KC841673 |
|  | KC841675 |
|  | KC841676 |
|  | KC841677 |
|  | KC841678 |
|  | KC841680 |
|  | KC841681 |
|  | KC841682 |
|  | KC841683 |
|  | KC018810 |
|  | KC841685 |
|  | KC841686 |
|  | KC841687 |
|  | KC841689 |
|  | KC841690 |
|  | KC841691 |
|  | KC841693 |
|  | KC841694 |
|  | KC841695 |
|  | KC841696 |
|  | KC841697 |
|  | KC841698 |
|  | KC841699 |
|  | KC841703 |
|  | KC841704 |
|  | KC841705 |
|  | KC841707 |
|  | KC841708 |
|  | KC841709 |
|  | KC018941 |
|  | KC018983 |
|  | KC019065 |
|  | KC841734 |
|  | KC841735 |
|  | KC841778 |
|  | KC841737 |
|  | KC841740 |
|  | KC841742 |
|  | KC841743 |
|  | KC841746 |
|  | KC841747 |
|  | KC841748 |
|  | KC841749 |
|  | KC841750 |
|  | KC841751 |
|  | KC841753 |
|  | KC841755 |
|  | KC019093 |
|  | KC841759 |
|  | KC841760 |
|  | KC841763 |
|  | KC841764 |
|  | KC841765 |
|  | KC019110 |
|  | KU749424 |
|  | KP223759 |
|  | KP223760 |
|  | KP223761 |
|  | KP223762 |
|  | KP223763 |
|  | KP223765 |
|  | KP223766 |
|  | KP223767 |
|  | KC018500 |
|  | KC018509 |
|  | KC018625 |
|  | KC018627 |
|  | KC018741 |
|  | KC018482 |
|  | KC841672 |
|  | KC841679 |
|  | KC841692 |
|  | KC841710 |
|  | KC841712 |
|  | KC841713 |
|  | KC841715 |
|  | KC841720 |
|  | KC841721 |
|  | KC841723 |
|  | KC841725 |
|  | KC018990 |
|  | KC019038 |
|  | KC841741 |
|  | KC841769 |
|  | KC841771 |
|  | KC841772 |
|  | KC841773 |
|  | KC841775 |
|  | KC841776 |
|  | KC841777 |
|  | KC019108 |
|  | KC018610 |
|  | KC018813 |
|  | KC841711 |
|  | KC841724 |
|  | KC018902 |
|  | KC018937 |
|  | KC019020 |
|  | KC841766 |
|  | KC018630 |
|  | KF716472 |
|  | KT982523 |
|  | KT982524 |
|  | KT982526 |
|  | KT982527 |
|  | KT982528 |
|  | KT982529 |
|  | KT982530 |
|  | KT982533 |
|  | KT982532 |
|  | KT982531 |
|  | AY102482 |
|  | AY102494 |
|  | MF373124 |
|  | AF069670 |
|  | AF069671 |
|  | AF069669 |
|  | AF107771 |
|  | AY165206 |
|  | AY165240 |
|  | AY165249 |
|  | GQ400536 |
|  | GQ400555 |
|  | GQ400575 |
|  | GQ400752 |
|  | GQ400781 |
|  | GQ400840 |
|  | JQ698799 |
|  | JQ698728 |
|  | GQ400883 |
|  | GQ399146 |
|  | GQ400350 |
|  | GU324872 |
|  | GU324875 |
|  | GQ400782 |
|  | GQ400951 |
|  | GU324881 |
|  | GU324886 |
|  | JX299809 |
|  | JX300064 |
|  | JQ698812 |
|  | JQ698862 |
|  | JX299753 |
|  | JX300248 |
|  | JX300580 |
|  | JX300654 |
|  | JX300966 |
|  | JX300987 |
|  | JQ698751 |
|  | JQ698759 |
|  | JQ698834 |
|  | JQ698676 |
|  | JQ698871 |
|  | JQ698689 |
|  | JQ698704 |
|  | KY386743 |
|  | JQ698793 |
|  | JQ698794 |
|  | JQ698874 |
|  | JQ698688 |
|  | JQ698785 |
|  | JQ698788 |
|  | JQ698813 |
|  | KY386691 |
|  | KY386759 |
|  | KY386744 |
|  | MF373167 |
|  | MF373168 |
|  | KY386736 |
|  | MF373181 |
|  | MF373182 |
|  | KP013648 |
|  | AJ286980 |
|  | AJ583737 |
|  | AJ286981 |
|  | FN599722 |
|  | AJ583720 |
|  | AJ583752 |
|  | FN599785 |
|  | HM002499 |
|  | HM002536 |
|  | KC350204 |
|  | HG424411 |
|  | GU191659 |
|  | GU191682 |
|  | KC350166 |
|  | KF745478 |
|  | KF745561 |
|  | MH543261 |
|  | KX183728 |
|  | KX183737 |
|  | KX183775 |
|  | KP121003 |
|  | KP121017 |
|  | KX775238 |
|  | KX775254 |
|  | KX775285 |
|  | KX775289 |
|  | AF361872 |
|  | AF361873 |
|  | AY253305 |
|  | AY253314 |
|  | KX907352 |
|  | KX907348 |
|  | KX907336 |
|  | KX907343 |
|  | KX907372 |
|  | KX907383 |
|  | KX907389 |
|  | JQ071458 |
|  | JQ071461 |
|  | JQ071466 |
|  | JQ071467 |
|  | JQ071469 |
|  | JQ071470 |
|  | JQ071473 |
|  | JQ071477 |
|  | JQ071479 |
|  | JQ071481 |
|  | JQ071453 |
|  | JQ071484 |
|  | JQ071454 |
|  | JQ071489 |
|  | JQ071455 |
|  | JQ071456 |
|  | KM438237 |
|  | KF531359 |
|  | KF530931 |
|  | KF530932 |
|  | KF530933 |
|  | KF530934 |
|  | KF530935 |
|  | KF530936 |
|  | KF530937 |
|  | KF530938 |
|  | KF530939 |
|  | KF530940 |
|  | KF530941 |
|  | KF530942 |
|  | KF530943 |
|  | KF530944 |
|  | KF530945 |
|  | KF530946 |
|  | KF530947 |
|  | KF530948 |
|  | KF530949 |
|  | KF530950 |
|  | KF530951 |
|  | KF530952 |
|  | KF530953 |
|  | KF530954 |
|  | KF530968 |
|  | KF530960 |
|  | KF530969 |
|  | KF530970 |
|  | KF530961 |
|  | KF530962 |
|  | KF530971 |
|  | KF530963 |
|  | KF530964 |
|  | KF530972 |
|  | KF530965 |
|  | KF530966 |
|  | KF530973 |
|  | KF530967 |
|  | KF530974 |
|  | KF530975 |
|  | KF530976 |
|  | KF530977 |
|  | KF530978 |
|  | KF530979 |
|  | KF530980 |
|  | KF530981 |
|  | KF530982 |
|  | KF530983 |
|  | KF530984 |
|  | KF530985 |
|  | KF530986 |
|  | KF530987 |
|  | KF531031 |
|  | KF531032 |
|  | KF531030 |
|  | KF531026 |
|  | KF531027 |
|  | KF531028 |
|  | KF531033 |
|  | KF531029 |
|  | KF531034 |
|  | KF531035 |
|  | KF531036 |
|  | KF531037 |
|  | KF531038 |
|  | KF531039 |
|  | KF531040 |
|  | KF531041 |
|  | KF531042 |
|  | KF531043 |
|  | KF531044 |
|  | KF531045 |
|  | KF531046 |
|  | KF531047 |
|  | KF531048 |
|  | KF531049 |
|  | KF531050 |
|  | KF531051 |
|  | KF531052 |
|  | KF531059 |
|  | KF531063 |
|  | KF531061 |
|  | KF531064 |
|  | KF531062 |
|  | KF531069 |
|  | KF531070 |
|  | KF531071 |
|  | KF531103 |
|  | KF531104 |
|  | KF531105 |
|  | KF531106 |
|  | KF531107 |
|  | KF531109 |
|  | KF531121 |
|  | KF531110 |
|  | KF531111 |
|  | KF531112 |
|  | KF531113 |
|  | KF531114 |
|  | KF531115 |
|  | KF531116 |
|  | KF531117 |
|  | KF531118 |
|  | KF531119 |
|  | KF531120 |
|  | KF531122 |
|  | KF531123 |
|  | KF531124 |
|  | KF531125 |
|  | KF531126 |
|  | KF531127 |
|  | KF531128 |
|  | KF531129 |
|  | KF531130 |
|  | KF531131 |
|  | KF531132 |
|  | KF531133 |
|  | KF531134 |
|  | KF531135 |
|  | KF531136 |
|  | KF531137 |
|  | KF531138 |
|  | KF531139 |
|  | KF531140 |
|  | KF531141 |
|  | KF531142 |
|  | KF531143 |
|  | KF531196 |
|  | KF531225 |
|  | KF531210 |
|  | KF531211 |
|  | KF531212 |
|  | KF531226 |
|  | KF531213 |
|  | KF531227 |
|  | KF531228 |
|  | KF531229 |
|  | KF531214 |
|  | KF531230 |
|  | KF531231 |
|  | KF531215 |
|  | KF531232 |
|  | KF531233 |
|  | KF531234 |
|  | KF531235 |
|  | KF531216 |
|  | KF531236 |
|  | KF531237 |
|  | KF531238 |
|  | KF531217 |
|  | KF531239 |
|  | KF531218 |
|  | KF531240 |
|  | KF531219 |
|  | KF531241 |
|  | KF531220 |
|  | KF531221 |
|  | KF531222 |
|  | KF531223 |
|  | KF531224 |
|  | KF531263 |
|  | KF531242 |
|  | KF531264 |
|  | KF531243 |
|  | KF531271 |
|  | KF531248 |
|  | KF531272 |
|  | KF531249 |
|  | KF531250 |
|  | KF531244 |
|  | KF531245 |
|  | KF531251 |
|  | KF531246 |
|  | KF531273 |
|  | KF531252 |
|  | KF531253 |
|  | KF531254 |
|  | KF531275 |
|  | KF531274 |
|  | KF531276 |
|  | KF531277 |
|  | KF531255 |
|  | KF531247 |
|  | KF531256 |
|  | KF531257 |
|  | KF531278 |
|  | KF531279 |
|  | KF531267 |
|  | KF531258 |
|  | KF531280 |
|  | KF531281 |
|  | KF531268 |
|  | KF531282 |
|  | KF531259 |
|  | KF531269 |
|  | KF531260 |
|  | KF531261 |
|  | KF531270 |
|  | KF531262 |
|  | KF531360 |
|  | KF531361 |
|  | KF531362 |
|  | KF531363 |
|  | KF531364 |
|  | KF531365 |
|  | KF531366 |
|  | KF531367 |
|  | KF531368 |
|  | KF531369 |
|  | KF531370 |
|  | KF531371 |
|  | KF531372 |
|  | KF531373 |
|  | KF531374 |
|  | KF531375 |
|  | KF531376 |
|  | KF531447 |
|  | KF531448 |
|  | KF531443 |
|  | KF531449 |
|  | KF531444 |
|  | KF531445 |
|  | KF531450 |
|  | KF531451 |
|  | KF531470 |
|  | KF531471 |
|  | KF531472 |
|  | KF531473 |
|  | KF531474 |
|  | KF531475 |
|  | KF531476 |
|  | KF531477 |
|  | KF531478 |
|  | KF531479 |
|  | KF531480 |
|  | KF531481 |
|  | KF531482 |
|  | KF531483 |
|  | KF531484 |
|  | KF531485 |
|  | KF531377 |
|  | KF531378 |
|  | KF531379 |
|  | KX907341 |
|  | KX907347 |
|  | KX907364 |
|  | KX907401 |
|  | KX907412 |
|  | KX228815 |
|  | EU251716 |
|  | EU251717 |
|  | EU251718 |
|  | EU251722 |
|  | EU251724 |
|  | EU251725 |
|  | EU251729 |
|  | EU251730 |
|  | EU251736 |
|  | EU251738 |
|  | EU251739 |
|  | EU251741 |
|  | EU251743 |
|  | EU251750 |
|  | EU251754 |
|  | EU251756 |
|  | EU251773 |
|  | EU251777 |
|  | EU251780 |
|  | EU251782 |
|  | EU251783 |
|  | EU251787 |
|  | EU251788 |
|  | EU251791 |
|  | EU251795 |
|  | EU251797 |
|  | EU251801 |
|  | EU251808 |
|  | EU251810 |
|  | EU251815 |
|  | EU251821 |
|  | EU251822 |
|  | EU251823 |
|  | EU251825 |
|  | EU251829 |
|  | EU251832 |
|  | EU251833 |
|  | EU251834 |
|  | EU251835 |
|  | EU251836 |
|  | EU251843 |
|  | EU251852 |
|  | EU251856 |
|  | EU251857 |
|  | EU251858 |
|  | EU251862 |
|  | EU251865 |
|  | EU251866 |
|  | EU251873 |
|  | EU251874 |
|  | EU251876 |
|  | EU251877 |
|  | KP681799 |
|  | KP681767 |
|  | KP681811 |
|  | KP681813 |
|  | KP681815 |
|  | KP681816 |
|  | KP681772 |
|  | KP681714 |
|  | KP681718 |
|  | KP681726 |
|  | KP681731 |
|  | KP681735 |
|  | KP681780 |
|  | KP681778 |
|  | KP681759 |
|  | KP681748 |
|  | KP681793 |
|  | KP681795 |
|  | KP681796 |
|  | KP681810 |
|  | KP681704 |
|  | KP681710 |
|  | KP681712 |
|  | KP681717 |
|  | KP681775 |
|  | KP681765 |
|  | KP681776 |
|  | KP681727 |
|  | KP681728 |
|  | KP681729 |
|  | KP681744 |
|  | KP681746 |
|  | KP681750 |
|  | KP681749 |
|  | KP681752 |
|  | KP681753 |
|  | KP681787 |
|  | KP681760 |
|  | KX907346 |
|  | KX907378 |
|  | KX907414 |
|  | KX907423 |
|  | KX907431 |
|  | HM572374 |
|  | HM572366 |
|  | HM572392 |
|  | HM572376 |
|  | HM572372 |
|  | HM572386 |
|  | HM572395 |
|  | HM572379 |
|  | HM572373 |
|  | HM572382 |
|  | HM572375 |
|  | HM572393 |
|  | HM572371 |
|  | KM438241 |
|  | KM438242 |
|  | KM438247 |
|  | KM438248 |
|  | KM438250 |
|  | KM438240 |
|  | KM438252 |
|  | KM438254 |
|  | KM438255 |
|  | KM438257 |
|  | KM438258 |
|  | KM438261 |
|  | KM438262 |
|  | KM438263 |
|  | KM438264 |
|  | KM438265 |
|  | KM438269 |
|  | KM438270 |
|  | KM438273 |
|  | KM438277 |
|  | KM438281 |
|  | KM438286 |
|  | KM438289 |
|  | KM438292 |
|  | KM438293 |
|  | KM438294 |
|  | KM438305 |
|  | KY658694 |
|  | KY658695 |
|  | KY658696 |
|  | HM572368 |
|  | HM572394 |
|  | HM572390 |
|  | KM438309 |
|  | KM438316 |
|  | HM572369 |
|  | HM572389 |
|  | HM572367 |
|  | HM572365 |
|  | HM572381 |
|  | HM572388 |
|  | HM572370 |
|  | HM572391 |
|  | HM572387 |
|  | JQ730776 |
|  | KX775225 |
|  | KX775230 |
|  | KX775233 |
|  | KX775234 |
|  | KX775235 |
|  | KX775236 |
|  | KX775247 |
|  | KX775249 |
|  | KX775250 |
|  | KX775261 |
|  | KX775286 |
|  | KX775287 |
|  | KX775291 |
|  | KX775226 |
|  | KX775255 |
|  | KX775257 |
|  | KX775258 |
|  | KX775260 |
|  | KX775262 |
|  | KX775265 |
|  | KX775270 |
|  | KX775277 |
|  | KX775280 |
|  | KX775283 |
|  | KX775292 |
|  | KX775294 |
|  | KX775300 |
|  | KX775301 |
|  | KX775302 |
|  | MH366803 |
|  | MG764290 |
|  | MH366821 |
|  | MH366824 |
|  | MH366826 |
|  | MH366827 |
|  | MH366836 |
|  | MH366837 |
|  | MH366838 |
|  | MH366842 |
|  | MH366843 |
|  | MH366897 |
|  | MH366807 |
|  | MH366901 |
|  | MH444904 |
|  | MH366906 |
|  | MH366907 |
|  | MH444906 |
|  | MH366913 |
|  | MH366914 |
|  | MH366915 |
|  | MH366916 |
|  | MH366917 |
|  | MH366919 |
|  | MH366922 |
|  | MH366923 |
|  | MH366928 |
|  | MH366929 |
|  | MH366934 |
|  | MH366938 |
|  | MH366939 |
|  | MH366940 |
|  | MH366944 |
|  | MH366945 |
|  | MH366950 |
|  | MH366952 |
|  | MH366957 |
|  | MH366958 |
|  | MH366960 |
|  | MH366961 |
|  | MH366963 |
|  | MH444909 |
|  | MH366968 |
|  | MH366969 |
|  | MH366975 |
|  | MH366976 |
|  | MH366978 |
|  | MH366979 |
|  | MH366981 |
|  | MH366982 |
|  | MH444912 |
|  | MH366988 |
|  | MH366991 |
|  | MH366995 |
|  | MH366997 |
|  | MH366998 |
|  | MH366999 |
|  | MH367001 |
|  | MH367002 |
|  | MH367008 |
|  | MH367009 |
|  | MH367010 |
|  | MH367011 |
|  | MH366810 |
|  | MH366812 |
|  | MG764294 |
|  | MH366815 |
|  | MH366819 |
|  | MH366846 |
|  | MH366847 |
|  | MH366849 |
|  | MH366850 |
|  | MH366853 |
|  | MH366857 |
|  | MH366862 |
|  | MH366865 |
|  | MH366866 |
|  | MH366867 |
|  | MH366870 |
|  | MH366872 |
|  | MH366873 |
|  | MH366874 |
|  | MH366875 |
|  | MH366876 |
|  | MH366880 |
|  | MH366882 |
|  | MH366886 |
|  | MH366887 |
|  | MH366891 |
|  | MH366892 |
|  | MH366893 |
|  | MH366894 |
|  | MH366895 |
|  | MH444901 |
|  | MK946461 |
|  | MK946465 |
|  | MK946466 |
|  | MK946468 |
|  | MK946469 |
|  | MK946470 |
|  | MK946471 |
|  | MK946474 |
|  | MK946478 |
|  | MK946481 |
|  | MK946484 |
|  | MK946492 |
|  | MK946493 |
|  | MK946494 |
|  | MK946498 |
|  | MK946501 |
|  | MK946502 |
|  | MK946506 |
|  | MK946513 |
|  | MK946517 |
|  | MK946523 |
|  | MK946530 |
|  | MK946532 |
|  | MK946534 |
|  | MK946535 |
|  | MK946536 |
|  | MK946537 |
|  | MK946543 |
|  | MK946544 |
|  | MK946546 |
|  | MK946547 |
|  | MK946549 |
|  | MK946554 |
|  | MK946556 |
|  | MK946558 |
|  | MK946559 |
|  | MK946562 |
|  | MK946565 |
|  | MK946566 |
|  | MK946570 |
|  | MK946571 |
|  | MK946572 |
|  | MK946575 |
|  | MK946576 |
|  | MK946580 |
|  | MK946582 |
|  | MK946586 |
|  | MK946587 |
|  | MK946589 |
|  | MK946590 |
|  | MK946592 |
|  | MK946593 |
|  | MK946594 |
|  | MK946601 |
|  | MK946603 |
|  | MK946604 |
|  | MK946605 |
|  | MK946607 |
|  | MK946608 |
|  | MK946612 |
|  | MK946613 |
|  | MK946615 |
|  | MK946618 |
|  | MK946619 |
|  | MK946620 |
|  | EF611991 |
|  | EF611994 |
|  | EF611995 |
|  | EF611996 |
|  | EF611997 |
|  | EF611999 |
|  | EF612001 |
|  | EF612002 |
|  | EF612003 |
|  | EF612004 |
|  | EF612007 |
|  | EF612010 |
|  | AY955692 |
|  | AY955693 |
|  | AY955694 |
|  | AY955695 |
|  | AF410204 |
|  | AY955696 |
|  | AF410205 |
|  | EF612183 |
|  | AY955700 |
|  | AY955701 |
|  | AY955704 |
|  | AY955705 |
|  | AY955707 |
|  | AY955708 |
|  | AY955709 |
|  | AY955710 |
|  | AY955711 |
|  | AF410210 |
|  | AF410211 |
|  | AF410213 |
|  | AY955712 |
|  | AF410215 |
|  | AY955713 |
|  | AY955714 |
|  | AY955716 |
|  | AY955717 |
|  | AY955718 |
|  | AY955720 |
|  | EF612184 |
|  | AF410220 |
|  | AF410221 |
|  | AY955725 |
|  | AF410222 |
|  | AF410223 |
|  | AY955727 |
|  | AY955728 |
|  | AY955729 |
|  | EF612185 |
|  | AF410224 |
|  | AY955732 |
|  | AY955733 |
|  | AY955735 |
|  | AY955736 |
|  | AF410227 |
|  | AY955737 |
|  | AF410228 |
|  | AF410230 |
|  | AF410231 |
|  | AY955740 |
|  | AF410232 |
|  | AF410233 |
|  | AY955743 |
|  | AY955744 |
|  | EF612186 |
|  | AF410237 |
|  | AY955746 |
|  | EF612135 |
|  | AF410238 |
|  | AY955748 |
|  | AY955749 |
|  | AF410239 |
|  | AY955750 |
|  | AF410242 |
|  | AY955753 |
|  | AY955754 |
|  | AF410243 |
|  | AY955755 |
|  | AF410245 |
|  | AY955757 |
|  | AF410246 |
|  | AY955759 |
|  | AY955760 |
|  | AF410248 |
|  | AY955761 |
|  | AY955763 |
|  | AY955765 |
|  | AF410251 |
|  | AY955766 |
|  | AY955767 |
|  | AY955770 |
|  | AF410253 |
|  | AY955771 |
|  | AF410255 |
|  | EF612014 |
|  | EF612015 |
|  | EF612017 |
|  | EU715593 |
|  | EU380732 |
|  | EU380722 |
|  | EU380723 |
|  | EU380725 |
|  | EU380729 |
|  | EU380730 |
|  | EU380731 |
|  | EU380733 |
|  | EU380734 |
|  | EU380737 |
|  | EU380741 |
|  | EU380743 |
|  | EU380744 |
|  | EU380746 |
|  | EU380749 |
|  | EU380750 |
|  | EU380751 |
|  | EF612019 |
|  | EF612020 |
|  | EF612021 |
|  | EF612022 |
|  | EU380755 |
|  | EU380756 |
|  | EU380757 |
|  | EU380759 |
|  | EU380760 |
|  | EF612024 |
|  | EF612025 |
|  | EF612026 |
|  | EU380762 |
|  | EF612027 |
|  | EF612028 |
|  | EF612030 |
|  | EF612033 |
|  | EU380764 |
|  | EF612036 |
|  | EF612039 |
|  | EU380767 |
|  | EF612042 |
|  | EF612043 |
|  | EF612045 |
|  | EF612048 |
|  | EF612049 |
|  | EF612052 |
|  | EF612056 |
|  | EF612058 |
|  | EF612060 |
|  | EF612062 |
|  | EF612065 |
|  | EF612066 |
|  | EF612067 |
|  | EF612068 |
|  | EF612069 |
|  | EF612070 |
|  | EF612072 |
|  | EF612073 |
|  | EF612075 |
|  | EF612077 |
|  | EF612080 |
|  | EF612082 |
|  | EF612085 |
|  | EF612086 |
|  | EF612089 |
|  | EF612090 |
|  | EF612092 |
|  | EF612094 |
|  | EF612095 |
|  | EF612096 |
|  | EF612097 |
|  | EF612098 |
|  | EF612099 |
|  | EF612100 |
|  | EF612102 |
|  | EF612104 |
|  | EF612107 |
|  | EF612108 |
|  | EF612109 |
|  | EF612111 |
|  | EF612113 |
|  | EF612114 |
|  | EF612115 |
|  | EF612117 |
|  | EF612120 |
|  | EF612121 |
|  | EF612122 |
|  | EF612123 |
|  | EF612125 |
|  | EF612127 |
|  | EF612129 |
|  | EF612132 |
|  | EF612134 |
|  | EF612140 |
|  | EF612141 |
|  | EF612147 |
|  | EF612149 |
|  | EF612150 |
|  | EF612151 |
|  | EF612153 |
|  | EF612155 |
|  | EF612156 |
|  | EF612158 |
|  | EF612159 |
|  | EF612160 |
|  | EF612163 |
|  | EF612164 |
|  | EF612165 |
|  | EF612166 |
|  | EF612168 |
|  | EF612173 |
|  | EF612174 |
|  | EF612175 |
|  | EF612176 |
|  | EF612178 |
|  | EF612179 |
|  | EF612180 |
|  | EF612181 |
|  | AY444180 |
|  | AY444181 |
|  | AY444182 |
|  | AY444183 |
|  | AY444184 |
|  | AY444185 |
|  | AY444186 |
|  | AY444187 |
|  | MH925337 |
|  | AB098331 |
|  | AB485632 |
|  | M62320 |
|  | AF447846 |
|  | AF107368 |
|  | AF107369 |
|  | AF107370 |
|  | AF107371 |
|  | AF107372 |
|  | AF447858 |
|  | AY713407 |
|  | U51190 |
|  | AB253429 |
|  | AB098333 |
|  | AY129510 |
|  | AY129511 |
|  | AY129512 |
|  | AY129513 |
|  | AY129514 |
|  | AY129515 |
|  | AY129516 |
|  | AY129517 |
|  | AY129518 |
|  | AY129519 |
|  | AY129520 |
|  | AY129521 |
|  | AY129522 |
|  | AY129523 |
|  | AY129524 |
|  | AY129525 |
|  | AY129526 |
|  | AY129527 |
|  | AY129528 |
|  | AY129529 |
|  | AY435223 |
|  | AY435241 |
|  | AY435251 |
|  | AF388070 |
|  | AY428678 |
|  | AY435390 |
|  | DQ230336 |
|  | DQ230337 |
|  | DQ230338 |
|  | DQ230339 |
|  | DQ230340 |
|  | AF388137 |
|  | AY435250 |
|  | AF388071 |
|  | AY428682 |
|  | AF388138 |
|  | AY428677 |
|  | AY435391 |
|  | DQ230341 |
|  | DQ230342 |
|  | DQ230343 |
|  | AY435247 |
|  | AY435261 |
|  | AY435255 |
|  | AY435256 |
|  | AY428671 |
|  | AY435225 |
|  | AY435248 |
|  | AY435262 |
|  | AY435260 |
|  | AY435252 |
|  | AY435264 |
|  | AY435226 |
|  | AY435231 |
|  | AY435229 |
|  | AY435224 |
|  | AF388075 |
|  | AY428683 |
|  | AY435280 |
|  | AY435269 |
|  | AY435265 |
|  | AY435274 |
|  | AF388076 |
|  | AY435267 |
|  | AY435290 |
|  | AF388078 |
|  | AY428685 |
|  | AY435283 |
|  | AY435272 |
|  | AF388067 |
|  | AF388079 |
|  | AF388080 |
|  | AF388142 |
|  | AY435289 |
|  | AY435273 |
|  | AY428672 |
|  | AY435228 |
|  | AY435279 |
|  | AF388081 |
|  | AY428686 |
|  | AY435286 |
|  | AY435227 |
|  | AY435271 |
|  | AY428687 |
|  | AY435281 |
|  | AY435292 |
|  | AF388089 |
|  | AF388090 |
|  | AY435351 |
|  | AY435282 |
|  | AY428675 |
|  | AY435234 |
|  | AY435288 |
|  | AY435296 |
|  | AF388144 |
|  | AF388145 |
|  | AY428697 |
|  | AF388146 |
|  | AY428696 |
|  | AF388091 |
|  | AF388148 |
|  | AY428691 |
|  | AY435393 |
|  | DQ230344 |
|  | AY435300 |
|  | AY435303 |
|  | AY435311 |
|  | AY435232 |
|  | AY435312 |
|  | AY435315 |
|  | AF388094 |
|  | AY435302 |
|  | AF388150 |
|  | AY435240 |
|  | AY435336 |
|  | AY435314 |
|  | AF388096 |
|  | AY428695 |
|  | AY435304 |
|  | AF388151 |
|  | AY428703 |
|  | AY435319 |
|  | AF388097 |
|  | AY428702 |
|  | AY435394 |
|  | DQ230330 |
|  | DQ230333 |
|  | DQ230345 |
|  | DQ230346 |
|  | AF388098 |
|  | AY428699 |
|  | AF388100 |
|  | AY435318 |
|  | AY435320 |
|  | AF388103 |
|  | AF388104 |
|  | AY428701 |
|  | AY435317 |
|  | AF388106 |
|  | AF388107 |
|  | AF388068 |
|  | AY435310 |
|  | AF388108 |
|  | AY428706 |
|  | AF388109 |
|  | AY435324 |
|  | AF388111 |
|  | AY435327 |
|  | AY435329 |
|  | AY428705 |
|  | DQ230347 |
|  | DQ230348 |
|  | DQ230349 |
|  | DQ230350 |
|  | DQ230351 |
|  | AY435339 |
|  | AY435334 |
|  | AY435335 |
|  | AY435333 |
|  | AF388113 |
|  | AY428712 |
|  | AY435396 |
|  | DQ230331 |
|  | DQ230334 |
|  | DQ230352 |
|  | DQ230353 |
|  | DQ230354 |
|  | AY435238 |
|  | AY435340 |
|  | AY435331 |
|  | AF388155 |
|  | AY435332 |
|  | AF388114 |
|  | AF388115 |
|  | AY428710 |
|  | AF388116 |
|  | AY435343 |
|  | AY435363 |
|  | AY435347 |
|  | AF388118 |
|  | AY435346 |
|  | AF388120 |
|  | AY428718 |
|  | AY435358 |
|  | AY435355 |
|  | AY435356 |
|  | AY435353 |
|  | AY435361 |
|  | AY435364 |
|  | AF388159 |
|  | AY428721 |
|  | AY435397 |
|  | DQ230335 |
|  | DQ230356 |
|  | DQ230357 |
|  | DQ230358 |
|  | AF388127 |
|  | AY435365 |
|  | AY428717 |
|  | AY435354 |
|  | AF388129 |
|  | AY428723 |
|  | AY435374 |
|  | AY435376 |
|  | AY435366 |
|  | AY435368 |
|  | AY435371 |
|  | AY435379 |
|  | AF388130 |
|  | AY428726 |
|  | AY425357 |
|  | AY428729 |
|  | AY435398 |
|  | DQ230359 |
|  | DQ230360 |
|  | DQ230361 |
|  | AY435373 |
|  | AF388164 |
|  | AY428727 |
|  | AY435399 |
|  | DQ230362 |
|  | DQ230363 |
|  | DQ230364 |
|  | AY435236 |
|  | AY428728 |
|  | AY435381 |
|  | AY435378 |
|  | AY435384 |
|  | AF388166 |
|  | AY428732 |
|  | AY435380 |
|  | AF388133 |
|  | AY428730 |
|  | AY435385 |
|  | AY435386 |
|  | AY435245 |
|  | FJ389058 |
|  | FJ389066 |
|  | FJ389067 |
|  | FJ389068 |
|  | FJ389073 |
|  | FJ389074 |
|  | FJ389076 |
|  | FJ389079 |
|  | FJ389080 |
|  | FJ389083 |
|  | FJ389085 |
|  | FJ389099 |
|  | FJ389100 |
|  | FJ389103 |
|  | FJ389108 |
|  | FJ389110 |
|  | FJ389111 |
|  | FJ389114 |
|  | FJ389115 |
|  | FJ389118 |
|  | FJ389128 |
|  | FJ389146 |
|  | FJ389147 |
|  | FJ389153 |
|  | AF484507 |
|  | AF484508 |
|  | AF484509 |
|  | AF484512 |
|  | AY425353 |
|  | AY425356 |
|  | AF484478 |
|  | AF484493 |
|  | DQ079853 |
|  | DQ079848 |
|  | DQ079847 |
|  | DQ079846 |
|  | AY803467 |
|  | AY803462 |
|  | DQ079844 |
|  | DQ079841 |
|  | AY803460 |
|  | KJ906626 |
|  | KJ906627 |
|  | KJ906631 |
|  | KJ906632 |
|  | KJ906633 |
|  | KJ906634 |
|  | KJ906635 |
|  | KJ906636 |
|  | KJ906637 |
|  | KJ906639 |
|  | KJ906642 |
|  | KJ906643 |
|  | KJ906645 |
|  | KJ906646 |
|  | KJ906647 |
|  | KJ906649 |
|  | KJ906650 |
|  | KJ906654 |
|  | KJ906656 |
|  | KJ906657 |
|  | KJ906658 |
|  | KJ906659 |
|  | KJ906660 |
|  | KJ906665 |
|  | KJ906666 |
|  | KJ906667 |
|  | KJ906671 |
|  | KJ906672 |
|  | KJ906673 |
|  | KJ906674 |
|  | KJ906676 |
|  | KJ906681 |
|  | KJ906688 |
|  | KJ906691 |
|  | KJ906692 |
|  | KJ906695 |
|  | KJ906696 |
|  | KJ906698 |
|  | KJ906700 |
|  | KJ906701 |
|  | DQ079840 |
|  | DQ079837 |
|  | DQ079835 |
|  | DQ079834 |
|  | DQ079833 |
|  | DQ079832 |
|  | DQ079831 |
|  | DQ079830 |
|  | DQ079827 |
|  | DQ079826 |
|  | AY803485 |
|  | DQ079825 |
|  | DQ079824 |
|  | DQ079822 |
|  | AY803464 |
|  | AY803471 |
|  | AY803470 |
|  | AY803472 |
|  | AY803474 |
|  | AY803473 |
|  | GU059285 |
|  | GU059288 |
|  | GU059289 |
|  | GU059301 |
|  | AY803481 |
|  | GQ409546 |
|  | GU059292 |
|  | GU059293 |
|  | GU059296 |
|  | GU059297 |
|  | GU059299 |
|  | GU059300 |
|  | GU059311 |
|  | GU059312 |
|  | GU059313 |
|  | GU059314 |
|  | GU059317 |
|  | GU059318 |
|  | GU059319 |
|  | GU059320 |
|  | GU059321 |
|  | GU059322 |
|  | GU059323 |
|  | GU059324 |
|  | GU059327 |
|  | GU059328 |
|  | GU059331 |
|  | GU059332 |
|  | GU059335 |
|  | GU059336 |
|  | GU059337 |
|  | GU059338 |
|  | HM037836 |
|  | HM037837 |
|  | HM037838 |
|  | HM037839 |
|  | HM037810 |
|  | HM037811 |
|  | HM037840 |
|  | HM037842 |
|  | HM037843 |
|  | HM037813 |
|  | HM037846 |
|  | HM037847 |
|  | HM037849 |
|  | HM037852 |
|  | HM037814 |
|  | HM037815 |
|  | HM037816 |
|  | HM037853 |
|  | HM037817 |
|  | HM037855 |
|  | HM037857 |
|  | HM037819 |
|  | HM037820 |
|  | HM037821 |
|  | HM037822 |
|  | HM037860 |
|  | HM037862 |
|  | HM037863 |
|  | HM037824 |
|  | HM037865 |
|  | HM037866 |
|  | HM037867 |
|  | HM037870 |
|  | HM037873 |
|  | HM037874 |
|  | HM037876 |
|  | HM037879 |
|  | HM037826 |
|  | HM037827 |
|  | HM037828 |
|  | HM037829 |
|  | HM037831 |
|  | GQ409549 |
|  | GQ409550 |
|  | GQ409551 |
|  | GQ409552 |
|  | GQ409556 |
|  | GQ409560 |
|  | GQ409562 |
|  | GQ409565 |
|  | GQ409567 |
|  | GQ409568 |
|  | GQ409569 |
|  | GQ409570 |
|  | GQ409572 |
|  | GQ409574 |
|  | GQ409575 |
|  | GQ409576 |
|  | GQ409577 |
|  | GQ409580 |
|  | GQ409581 |
|  | GQ409582 |
|  | GQ409585 |
|  | GQ409588 |
|  | GQ409591 |
|  | HM037884 |
|  | HM037885 |
|  | HM037886 |
|  | HM037888 |
|  | HM037889 |
|  | HM037891 |
|  | HM037892 |
|  | HM037893 |
|  | GQ409598 |
|  | GQ409600 |
|  | GQ409602 |
|  | GQ409603 |
|  | GQ409605 |
|  | GQ409609 |
|  | GQ409610 |
|  | GQ409611 |
|  | GQ409612 |
|  | GQ409614 |
|  | GQ409635 |
|  | GQ409615 |
|  | GQ409617 |
|  | GQ409619 |
|  | GQ409620 |
|  | GQ409623 |
|  | GQ409625 |
|  | GQ409628 |
|  | GQ409629 |
|  | JN652153 |
|  | JN652196 |
|  | KJ906707 |
|  | KJ906712 |
|  | KJ906713 |
|  | KJ906714 |
|  | KJ906715 |
|  | KJ906716 |
|  | KJ906717 |
|  | KJ906721 |
|  | KJ906724 |
|  | EF186204 |
|  | KJ906728 |
|  | EF186206 |
|  | KJ906729 |
|  | KJ906730 |
|  | KJ906731 |
|  | EF186207 |
|  | KJ906736 |
|  | KJ906737 |
|  | KJ906738 |
|  | EF186211 |
|  | KJ906742 |
|  | KJ906749 |
|  | KJ906750 |
|  | KJ906753 |
|  | KJ906754 |
|  | EF186218 |
|  | KJ906759 |
|  | KJ906761 |
|  | KJ906764 |
|  | KJ906767 |
|  | EF186221 |
|  | EF186223 |
|  | EF186225 |
|  | EF186226 |
|  | EF186134 |
|  | EF186137 |
|  | EF186138 |
|  | EF186140 |
|  | EF186142 |
|  | EF186145 |
|  | EF186146 |
|  | EF186150 |
|  | JQ266062 |
|  | JQ266074 |
|  | JQ266034 |
|  | KC018694 |
|  | HM037896 |
|  | HM037897 |
|  | HM037898 |
|  | HM037900 |
|  | HM037901 |
|  | KC019092 |
|  | JN652139 |
|  | EU306745 |
|  | EU306746 |
|  | EU306747 |
|  | EU306751 |
|  | EU306752 |
|  | EU306753 |
|  | EU306756 |
|  | EU306755 |
|  | EU306759 |
|  | EU306761 |
|  | EU306763 |
|  | EU306764 |
|  | EU306768 |
|  | EU306769 |
|  | EU306770 |
|  | EU306772 |
|  | EU306773 |
|  | EU306776 |
|  | EU306777 |
|  | EU306778 |
|  | EU306779 |
|  | EU306784 |
|  | EU306786 |
|  | EU306794 |
|  | JN652167 |
|  | JN652166 |
|  | JN652168 |
|  | KJ906774 |
|  | KJ906776 |
|  | KJ906777 |
|  | KJ906778 |
|  | KJ906780 |
|  | KJ906781 |
|  | KJ906782 |
|  | KJ906783 |
|  | KJ906784 |
|  | KJ906785 |
|  | KJ906786 |
|  | KJ906787 |
|  | KJ906788 |
|  | KJ906790 |
|  | KJ906791 |
|  | KJ906796 |
|  | KJ906797 |
|  | KJ906798 |
|  | KJ906799 |
|  | KJ906800 |
|  | KJ906802 |
|  | KJ906803 |
|  | KJ906808 |
|  | KJ906810 |
|  | KJ906815 |
|  | KJ906818 |
|  | KJ906820 |
|  | KJ906828 |
|  | KJ906829 |
|  | KJ906831 |
|  | KJ906832 |
|  | KJ906834 |
|  | KJ906837 |
|  | KJ906839 |
|  | KJ906840 |
|  | KJ906841 |
|  | KJ906844 |
|  | KJ906847 |
|  | KJ906849 |
|  | KJ906850 |
|  | KJ906853 |
|  | KJ906857 |
|  | KJ906860 |
|  | KJ906862 |
|  | KJ906867 |
|  | KJ906869 |
|  | KJ906870 |
|  | KJ906871 |
|  | KJ906873 |
|  | KJ906875 |
|  | KJ906876 |
|  | KJ906878 |
|  | KJ906884 |
|  | JQ266088 |
|  | JQ266057 |
|  | JX202785 |
|  | JX202786 |
|  | JX202787 |
|  | JX202788 |
|  | JX202789 |
|  | JX202790 |
|  | JX202791 |
|  | JX202792 |
|  | JX202793 |
|  | JX202794 |
|  | JX202795 |
|  | JX202868 |
|  | JX202869 |
|  | JX202870 |
|  | JX202871 |
|  | JX202872 |
|  | JX202873 |
|  | JX202874 |
|  | JX202875 |
|  | JX202876 |
|  | JX202877 |
|  | JX202878 |
|  | JX202879 |
|  | KC018589 |
|  | KC018802 |
|  | KC018863 |
|  | KC018944 |
|  | KC018975 |
|  | KC018588 |
|  | KC019015 |
|  | KC019039 |
|  | KC019053 |
|  | JX202934 |
|  | JX202935 |
|  | JX202936 |
|  | JX202937 |
|  | JX202938 |
|  | JX202939 |
|  | JX202940 |
|  | JX202941 |
|  | JX202942 |
|  | JX202943 |
|  | JX202944 |
|  | JX203151 |
|  | JX203152 |
|  | JX203153 |
|  | JX203154 |
|  | JX203155 |
|  | JX203156 |
|  | JX203157 |
|  | JX203158 |
|  | JX203159 |
|  | JX203160 |
|  | JX203161 |
|  | JX203162 |
|  | JX203163 |
|  | JX203164 |
|  | JX203165 |
|  | JX203166 |
|  | JX203167 |
|  | JQ480236 |
|  | JQ480237 |
|  | JQ480238 |
|  | JQ480240 |
|  | JQ480242 |
|  | JQ480244 |
|  | JQ480246 |
|  | JQ480247 |
|  | JQ480250 |
|  | JQ480251 |
|  | JQ480252 |
|  | JQ480255 |
|  | JQ480256 |
|  | JQ480259 |
|  | JQ480260 |
|  | JQ480261 |
|  | JQ480262 |
|  | JQ480266 |
|  | JQ480268 |
|  | JQ480270 |
|  | KF716500 |
|  | KC596070 |
|  | JN652186 |
|  | JN652203 |
|  | JN652204 |
|  | KJ906892 |
|  | KJ906893 |
|  | KJ906895 |
|  | KJ906896 |
|  | KJ906897 |
|  | KJ906899 |
|  | KJ906900 |
|  | KJ906903 |
|  | KJ906905 |
|  | KJ906907 |
|  | KJ906910 |
|  | KJ906911 |
|  | KJ906912 |
|  | KJ906913 |
|  | KJ906914 |
|  | KJ906915 |
|  | KJ906916 |
|  | KJ906919 |
|  | KJ906921 |
|  | KJ906922 |
|  | KJ906923 |
|  | KJ906924 |
|  | KJ906925 |
|  | KJ906927 |
|  | KJ906928 |
|  | KJ906931 |
|  | KJ906932 |
|  | KJ906933 |
|  | KJ906934 |
|  | KJ906935 |
|  | KJ906936 |
|  | KJ906939 |
|  | KJ906940 |
|  | KJ906942 |
|  | KJ906944 |
|  | KJ906946 |
|  | KJ906948 |
|  | KJ906949 |
|  | KJ906950 |
|  | KJ906952 |
|  | KJ906954 |
|  | KJ906956 |
|  | KJ906960 |
|  | KJ906961 |
|  | KJ906962 |
|  | KJ906963 |
|  | KJ906966 |
|  | KJ906969 |
|  | KJ906970 |
|  | KJ906971 |
|  | KJ906972 |
|  | KJ906975 |
|  | KJ906976 |
|  | KJ906978 |
|  | KJ906979 |
|  | KJ906982 |
|  | KJ906987 |
|  | KJ906990 |
|  | KJ906992 |
|  | KJ906993 |
|  | KJ906994 |
|  | KJ906997 |
|  | KJ906998 |
|  | KJ906999 |
|  | KJ907001 |
|  | KJ907003 |
|  | KJ907006 |
|  | KJ907008 |
|  | KJ907010 |
|  | KJ907011 |
|  | KJ907021 |
|  | KJ907022 |
|  | KJ907023 |
|  | KJ907024 |
|  | KJ907028 |
|  | KJ907030 |
|  | KJ907034 |
|  | KJ907035 |
|  | KJ907037 |
|  | KJ907040 |
|  | KJ907048 |
|  | KJ907049 |
|  | JX236669 |
|  | JX236671 |
|  | JX236676 |
|  | JQ266055 |
|  | JQ266044 |
|  | JQ266090 |
|  | JQ266029 |
|  | JQ266039 |
|  | JQ266049 |
|  | JQ266084 |
|  | JQ266070 |
|  | JF800190 |
|  | KC018520 |
|  | KC018581 |
|  | KC018680 |
|  | KC018759 |
|  | JN652170 |
|  | JN652138 |
|  | JN652175 |
|  | HQ994918 |
|  | HQ994920 |
|  | HQ995313 |
|  | HQ995314 |
|  | HQ995315 |
|  | HQ995316 |
|  | HQ995317 |
|  | HQ994922 |
|  | HQ995318 |
|  | JN630954 |
|  | HQ994926 |
|  | HQ995099 |
|  | HQ995321 |
|  | HQ995326 |
|  | HQ995327 |
|  | HQ994930 |
|  | HQ994931 |
|  | JN630955 |
|  | HQ995329 |
|  | HQ994934 |
|  | HQ995331 |
|  | HQ995332 |
|  | HQ995333 |
|  | JN630959 |
|  | HQ994935 |
|  | HQ995334 |
|  | HQ995336 |
|  | HQ995337 |
|  | HQ994938 |
|  | HQ995338 |
|  | HQ995339 |
|  | HQ995342 |
|  | HQ995343 |
|  | HQ995346 |
|  | HQ995347 |
|  | HQ994939 |
|  | HQ995348 |
|  | HQ995349 |
|  | HQ995351 |
|  | HQ995352 |
|  | HQ995353 |
|  | HQ995354 |
|  | JN630961 |
|  | HQ995357 |
|  | HQ995358 |
|  | HQ995360 |
|  | HQ995362 |
|  | HQ994942 |
|  | HQ994943 |
|  | HQ995102 |
|  | HQ995103 |
|  | HQ995104 |
|  | HQ994945 |
|  | HQ995105 |
|  | HQ994948 |
|  | HQ995106 |
|  | HQ994950 |
|  | HQ994951 |
|  | HQ994952 |
|  | HQ994953 |
|  | HQ995110 |
|  | HQ994955 |
|  | HQ994956 |
|  | HQ995113 |
|  | HQ994958 |
|  | HQ995114 |
|  | HQ995119 |
|  | HQ995120 |
|  | HQ995121 |
|  | HQ995122 |
|  | HQ995123 |
|  | HQ995124 |
|  | HQ995126 |
|  | HQ995127 |
|  | HQ994962 |
|  | HQ995131 |
|  | HQ995133 |
|  | HQ994964 |
|  | HQ995135 |
|  | HQ994965 |
|  | HQ995137 |
|  | HQ995138 |
|  | HQ995139 |
|  | HQ995141 |
|  | HQ994970 |
|  | HQ995143 |
|  | HQ994972 |
|  | HQ995145 |
|  | HQ995146 |
|  | HQ994974 |
|  | HQ995147 |
|  | HQ995148 |
|  | HQ994976 |
|  | HQ995150 |
|  | HQ995151 |
|  | HQ995152 |
|  | JN630964 |
|  | HQ995153 |
|  | HQ995154 |
|  | HQ995155 |
|  | HQ995156 |
|  | HQ995158 |
|  | HQ995159 |
|  | HQ995160 |
|  | HQ994980 |
|  | HQ995162 |
|  | HQ994981 |
|  | HQ994982 |
|  | HQ995163 |
|  | JN630965 |
|  | HQ994983 |
|  | HQ995164 |
|  | HQ995165 |
|  | JN630966 |
|  | HQ994985 |
|  | HQ994988 |
|  | HQ994990 |
|  | HQ994995 |
|  | HQ995172 |
|  | HQ994998 |
|  | HQ995173 |
|  | HQ995001 |
|  | HQ995003 |
|  | HQ995005 |
|  | HQ995006 |
|  | HQ995007 |
|  | JN630968 |
|  | HQ995008 |
|  | HQ995176 |
|  | HQ995009 |
|  | HQ995010 |
|  | HQ995012 |
|  | HQ995368 |
|  | HQ995369 |
|  | HQ995371 |
|  | HQ995376 |
|  | HQ995379 |
|  | HQ995380 |
|  | HQ995381 |
|  | HQ995382 |
|  | HQ995383 |
|  | HQ995385 |
|  | HQ995386 |
|  | HQ995387 |
|  | HQ995388 |
|  | HQ995390 |
|  | HQ995392 |
|  | HQ995393 |
|  | HQ995395 |
|  | HQ995397 |
|  | HQ995400 |
|  | HQ995404 |
|  | HQ995407 |
|  | HQ995408 |
|  | HQ995409 |
|  | HQ995412 |
|  | HQ995415 |
|  | HQ995416 |
|  | HQ995417 |
|  | HQ995420 |
|  | HQ995421 |
|  | HQ995430 |
|  | HQ995440 |
|  | HQ995014 |
|  | HQ995442 |
|  | HQ995015 |
|  | HQ995447 |
|  | HQ995448 |
|  | HQ995177 |
|  | HQ995178 |
|  | HQ995450 |
|  | HQ995181 |
|  | HQ995016 |
|  | HQ995452 |
|  | HQ995183 |
|  | HQ995184 |
|  | HQ995185 |
|  | HQ995453 |
|  | HQ995187 |
|  | HQ995188 |
|  | HQ995189 |
|  | HQ995190 |
|  | HQ995191 |
|  | HQ995195 |
|  | HQ995458 |
|  | HQ995459 |
|  | HQ995460 |
|  | HQ995197 |
|  | JN630977 |
|  | HQ995198 |
|  | HQ995019 |
|  | HQ995199 |
|  | JN630978 |
|  | HQ995020 |
|  | HQ995202 |
|  | HQ995204 |
|  | HQ995022 |
|  | HQ995024 |
|  | HQ995206 |
|  | HQ995029 |
|  | HQ995030 |
|  | HQ995032 |
|  | HQ995033 |
|  | JN630981 |
|  | HQ995034 |
|  | HQ995035 |
|  | HQ995209 |
|  | HQ995464 |
|  | HQ995210 |
|  | HQ995211 |
|  | HQ995466 |
|  | JN630982 |
|  | HQ995470 |
|  | HQ995471 |
|  | HQ995472 |
|  | HQ995473 |
|  | HQ995474 |
|  | HQ995476 |
|  | HQ995481 |
|  | HQ995482 |
|  | HQ995483 |
|  | HQ995485 |
|  | HQ995488 |
|  | HQ995215 |
|  | HQ995039 |
|  | HQ995043 |
|  | HQ995044 |
|  | HQ995217 |
|  | HQ995045 |
|  | HQ995047 |
|  | HQ995051 |
|  | HQ995052 |
|  | HQ995053 |
|  | HQ995054 |
|  | HQ995219 |
|  | HQ995056 |
|  | HQ995058 |
|  | HQ995061 |
|  | HQ995063 |
|  | HQ995066 |
|  | HQ995222 |
|  | HQ995070 |
|  | HQ995074 |
|  | HQ995076 |
|  | HQ995079 |
|  | HQ995080 |
|  | HQ995081 |
|  | HQ995083 |
|  | HQ995084 |
|  | HQ995086 |
|  | HQ995087 |
|  | HQ995088 |
|  | HQ995089 |
|  | HQ995095 |
|  | HQ995097 |
|  | HQ995223 |
|  | HQ995228 |
|  | HQ995230 |
|  | HQ995234 |
|  | HQ995308 |
|  | HQ995238 |
|  | HQ995239 |
|  | HQ995240 |
|  | HQ995241 |
|  | HQ995242 |
|  | HQ995243 |
|  | HQ995244 |
|  | HQ995245 |
|  | HQ995250 |
|  | HQ995253 |
|  | HQ995256 |
|  | HQ995258 |
|  | HQ995259 |
|  | HQ995262 |
|  | HQ995268 |
|  | HQ995270 |
|  | HQ995271 |
|  | HQ995273 |
|  | HQ995276 |
|  | HQ995278 |
|  | HQ995280 |
|  | HQ995282 |
|  | HQ995283 |
|  | HQ995290 |
|  | HQ995295 |
|  | HQ995296 |
|  | HQ995299 |
|  | HQ995300 |
|  | HQ995301 |
|  | HQ995305 |
|  | HQ995306 |
|  | HQ995307 |
|  | JN132241 |
|  | JN132242 |
|  | JN132243 |
|  | JN132247 |
|  | JN132250 |
|  | JN132251 |
|  | JN132252 |
|  | JN132253 |
|  | JN132279 |
|  | JN132280 |
|  | JN132281 |
|  | JN132283 |
|  | JN132285 |
|  | JN132287 |
|  | JN132288 |
|  | JN132289 |
|  | JN132291 |
|  | JN132311 |
|  | JN132313 |
|  | JN132315 |
|  | JN132319 |
|  | JN132323 |
|  | JN132324 |
|  | JN132329 |
|  | JN132331 |
|  | JN132335 |
|  | JN132338 |
|  | JN652202 |
|  | KJ907052 |
|  | KJ907055 |
|  | KJ907056 |
|  | KJ907058 |
|  | KJ907060 |
|  | KJ907064 |
|  | KJ907069 |
|  | KJ907070 |
|  | KJ907073 |
|  | KJ907075 |
|  | KJ907076 |
|  | KJ907077 |
|  | KJ907079 |
|  | KJ907083 |
|  | KJ907084 |
|  | KJ907085 |
|  | KJ907086 |
|  | KJ907088 |
|  | KJ907089 |
|  | KJ907091 |
|  | KJ907094 |
|  | KJ907097 |
|  | KJ907101 |
|  | KJ907103 |
|  | KJ907104 |
|  | KJ907105 |
|  | KJ907107 |
|  | KJ907109 |
|  | KJ907116 |
|  | KJ907119 |
|  | KJ907121 |
|  | KJ907122 |
|  | KJ907124 |
|  | KJ907126 |
|  | KJ907129 |
|  | KJ907130 |
|  | KJ907131 |
|  | KJ907138 |
|  | KJ907141 |
|  | KJ907144 |
|  | KJ907146 |
|  | KJ907147 |
|  | KJ907149 |
|  | KJ907151 |
|  | JQ266079 |
|  | JQ266063 |
|  | JQ266056 |
|  | JQ266076 |
|  | JQ266061 |
|  | JQ730782 |
|  | JQ730754 |
|  | JQ730755 |
|  | JQ730809 |
|  | JQ730785 |
|  | JQ730802 |
|  | JQ730770 |
|  | KC018560 |
|  | KC018570 |
|  | KC018586 |
|  | KC018606 |
|  | KC018612 |
|  | KC018654 |
|  | KC018655 |
|  | KC018676 |
|  | JF800194 |
|  | JF800215 |
|  | KC018701 |
|  | JF800196 |
|  | JF800217 |
|  | JF800198 |
|  | JF800199 |
|  | JF800218 |
|  | JF800225 |
|  | KC018769 |
|  | KC018775 |
|  | KC018790 |
|  | KC018851 |
|  | JF800202 |
|  | JN393298 |
|  | KC018878 |
|  | KC018890 |
|  | KC018955 |
|  | JN393299 |
|  | JN393300 |
|  | HQ702677 |
|  | HQ702676 |
|  | HQ702678 |
|  | HQ702680 |
|  | HQ702682 |
|  | HQ702675 |
|  | HQ702634 |
|  | HQ702649 |
|  | HQ702672 |
|  | HQ702670 |
|  | HQ702659 |
|  | HQ702657 |
|  | HQ702656 |
|  | HQ702652 |
|  | HQ702651 |
|  | HQ702633 |
|  | HQ702650 |
|  | HQ702618 |
|  | HQ702645 |
|  | HQ702617 |
|  | HQ702635 |
|  | HQ702640 |
|  | KF716478 |
|  | KP109490 |
|  | JN652210 |
|  | JQ625657 |
|  | JQ625654 |
|  | JQ625602 |
|  | JQ625628 |
|  | KJ907153 |
|  | KJ907155 |
|  | KJ907158 |
|  | KJ907161 |
|  | KJ907162 |
|  | KJ907165 |
|  | KJ907168 |
|  | KJ907172 |
|  | KJ907175 |
|  | KJ907176 |
|  | KJ907177 |
|  | KJ907178 |
|  | KJ907179 |
|  | KJ907182 |
|  | KJ907183 |
|  | KJ907185 |
|  | KJ907186 |
|  | KJ907189 |
|  | HQ702669 |
|  | HQ702664 |
|  | HQ702663 |
|  | HQ702661 |
|  | HQ702631 |
|  | HQ702630 |
|  | HQ702627 |
|  | HQ702626 |
|  | HQ702622 |
|  | HQ702620 |
|  | HQ702616 |
|  | HQ702628 |
|  | HQ702648 |
|  | JQ266065 |
|  | JQ266058 |
|  | JQ266059 |
|  | MG436509 |
|  | MG436573 |
|  | MG436590 |
|  | MG436616 |
|  | MG436617 |
|  | MG436618 |
|  | MG436621 |
|  | MG436623 |
|  | MG436625 |
|  | MG436626 |
|  | MG436627 |
|  | MG436630 |
|  | MG436631 |
|  | MG436635 |
|  | MG436636 |
|  | MG436637 |
|  | MG436639 |
|  | MG436640 |
|  | MG436641 |
|  | MG436642 |
|  | MG436644 |
|  | MG436645 |
|  | MG436646 |
|  | MG436649 |
|  | MG436651 |
|  | MG436653 |
|  | MG436659 |
|  | MG436662 |
|  | MG436663 |
|  | MG436665 |
|  | MG436669 |
|  | MG436670 |
|  | MG436671 |
|  | MG436673 |
|  | MG436675 |
|  | MG436676 |
|  | MG436677 |
|  | MG436678 |
|  | MG436682 |
|  | MG436684 |
|  | MG436685 |
|  | MG436686 |
|  | MG436687 |
|  | MG436690 |
|  | MG436691 |
|  | MG436692 |
|  | MG436695 |
|  | MG436711 |
|  | MG436721 |
|  | MG436724 |
|  | MG436725 |
|  | MG436728 |
|  | MG436729 |
|  | MG436731 |
|  | MG436732 |
|  | MG436737 |
|  | MG436739 |
|  | MG436740 |
|  | MG436743 |
|  | MG436745 |
|  | MG436748 |
|  | MG436751 |
|  | MG436752 |
|  | MG436755 |
|  | MG436759 |
|  | MG436760 |
|  | MG436766 |
|  | MG436767 |
|  | HQ702685 |
|  | JQ730756 |
|  | JQ730810 |
|  | JQ730798 |
|  | JX498988 |
|  | JX498989 |
|  | JQ730769 |
|  | KC018550 |
|  | JX498985 |
|  | JX498976 |
|  | JX498994 |
|  | JX498995 |
|  | JX498982 |
|  | KC018731 |
|  | KC018733 |
|  | KC018778 |
|  | KC018856 |
|  | JF800216 |
|  | JX498999 |
|  | JX499000 |
|  | JX499004 |
|  | JX499006 |
|  | JX499010 |
|  | KC018915 |
|  | KC018935 |
|  | KC018971 |
|  | JF800205 |
|  | JF800207 |
|  | JF800228 |
|  | JX499014 |
|  | JX499016 |
|  | KC019075 |
|  | JF800210 |
|  | JF800211 |
|  | JF800214 |
|  | MG549798 |
|  | HQ702638 |
|  | KX073740 |
|  | JX181984 |
|  | KX073718 |
|  | KX073712 |
|  | JX181989 |
|  | JX181990 |
|  | JX181991 |
|  | JX181992 |
|  | KX073725 |
|  | JX181995 |
|  | KX073742 |
|  | KX073743 |
|  | JX182001 |
|  | KX073719 |
|  | JX182006 |
|  | MF138738 |
|  | MF138741 |
|  | MF138742 |
|  | MF138744 |
|  | JX182009 |
|  | JX182011 |
|  | JQ625641 |
|  | JQ625647 |
|  | JQ625648 |
|  | JQ625644 |
|  | JQ625642 |
|  | JQ625643 |
|  | JQ625637 |
|  | JQ625636 |
|  | JQ625638 |
|  | JQ625635 |
|  | JQ625634 |
|  | JQ625627 |
|  | JQ625621 |
|  | JQ625620 |
|  | JQ625619 |
|  | JQ625614 |
|  | JQ625601 |
|  | KJ907191 |
|  | KJ907192 |
|  | KJ907193 |
|  | KJ907195 |
|  | KJ907196 |
|  | JX182012 |
|  | KX073714 |
|  | KX073715 |
|  | JX182015 |
|  | JX182017 |
|  | KX073734 |
|  | JX182018 |
|  | JX182020 |
|  | KX073723 |
|  | JX182023 |
|  | KX073724 |
|  | KX073735 |
|  | JX182024 |
|  | MF138736 |
|  | MF138734 |
|  | MF138735 |
|  | MF138685 |
|  | MF138686 |
|  | MF138687 |
|  | MF138688 |
|  | MF138689 |
|  | MF138690 |
|  | MF138691 |
|  | MF138694 |
|  | MF138695 |
|  | MF138696 |
|  | MF138697 |
|  | MF138698 |
|  | MF138701 |
|  | MF138704 |
|  | MF138705 |
|  | MF138716 |
|  | MF138708 |
|  | MF138709 |
|  | MF138710 |
|  | MF138711 |
|  | MF138712 |
|  | MF138714 |
|  | MF138719 |
|  | MF138720 |
|  | MF138721 |
|  | MF138717 |
|  | MF138727 |
|  | MF138728 |
|  | MF138729 |
|  | MF138730 |
|  | MF138731 |
|  | MF138718 |
|  | KX228810 |
|  | MG436697 |
|  | MG436701 |
|  | MG436712 |
|  | MG436714 |
|  | MG436715 |
|  | MG436717 |
|  | MG549467 |
|  | MG549468 |
|  | MG549470 |
|  | MG549473 |
|  | MG549476 |
|  | MG549477 |
|  | MG549482 |
|  | MG549488 |
|  | MG549489 |
|  | MG549562 |
|  | MG549593 |
|  | KC018717 |
|  | KC019055 |
|  | MG549635 |
|  | MG549636 |
|  | MG549637 |
|  | MG549665 |
|  | MG549667 |
|  | MG549668 |
|  | MG549683 |
|  | MG549696 |
|  | MG549701 |
|  | MG549704 |
|  | MG549717 |
|  | MG549718 |
|  | MG549719 |
|  | MG549720 |
|  | MG549737 |
|  | MG549739 |
|  | MG549741 |
|  | MG549745 |
|  | MG549780 |
|  | MG549783 |
|  | MG549791 |
|  | MG576331 |
|  | MG576332 |
|  | MG576337 |
|  | MG576338 |
|  | KF859745 |
|  | KF716486 |
|  | MG576340 |
|  | MG576342 |
|  | JQ625625 |
|  | JQ625624 |
|  | JQ625617 |
|  | JQ625612 |
|  | JQ625613 |
|  | JQ625611 |
|  | JQ625610 |
|  | JQ625599 |
|  | MG436399 |
|  | MG436403 |
|  | MG436404 |
|  | MG436407 |
|  | MG436410 |
|  | MG436412 |
|  | MG549462 |
|  | MG549479 |
|  | MG549480 |
|  | MG549483 |
|  | MG549487 |
|  | MG549494 |
|  | MG549495 |
|  | MG549498 |
|  | MG549503 |
|  | MG549505 |
|  | MG549506 |
|  | MG549507 |
|  | MG549510 |
|  | MG549511 |
|  | MG549512 |
|  | MG549517 |
|  | MG549518 |
|  | MG549520 |
|  | MG549522 |
|  | MG549523 |
|  | MG549524 |
|  | MG549527 |
|  | MG549528 |
|  | MG549529 |
|  | MG549530 |
|  | MG549533 |
|  | MG549534 |
|  | MG549541 |
|  | MG549563 |
|  | MG549564 |
|  | MG549572 |
|  | MG549591 |
|  | MG549597 |
|  | MG549600 |
|  | MG549626 |
|  | MG549638 |
|  | MG549639 |
|  | MG549674 |
|  | MG549684 |
|  | MG549685 |
|  | MG549702 |
|  | MG549703 |
|  | MG549705 |
|  | MG549715 |
|  | MG549729 |
|  | MG549730 |
|  | MG549732 |
|  | MG549733 |
|  | MG549734 |
|  | MG549738 |
|  | MG549752 |
|  | MG549755 |
|  | MG549756 |
|  | MG549757 |
|  | MG549762 |
|  | MG549772 |
|  | MG549775 |
|  | MG549776 |
|  | MG549779 |
|  | MG549784 |
|  | MG549788 |
|  | MG549789 |
|  | MG549790 |
|  | MG549792 |
|  | MG549794 |
|  | KX944681 |
|  | MG436417 |
|  | MG436418 |
|  | MG436419 |
|  | MG436420 |
|  | MG436421 |
|  | MG436422 |
|  | MG436424 |
|  | MG436425 |
|  | MG436426 |
|  | MG436427 |
|  | MG436428 |
|  | MG436429 |
|  | MG436430 |
|  | MG436432 |
|  | MG436434 |
|  | MG436435 |
|  | MG436436 |
|  | MG436438 |
|  | MG436440 |
|  | MG436441 |
|  | MG436443 |
|  | MG436447 |
|  | MG436450 |
|  | MG436452 |
|  | MG436454 |
|  | MG436455 |
|  | MG436456 |
|  | MG436460 |
|  | MG436463 |
|  | MG436464 |
|  | MG436465 |
|  | MG436467 |
|  | MG436470 |
|  | MG436474 |
|  | MG436475 |
|  | MG436477 |
|  | MG436478 |
|  | MG436480 |
|  | MG436481 |
|  | MG436483 |
|  | MG436484 |
|  | MG436485 |
|  | MG436486 |
|  | MG436491 |
|  | MG436492 |
|  | MG436493 |
|  | MG436497 |
|  | MG436498 |
|  | MG436499 |
|  | MG436500 |
|  | MG436501 |
|  | MG436502 |
|  | MG436503 |
|  | MG436504 |
|  | MG436505 |
|  | MG436507 |
|  | MG436508 |
|  | MG549526 |
|  | MG549538 |
|  | MG549539 |
|  | MG549540 |
|  | MG549544 |
|  | MG549568 |
|  | MG549573 |
|  | MG549574 |
|  | MG549575 |
|  | MG549578 |
|  | MG549584 |
|  | MG549589 |
|  | MG549603 |
|  | MG549604 |
|  | MG549607 |
|  | MG549613 |
|  | MG549617 |
|  | MG549618 |
|  | MG549619 |
|  | MG549620 |
|  | MG549621 |
|  | MG549622 |
|  | MG549624 |
|  | MG549625 |
|  | MG549629 |
|  | MG549630 |
|  | MG549631 |
|  | MG549632 |
|  | MG549648 |
|  | MG549649 |
|  | MG549653 |
|  | MG549658 |
|  | MG549659 |
|  | MG549687 |
|  | MG549699 |
|  | MG549740 |
|  | MG549747 |
|  | MG549768 |
|  | MG549769 |
|  | MG549770 |
|  | MG549773 |
|  | MG549777 |
|  | MG549785 |
|  | MG549786 |
|  | MG549787 |
|  | MG549797 |
|  | KX944666 |
|  | KX944676 |
|  | KX944682 |
|  | MG436510 |
|  | MG436516 |
|  | MG436520 |
|  | MG436524 |
|  | MG436528 |
|  | MG436529 |
|  | MG436530 |
|  | MG436531 |
|  | MG436535 |
|  | MG436536 |
|  | MG436539 |
|  | MG436540 |
|  | MG436541 |
|  | MG436544 |
|  | MG436545 |
|  | MG436547 |
|  | MG436550 |
|  | MG436552 |
|  | MG436557 |
|  | MG436561 |
|  | MG436575 |
|  | MG436577 |
|  | MG436579 |
|  | MG436580 |
|  | MG436583 |
|  | MG436587 |
|  | MG436591 |
|  | MG436592 |
|  | MG434786 |
|  | MG434789 |
|  | MG434790 |
|  | MG434791 |
|  | MG434792 |
|  | MG434793 |
|  | MG434795 |
|  | MG434796 |
|  | MG434798 |
|  | MG434799 |
|  | MG434803 |
|  | MG434805 |
|  | MG434807 |
|  | MG434808 |
|  | MG434809 |
|  | MG434811 |
|  | MG434812 |
|  | MG434818 |
|  | MG434819 |
|  | MG434820 |
|  | MG434821 |
|  | MG434822 |
|  | MG434823 |
|  | MG434824 |
|  | MG434825 |
|  | MG434826 |
|  | MG434829 |
|  | MG434830 |
|  | MG434831 |
|  | MG434832 |
|  | MG434834 |
|  | MG434835 |
|  | MG434837 |
|  | MG434838 |
|  | MG434842 |
|  | MG434846 |
|  | MG434847 |
|  | MG434848 |
|  | MG434849 |
|  | MG434855 |
|  | MG434858 |
|  | MG434865 |
|  | MG434866 |
|  | MG434868 |
|  | MG434873 |
|  | MG436600 |
|  | MG436603 |
|  | MG436605 |
|  | MG436607 |
|  | MH423510 |
|  | MH423512 |
|  | MH423514 |
|  | MH423524 |
|  | MH423527 |
|  | MG435358 |
|  | MG435361 |
|  | MG435362 |
|  | MG435363 |
|  | MG435364 |
|  | MG435366 |
|  | MG435368 |
|  | MG435369 |
|  | MG435370 |
|  | MG435371 |
|  | MG435373 |
|  | MG435379 |
|  | MG435382 |
|  | MG435383 |
|  | MG435385 |
|  | MG435386 |
|  | MG435388 |
|  | MG435390 |
|  | MG435392 |
|  | MG435395 |
|  | MG435397 |
|  | MG435399 |
|  | MG435400 |
|  | MG435401 |
|  | MG435403 |
|  | MG435404 |
|  | MG435405 |
|  | MG435406 |
|  | MG435408 |
|  | MG435409 |
|  | MG435411 |
|  | MG435415 |
|  | MG435417 |
|  | MG435419 |
|  | MG435420 |
|  | MG435421 |
|  | MG435422 |
|  | MG435424 |
|  | MG435425 |
|  | MG435431 |
|  | MG435436 |
|  | MG435438 |
|  | MG435439 |
|  | MG435441 |
|  | MG435442 |
|  | MG435443 |
|  | MG435444 |
|  | MG435445 |
|  | MG435447 |
|  | MG435449 |
|  | MG435451 |
|  | MG435452 |
|  | MG435453 |
|  | MG435454 |
|  | MG435456 |
|  | MG435468 |
|  | MG435472 |
|  | MG435477 |
|  | MG435479 |
|  | MG435483 |
|  | MG435484 |
|  | MG435486 |
|  | MG435488 |
|  | MG435489 |
|  | MG435490 |
|  | MG435494 |
|  | MG435498 |
|  | MG435499 |
|  | MG435500 |
|  | MG435502 |
|  | MG435503 |
|  | MG435504 |
|  | MG435505 |
|  | MG435506 |
|  | MG435514 |
|  | MG435515 |
|  | MG435517 |
|  | MG435520 |
|  | MG435521 |
|  | MG435523 |
|  | MG435524 |
|  | MG435525 |
|  | MG435527 |
|  | MG435529 |
|  | MG435532 |
|  | MG435533 |
|  | MG435537 |
|  | MG435540 |
|  | MG435541 |
|  | MG435545 |
|  | MG435546 |
|  | MG435548 |
|  | MG435551 |
|  | MG435552 |
|  | MG435556 |
|  | MG435557 |
|  | MG435558 |
|  | MG435559 |
|  | MG435563 |
|  | MG435564 |
|  | MG435566 |
|  | MG435567 |
|  | MG435572 |
|  | MG435574 |
|  | MG435576 |
|  | MG435578 |
|  | MG435579 |
|  | MG435583 |
|  | MG435588 |
|  | MG435589 |
|  | MG435590 |
|  | MG435591 |
|  | MG435593 |
|  | MG435594 |
|  | MG435595 |
|  | MG435597 |
|  | MG435603 |
|  | MG435611 |
|  | MG435621 |
|  | MG435624 |
|  | MG435625 |
|  | MG435628 |
|  | MG435630 |
|  | MG435631 |
|  | MG435632 |
|  | MG435635 |
| A6 | EU611273 |
|  | KF720940 |
|  | KF720941 |
|  | KF720942 |
|  | KF720944 |
|  | KF720945 |
|  | KF720946 |
|  | KF720947 |
|  | KF720948 |
|  | KF720949 |
|  | KF720950 |
|  | KF720951 |
|  | KF720952 |
|  | KF720953 |
|  | KF720954 |
|  | KF720956 |
|  | KF720957 |
|  | KF720958 |
|  | KF720960 |
|  | KF720961 |
|  | KF720962 |
|  | KM023164 |
|  | KM023165 |
|  | KM023166 |
|  | KM023167 |
|  | KM023168 |
|  | KM023170 |
|  | KM023171 |
|  | KF692159 |
|  | KF692160 |
|  | KF692161 |
|  | KF692162 |
|  | KF692163 |
|  | KF692164 |
|  | KF692166 |
|  | KF692167 |
|  | KF692168 |
|  | KF692171 |
|  | KF692201 |
|  | KF692202 |
|  | KF692203 |
|  | KF692207 |
|  | KU645857 |
|  | GQ399371 |
|  | GQ400101 |
|  | KC238024 |
|  | DQ328519 |
|  | DQ328520 |
|  | DQ328521 |
|  | DQ328522 |
|  | DQ328523 |
|  | DQ328524 |
|  | DQ328526 |
|  | DQ328527 |
|  | DQ328528 |
|  | DQ328529 |
|  | DQ328530 |
|  | DQ328531 |
|  | DQ328532 |
|  | DQ328533 |
|  | DQ328534 |
|  | DQ328535 |
|  | DQ328536 |
|  | DQ328537 |
|  | DQ328538 |
|  | DQ328539 |
|  | DQ328540 |
|  | DQ328541 |
|  | DQ328542 |
|  | DQ328543 |
|  | DQ328545 |
|  | DQ328546 |
|  | DQ328548 |
|  | DQ328549 |
|  | DQ328550 |
|  | DQ328551 |
|  | DQ328552 |
|  | DQ328553 |
|  | DQ328554 |
|  | DQ328555 |
|  | DQ328556 |
|  | DQ328557 |
|  | DQ328558 |
|  | EU248487 |
|  | JX301073 |
|  | KM190951 |
|  | KM190968 |
|  | KM190969 |
|  | KM190970 |
|  | KM190946 |
|  | KM190971 |
|  | KM190947 |
|  | KM190972 |
|  | KM190973 |
|  | KM190974 |
|  | KM190949 |
|  | KM190950 |
|  | KM190975 |
|  | KM190952 |
|  | KM190948 |
|  | KM190976 |
|  | FR686903 |
|  | AF193275 |
|  | EU345608 |
|  | EU345609 |
|  | EU345610 |
|  | EU345611 |
|  | EU345612 |
|  | EU345613 |
|  | MH494212 |
|  | HF679242 |
|  | HE657536 |
|  | HE657505 |
|  | HE657466 |
|  | HE657434 |
|  | HF679267 |
|  | HF679241 |
|  | HF679243 |
|  | HF679244 |
|  | HF679245 |
|  | HF679246 |
|  | HE657500 |
|  | HF679258 |
|  | HE657452 |
|  | HF679239 |
|  | HE657510 |
|  | HE657523 |
|  | HE657522 |
|  | HF679233 |
|  | HF679214 |
|  | HF679209 |
|  | HE657489 |
|  | HE657436 |
|  | HE657435 |
|  | HE657538 |
|  | HE657539 |
|  | HE657521 |
|  | HE657490 |
|  | HE657471 |
|  | HE657469 |
|  | HE657470 |
|  | HE657467 |
|  | HE657537 |
|  | HE657468 |
|  | HE657472 |
|  | HE657535 |
|  | HE657474 |
|  | HE657450 |
|  | HE657442 |
|  | HE657441 |
|  | HE657437 |
|  | HE657438 |
|  | HE657444 |
|  | HE657449 |
|  | HE657430 |
|  | HE657532 |
|  | HE657432 |
|  | HE657439 |
|  | HE657448 |
|  | HE657431 |
|  | HE657447 |
|  | HE657534 |
|  | HE657451 |
|  | HE657445 |
|  | HE657446 |
|  | HE657443 |
|  | HE657440 |
|  | HE657533 |
|  | HE657484 |
|  | HE657479 |
|  | HE657477 |
|  | HE657483 |
|  | HE657478 |
|  | HE657480 |
|  | HE657481 |
|  | HE657482 |
|  | HE657493 |
|  | HE657498 |
|  | HF679238 |
|  | HE657459 |
|  | HE657456 |
|  | HF679237 |
|  | HE657454 |
|  | HF679236 |
|  | HE657457 |
|  | HF679240 |
|  | HE657460 |
|  | HE657502 |
|  | HE657463 |
|  | HE657461 |
|  | HE657462 |
|  | HE577613 |
|  | HE577614 |
|  | HE577615 |
|  | HE577616 |
|  | HE577617 |
|  | HE577618 |
|  | HE657517 |
|  | HE657520 |
|  | MH494268 |
|  | HE657527 |
|  | HE657528 |
|  | HE657530 |
|  | HE657531 |
|  | HE657519 |
|  | HE657529 |
|  | HE657514 |
|  | HE657513 |
|  | HE657515 |
|  | HE657525 |
|  | HE657524 |
|  | HE657516 |
|  | HE657526 |
|  | HE657518 |
|  | HE657540 |
|  | HE657541 |
|  | HF679211 |
|  | HE657499 |
|  | MH494296 |
|  | HE657475 |
|  | HE657473 |
|  | HE657465 |
|  | HE657506 |
|  | HE657476 |
|  | HE657507 |
|  | HF679265 |
|  | MH494200 |
|  | HE657503 |
|  | HE657504 |
|  | HE657487 |
|  | HE657486 |
|  | MH494222 |
|  | MH494227 |
|  | HE657488 |
|  | HE657497 |
|  | HE657496 |
|  | HE657495 |
|  | HE657494 |
|  | HE657508 |
|  | HF679247 |
|  | MH494269 |
|  | MH494270 |
|  | HE657511 |
|  | HE657509 |
|  | HE657512 |
|  | HE657542 |
|  | HF679220 |
|  | HF679227 |
|  | HF679229 |
|  | HF679228 |
|  | HE657491 |
|  | HF679260 |
|  | HF679264 |
|  | HF679266 |
|  | HF679276 |
|  | HF679271 |
|  | HF679269 |
|  | HF679270 |
|  | HF679277 |
|  | HF679274 |
|  | HF679273 |
|  | LN600131 |
|  | LN600137 |
|  | LN600132 |
|  | HF679272 |
|  | HF679275 |
|  | HF679268 |
|  | HF679248 |
|  | HF679249 |
|  | HF679250 |
|  | HF679251 |
|  | HF679252 |
|  | HF679253 |
|  | HF679254 |
|  | HF679256 |
|  | HF679259 |
|  | MH494234 |
|  | MH494238 |
|  | MH494266 |
|  | MH494267 |
|  | HF679225 |
|  | HF679219 |
|  | HF679217 |
|  | HF679224 |
|  | HF679230 |
|  | HF679235 |
|  | HF679232 |
|  | HF679216 |
|  | HF679231 |
|  | MH494281 |
|  | HF679218 |
|  | HF679210 |
|  | HF679212 |
|  | HF679234 |
|  | HF679215 |
|  | HF679222 |
|  | HF679221 |
|  | HF586690 |
|  | HF679226 |
|  | HF679213 |
|  | HF679223 |
|  | MH494284 |
|  | MH494285 |
|  | MH494286 |
|  | MH494289 |
|  | MH494290 |
|  | MH494291 |
|  | MH494298 |
|  | MH494299 |
|  | MH494300 |
|  | HF679261 |
|  | HF679263 |
|  | LN600133 |
|  | LN600134 |
|  | LN600135 |
|  | LN600136 |
|  | LN600138 |
|  | MH494201 |
|  | MH494208 |
|  | MH494209 |
|  | KM190978 |
|  | KM190979 |
|  | MH494235 |
|  | MH494239 |
|  | MH494262 |
|  | MH494274 |
|  | MH494282 |
|  | MH494283 |
|  | KM190956 |
|  | KM190957 |
|  | KM190958 |
|  | KM190959 |
|  | KM190960 |
|  | KM190961 |
|  | KM190953 |
|  | KM190962 |
|  | KM190963 |
|  | KM190954 |
|  | KM190964 |
|  | KM190965 |
|  | KM190955 |
|  | KM190966 |
|  | KM190967 |
|  | MH494287 |
|  | MH494288 |
|  | MH494292 |
|  | MH494293 |
|  | MH494294 |
|  | MH494295 |
|  | MH494297 |
|  | KT983615 |
|  | MH494301 |
|  | LN600139 |
|  | KM190977 |
|  | KM190981 |
|  | KM190982 |
|  | MH494263 |
|  | MH494264 |
|  | MH494265 |
|  | MH494271 |
|  | MH494202 |
|  | MH494207 |
|  | MH494210 |
|  | MH494211 |
|  | MH494215 |
|  | MH494216 |
|  | MH494217 |
|  | MH494218 |
|  | MH494219 |
|  | MH494220 |
|  | MH494221 |
|  | MH494236 |
|  | MH494237 |
|  | MH494240 |
|  | MH494241 |
|  | MH494242 |
|  | MH494243 |
|  | MH494249 |
|  | MH494250 |
|  | MH494251 |
|  | MH494252 |
|  | MH494253 |
|  | MH494254 |
|  | MH494272 |
|  | MH494275 |
|  | MH494276 |
|  | MH494277 |
|  | MH494278 |
|  | MH494302 |
|  | MH494303 |
|  | MH494304 |
|  | MH494305 |
|  | MH494306 |
|  | MH494307 |
|  | MH494203 |
|  | MH494206 |
|  | MH494213 |
|  | MH494214 |
|  | MH494223 |
|  | MH494224 |
|  | MH494225 |
|  | MH494226 |
|  | MH494228 |
|  | MH494231 |
|  | MH494232 |
|  | MH494244 |
|  | MH494245 |
|  | MH494246 |
|  | MH494247 |
|  | MH494248 |
|  | MH494255 |
|  | MH494256 |
|  | MH494257 |
|  | MH494258 |
|  | MH494259 |
|  | MH494260 |
|  | MH494261 |
|  | MH494273 |
|  | MH494279 |
|  | MH494280 |
|  | MH494204 |
|  | MH494205 |
|  | JF769804 |
|  | JF769817 |
|  | MF684328 |
|  | KY713505 |
|  | FJ388892 |
|  | EU673388 |
|  | FJ388906 |
|  | EU673389 |
|  | EU673401 |
|  | EU673402 |
|  | EU673403 |
|  | FJ388950 |
|  | EU673405 |
|  | FJ388951 |
|  | JF683763 |
|  | JF683780 |
|  | JF683798 |
|  | KJ635951 |
|  | KJ635968 |
|  | KJ635974 |
|  | KJ635975 |
|  | KJ635996 |
|  | KJ636003 |
|  | KJ636004 |
|  | EU672582 |
|  | EU672639 |
|  | EU672612 |
|  | EU672692 |
|  | EU672703 |
|  | EU672709 |
|  | EU672691 |
|  | EU672704 |
|  | EU672689 |
|  | EU672717 |
|  | EU672741 |
|  | FJ842289 |
|  | GQ999124 |
|  | HQ025859 |
|  | FJ842314 |
|  | FJ842326 |
|  | FJ842329 |
|  | FJ842340 |
|  | GQ999121 |
|  | FJ842348 |
|  | FJ842355 |
|  | FJ842374 |
|  | GQ999059 |
|  | FJ842373 |
|  | GQ999081 |
|  | GQ999087 |
|  | GQ999089 |
|  | GQ999101 |
|  | GQ999098 |
|  | GQ999109 |
|  | GQ999123 |
|  | HQ025853 |
|  | GQ999116 |
|  | GQ999120 |
|  | HQ025900 |
|  | JN229132 |
|  | HQ025899 |
|  | HQ025910 |
|  | JN229053 |
|  | JN229195 |
|  | JN229155 |
|  | JN229113 |
|  | JN229069 |
|  | JN229081 |
|  | JN229098 |
|  | JN229196 |
|  | HQ025895 |
|  | EU672763 |
|  | DQ974921 |
|  | EU672610 |
|  | DQ975100 |
|  | EU672545 |
|  | EU672645 |
|  | FJ842299 |
|  | JN229077 |
|  | DQ974926 |
|  | DQ974995 |
|  | EU672737 |
|  | FJ842316 |
|  | GQ999094 |
|  | DQ974940 |
|  | DQ975146 |
|  | DQ975165 |
|  | DQ975143 |
|  | DQ975173 |
|  | DQ975141 |
|  | DQ975147 |
|  | EU672675 |
|  | DQ975159 |
|  | DQ974847 |
|  | DQ975074 |
|  | DQ975044 |
|  | FJ842298 |
|  | DQ975064 |
|  | DQ975120 |
|  | EU672634 |
|  | EU672672 |
|  | EU672734 |
|  | DQ975036 |
|  | DQ975039 |
|  | DQ975045 |
|  | DQ975105 |
|  | EU672534 |
|  | EU672781 |
|  | EU672546 |
|  | EU672551 |
|  | EU672574 |
|  | EU672566 |
|  | DQ975022 |
|  | AY694336 |
|  | AY694248 |
|  | AY694296 |
|  | AY694380 |
|  | AY694362 |
|  | JX299750 |
|  | JX299830 |
|  | JX299880 |
|  | JX300589 |
|  | JX300882 |
|  | JX301034 |
|  | JX301117 |
|  | JX301138 |
|  | JX299563 |
|  | JX299576 |
|  | JX300137 |
|  | JX300149 |
|  | JX300174 |
|  | JX300383 |
|  | JX300468 |
|  | JX300767 |
|  | JX300941 |
|  | JX301087 |
|  | KJ770147 |
|  | KX465919 |
|  | MH072851 |
|  | KJ770328 |
|  | KX465920 |
|  | KJ770546 |
|  | KX465921 |
|  | MH072716 |
|  | KY386741 |
|  | KJ771053 |
|  | KX466795 |
|  | KJ771121 |
|  | KX466831 |
|  | KJ771127 |
|  | KX466835 |
|  | MH072081 |
|  | MH073177 |
|  | MH073239 |
|  | KJ771421 |
|  | KX466832 |
|  | MH072089 |
|  | MH072711 |
|  | MH072888 |
|  | KJ771501 |
|  | KX467015 |
|  | MH072052 |
|  | MH072796 |
|  | KJ771708 |
|  | KX467117 |
|  | KJ771750 |
|  | KX467140 |
|  | MH073213 |
|  | MH073460 |
|  | MH074519 |
|  | MH072703 |
|  | KY386719 |
|  | MH074571 |
|  | MH074576 |
|  | MH074520 |
|  | MH074615 |
|  | MH073643 |
|  | MH074678 |
|  | AJ419502 |
|  | JX300972 |
|  | EF583222 |
|  | FJ481651 |
|  | MF403212 |
|  | MF403233 |
|  | MF403237 |
|  | MF403211 |
|  | GQ240969 |
|  | KC340417 |
|  | MF403207 |
|  | JF929157 |
|  | GQ241002 |
|  | GQ241115 |
|  | KC340407 |
|  | KC340436 |
|  | MF403225 |
|  | KC340416 |
|  | KC340475 |
|  | KC340486 |
|  | JQ351963 |
|  | KC340495 |
|  | MF403209 |
|  | MF403215 |
|  | MF403230 |
|  | MF403231 |
|  | MF403205 |
|  | MF403221 |
|  | MF403210 |
|  | MF403206 |
|  | MF403218 |
|  | KX534327 |
|  | JX299836 |
|  | JX299622 |
|  | JX301143 |
|  | GQ462050 |
|  | GQ462058 |
|  | GQ462095 |
|  | GQ462157 |
|  | GQ462267 |
|  | GQ462353 |
|  | KU498445 |
|  | KU498321 |
|  | KU498306 |
|  | KU498378 |
|  | KU498383 |
|  | KU498432 |
|  | KU498433 |
|  | KU498451 |
|  | KU498454 |
|  | KU498333 |
|  | KU498399 |
|  | KU498401 |
|  | KU498317 |
|  | KX662399 |
|  | KX662389 |
|  | KX662408 |
|  | KX662386 |
|  | KX662395 |
|  | KX662387 |
|  | KX662376 |
|  | KX662374 |
|  | MF109697 |
|  | DQ205254 |
|  | DQ205255 |
|  | DQ205256 |
|  | DQ205257 |
|  | DQ207944 |
|  | DQ205258 |
|  | DQ205259 |
|  | DQ205262 |
|  | DQ205263 |
|  | DQ205266 |
|  | DQ205267 |
|  | DQ205268 |
|  | DQ205270 |
|  | DQ205271 |
|  | DQ205274 |
|  | DQ205275 |
|  | DQ205276 |
|  | DQ205277 |
|  | DQ205278 |
|  | DQ205279 |
|  | DQ205281 |
|  | DQ205283 |
|  | DQ205285 |
|  | DQ205286 |
|  | DQ205287 |
|  | DQ205289 |
|  | DQ205290 |
|  | DQ205291 |
|  | DQ205292 |
|  | DQ205294 |
|  | EU683760 |
|  | EU861977 |
|  | AB356229 |
|  | AB868197 |
|  | AB866087 |
|  | AB865607 |
|  | LC162249 |
|  | KF874569 |
|  | KF874567 |
|  | MK228831 |
|  | MK228824 |
|  | MK228826 |
|  | MK228829 |
|  | MG798935 |
|  | MG798936 |
|  | MG798937 |
|  | MG798938 |
|  | MG798939 |
|  | MG798940 |
|  | MG798941 |
|  | MG798942 |
|  | MG798943 |
|  | MG798944 |
|  | MG798945 |
|  | MG798946 |
|  | MG798947 |
|  | MG798948 |
|  | MG798949 |
|  | MG798950 |
|  | MG798951 |
|  | MG798952 |
|  | MG798953 |
|  | MG798954 |
|  | MG798955 |
|  | MG798956 |
|  | MG798957 |
|  | MG798958 |
|  | MG798959 |
|  | MG798960 |
|  | MG798961 |
|  | MG798962 |
|  | MG798963 |
|  | MG798964 |
|  | MG798965 |
|  | MG798966 |
|  | MG798967 |
|  | MG798968 |
|  | MG798969 |
|  | MG798970 |
|  | MG798971 |
|  | MG799021 |
|  | MG799022 |
|  | MG799023 |
|  | MG799053 |
|  | MG799054 |
|  | MG799055 |
|  | MG799025 |
|  | MG799026 |
|  | MG799027 |
|  | MG799028 |
|  | MG799029 |
|  | MG799030 |
|  | MG799031 |
|  | MG799032 |
|  | MG799033 |
|  | MG799034 |
|  | MG799035 |
|  | MG799036 |
|  | MG799037 |
|  | MG799038 |
|  | MG799039 |
|  | MG799040 |
|  | MG799041 |
|  | MG799042 |
|  | MG799043 |
|  | MG799044 |
|  | MG799046 |
|  | MG799047 |
|  | MG799048 |
|  | MG799049 |
|  | MG799051 |
|  | MG799052 |
|  | KM820414 |
|  | KX155621 |
|  | EU345695 |
|  | EU345696 |
|  | EU345697 |
|  | EU345698 |
|  | EU345699 |
|  | EF589042 |
|  | EF589043 |
|  | EF589039 |
|  | EF589044 |
|  | EF589040 |
|  | EF589041 |
|  | KF498472 |
|  | KF512380 |
|  | KF512389 |
|  | KF512390 |
|  | KF512391 |
|  | KF498481 |
|  | KF498483 |
|  | KF498484 |
|  | KF512381 |
|  | KF512382 |
|  | KF512383 |
|  | KF512384 |
|  | KF512386 |
|  | KF512387 |
|  | KF512388 |
|  | KF498462 |
|  | KF498466 |
|  | KF498478 |
|  | KF498479 |
|  | KF498480 |
|  | KF498464 |
|  | KF498485 |
|  | KF498463 |
|  | KF498451 |
|  | KF498458 |
|  | KF498455 |
|  | KF498449 |
|  | KF498461 |
|  | KF498452 |
|  | KF498454 |
|  | KF498453 |
|  | JX946628 |
|  | JX946629 |
|  | JX946631 |
|  | JX946632 |
|  | JX946636 |
|  | JX946641 |
|  | JX946643 |
|  | JX946645 |
|  | JX946650 |
|  | JX946651 |
|  | JX946652 |
|  | JX946653 |
|  | JX299932 |
|  | JX300086 |
|  | JX300393 |
|  | JX300936 |
|  | GU945087 |
|  | GU945097 |
|  | GU945098 |
|  | GU945099 |
|  | GU945104 |
|  | GU945110 |
|  | GU945112 |
|  | GU945115 |
|  | GU945118 |
|  | GU945120 |
|  | GU945128 |
|  | GU945130 |
|  | GU945148 |
|  | GU945074 |
|  | GU945075 |
|  | GU945077 |
|  | GU945079 |
|  | GU945080 |
|  | GU945082 |
|  | GU945090 |
|  | GU945091 |
|  | GU945092 |
|  | GU945096 |
|  | GU945101 |
|  | GU945102 |
|  | GU945106 |
|  | GU945107 |
|  | GU945108 |
|  | GU945109 |
|  | GU945111 |
|  | GU945113 |
|  | GU945127 |
|  | GU945132 |
|  | GU945140 |
|  | GU945141 |
|  | GU945142 |
|  | GU945143 |
|  | GU945145 |
|  | GU945076 |
|  | GU945081 |
|  | GU945083 |
|  | GU945084 |
|  | GU945086 |
|  | GU945088 |
|  | GU945089 |
|  | GU945093 |
|  | GU945094 |
|  | GU945095 |
|  | GU945100 |
|  | GU945103 |
|  | GU945105 |
|  | GU945119 |
|  | GU945121 |
|  | GU945122 |
|  | GU945123 |
|  | GU945125 |
|  | GU945133 |
|  | GU945135 |
|  | GU945137 |
|  | GU945138 |
|  | GU945139 |
|  | GU945146 |
|  | GU945147 |
|  | GU945151 |
|  | GU945153 |
|  | GU945155 |
|  | GU945159 |
|  | GU945172 |
|  | GU945176 |
|  | GU945178 |
|  | GU945180 |
|  | GU945181 |
|  | GU945182 |
|  | GU945188 |
|  | GU945160 |
|  | GU945161 |
|  | GU945162 |
|  | GU945163 |
|  | GU945164 |
|  | GU945165 |
|  | GU945166 |
|  | GU945169 |
|  | GU945170 |
|  | GU945171 |
|  | GU945173 |
|  | GU945174 |
|  | GU945175 |
|  | GU945185 |
|  | GU945186 |
|  | GU945189 |
|  | GU945190 |
|  | GU945194 |
|  | GU945150 |
|  | GU945152 |
|  | GU945154 |
|  | GU945156 |
|  | GU945157 |
|  | GU945167 |
|  | GU945177 |
|  | GU945179 |
|  | GU945191 |
|  | GU945193 |
|  | GU945195 |
|  | JX299593 |
|  | JX299608 |
|  | JX299681 |
|  | JX299684 |
|  | JX299707 |
|  | JX299792 |
|  | JX299810 |
|  | JX299828 |
|  | JX299858 |
|  | JX299879 |
|  | JX299892 |
|  | JX299935 |
|  | JX299989 |
|  | JX300032 |
|  | JX300042 |
|  | JX300119 |
|  | JX300132 |
|  | JX300141 |
|  | JX300157 |
|  | JX300276 |
|  | JX300283 |
|  | JX300373 |
|  | JX300384 |
|  | JX300460 |
|  | JX300496 |
|  | JX300565 |
|  | JX300598 |
|  | JX300726 |
|  | JX300769 |
|  | JX300837 |
|  | JX300886 |
|  | JX300938 |
|  | JX300967 |
|  | JX300990 |
|  | JX301030 |
|  | JX301059 |
|  | JX301097 |
|  | JX301122 |
|  | AF400677 |
|  | AF400675 |
|  | AF400680 |
|  | AF400678 |
|  | AF400676 |
|  | AF400679 |
|  | LC311909 |
|  | LC311925 |
|  | LC311986 |
|  | LC311987 |
|  | LC311996 |
|  | GQ400299 |
|  | GQ399425 |
|  | JX300431 |
|  | AB747473 |
|  | AB747538 |
|  | GU906861 |
|  | GU906877 |
|  | KM284597 |
|  | KM284224 |
|  | KM284407 |
|  | KM284426 |
|  | KM057365 |
|  | KM057367 |
|  | KM057370 |
|  | KM283901 |
|  | KM283912 |
|  | KM284017 |
|  | KM284132 |
|  | KM284137 |
|  | KM284472 |
|  | KM284659 |
|  | KM283942 |
|  | KM284021 |
|  | KM284157 |
|  | KM284169 |
|  | KM284226 |
|  | KM284227 |
|  | KM284264 |
|  | KM284333 |
|  | KM284337 |
|  | KM284401 |
|  | KM057357 |
|  | KM057361 |
|  | KM057362 |
|  | KM057364 |
|  | KM057369 |
|  | KM283902 |
|  | KM283984 |
|  | KM284113 |
|  | KM284125 |
|  | KM284222 |
|  | KM284346 |
|  | KM284367 |
|  | KM284438 |
|  | KM284475 |
|  | KM284546 |
|  | KM284548 |
|  | KT340132 |
|  | KT340187 |
|  | KT340202 |
|  | GQ400498 |
|  | EU345595 |
|  | EU345596 |
|  | MK029139 |
|  | MK029140 |
|  | MK029141 |
|  | MK029142 |
|  | MK029143 |
|  | MK029144 |
|  | MK029145 |
|  | MK029146 |
|  | MK029147 |
|  | MK029148 |
|  | MK029149 |
|  | MK029150 |
|  | MK029151 |
|  | MK029152 |
|  | MK029153 |
|  | MK029154 |
|  | MK029155 |
|  | MK029156 |
|  | MK029157 |
|  | MK029158 |
|  | MK029159 |
|  | MK029160 |
|  | MK029161 |
|  | MK029162 |
|  | MK029163 |
|  | MK029164 |
|  | MK029165 |
|  | MK029166 |
|  | MK029167 |
|  | MK029168 |
|  | MK029169 |
|  | MK029170 |
|  | MK029171 |
|  | MK029172 |
|  | MK029173 |
|  | MK029174 |
|  | MK029175 |
|  | MK029176 |
|  | MK029177 |
|  | MK029178 |
|  | MK029179 |
|  | MK029180 |
|  | MK029181 |
|  | MK029182 |
|  | MK029183 |
|  | MK029184 |
|  | MK029185 |
|  | MK029186 |
|  | MK029187 |
|  | MK029188 |
|  | MK029189 |
|  | MK029190 |
|  | MK029191 |
|  | MK029192 |
|  | MK029193 |
|  | MK029194 |
|  | MK029195 |
|  | MK029196 |
|  | MK029197 |
|  | MK029198 |
|  | MK029199 |
|  | MK029200 |
|  | MK029201 |
|  | MK029202 |
|  | MK029203 |
|  | MK029204 |
|  | MK029205 |
|  | MK029208 |
|  | MK029209 |
|  | MK029210 |
|  | MK029211 |
|  | MK029212 |
|  | MK029213 |
|  | MK029214 |
|  | MK029215 |
|  | MK029216 |
|  | MK029217 |
|  | MK029218 |
|  | MK029219 |
|  | MK029220 |
|  | MK029221 |
|  | MK029222 |
|  | MK029223 |
|  | MK029224 |
|  | MK029225 |
|  | MK029226 |
|  | MK029227 |
|  | MK029228 |
|  | MK029229 |
|  | MK029230 |
|  | MK029231 |
|  | MK029232 |
|  | MK029233 |
|  | MK029234 |
|  | MK029235 |
|  | MK029236 |
|  | MK029237 |
|  | MK029238 |
|  | MK029239 |
|  | MK029240 |
|  | MK029241 |
|  | MK029242 |
|  | MK029243 |
|  | MK029244 |
|  | MK029245 |
|  | MK029246 |
|  | MK029247 |
|  | MK029248 |
|  | MK029250 |
|  | MK029251 |
|  | MK029252 |
|  | MK029253 |
|  | MK029254 |
|  | MK029255 |
|  | MK029256 |
|  | MK029257 |
|  | MK029258 |
|  | MK029259 |
|  | MK029260 |
|  | MK029261 |
|  | MK029262 |
|  | MK029263 |
|  | MK029264 |
|  | MK029265 |
|  | MK029266 |
|  | MK029267 |
|  | MK029268 |
|  | MK029269 |
|  | MK029270 |
|  | MK029271 |
|  | MK029272 |
|  | MK029273 |
|  | MK029274 |
|  | MK029275 |
|  | MK029276 |
|  | MK029277 |
|  | MK029278 |
|  | MK029279 |
|  | MK029280 |
|  | MK029281 |
|  | MK029282 |
|  | MK029283 |
|  | MK029284 |
|  | MK029285 |
|  | MK029286 |
|  | MK029287 |
|  | MK029288 |
|  | MK029289 |
|  | MK029290 |
|  | MK029291 |
|  | MK029292 |
|  | MK029293 |
|  | MK029294 |
|  | MK029295 |
|  | MK029296 |
|  | MK029298 |
|  | MK029299 |
|  | MK029300 |
|  | MK029301 |
|  | MK029302 |
|  | MK029303 |
|  | MK029304 |
|  | MK029305 |
|  | MK029306 |
|  | MK029307 |
|  | MK029308 |
|  | MK029309 |
|  | MK029310 |
|  | MK029311 |
|  | MK029312 |
|  | MK029313 |
|  | MK029314 |
|  | MK029315 |
|  | MK029316 |
|  | MK029317 |
|  | MK029318 |
|  | MK029319 |
|  | MK029320 |
|  | MK029321 |
|  | MK029322 |
|  | MK029323 |
|  | MK029324 |
|  | MK029325 |
|  | MK029326 |
|  | MK029327 |
|  | MK029328 |
|  | KX517452 |
|  | KX530768 |
|  | KX530767 |
|  | FJ491427 |
|  | FJ491430 |
|  | FJ515323 |
|  | FJ491437 |
|  | FJ491438 |
|  | FJ491439 |
|  | FJ491440 |
|  | FJ491442 |
|  | FJ491443 |
|  | FJ491444 |
|  | FJ491453 |
|  | FJ491446 |
|  | FJ491447 |
|  | FJ491449 |
|  | FJ491450 |
|  | FJ491451 |
|  | EU345598 |
|  | KX788813 |
|  | KX788815 |
|  | KX517454 |
|  | KX517456 |
|  | EU345599 |
|  | EU345600 |
|  | EU345601 |
|  | EU345602 |
|  | EU345603 |
|  | EU345604 |
|  | EU345605 |
|  | EU345606 |
|  | EU345841 |
|  | EU345842 |
|  | EU345843 |
|  | EU345844 |
|  | KX517446 |
|  | EU345700 |
|  | KX517408 |
|  | KX517412 |
|  | KX517423 |
|  | KX517426 |
|  | KX517429 |
|  | KX517435 |
|  | KX517436 |
|  | KX517437 |
|  | EF103193 |
|  | EU345791 |
|  | EU345866 |
|  | EU345867 |
|  | EU345868 |
|  | EU345869 |
|  | EU345870 |
|  | EU345792 |
|  | EU345871 |
|  | EU345872 |
|  | EU345873 |
|  | EU345874 |
|  | EU345875 |
|  | EU345876 |
|  | EU345794 |
|  | EU345877 |
|  | EU345878 |
|  | EU345879 |
|  | EU345880 |
|  | EU345795 |
|  | EU345881 |
|  | EU345882 |
|  | EU345796 |
|  | EU345883 |
|  | EU345797 |
|  | EU345884 |
|  | EU345885 |
|  | EU345886 |
|  | EU345887 |
|  | EU345888 |
|  | EU345889 |
|  | EU345890 |
|  | EU345891 |
|  | EU345892 |
|  | EU345893 |
|  | EU345894 |
|  | EU345921 |
|  | EU345806 |
|  | EU345924 |
|  | EU345925 |
|  | EU345926 |
|  | EU345936 |
|  | EU345940 |
|  | EU345943 |
|  | EU345826 |
|  | EU345827 |
|  | EU345828 |
|  | EU345829 |
|  | EU345830 |
|  | EU345780 |
|  | EU345781 |
|  | EU345784 |
|  | EU345785 |
|  | EU345854 |
|  | EU345786 |
|  | EU345855 |
|  | EU345787 |
|  | EU345858 |
|  | EU345788 |
|  | EU345859 |
|  | EU345789 |
|  | KM247291 |
|  | KM247292 |
|  | KM247293 |
|  | KM247294 |
|  | KM247296 |
|  | KM247297 |
|  | KM247298 |
|  | KM247299 |
|  | KM247300 |
|  | EU345614 |
|  | EU345615 |
|  | EU345616 |
|  | EU345617 |
|  | EU345618 |
|  | EU345619 |
|  | EU345620 |
|  | EU345621 |
|  | EU345622 |
|  | EU345623 |
|  | EU345624 |
|  | EU345625 |
|  | EU345626 |
|  | EU345627 |
|  | EU345628 |
|  | EU345629 |
|  | EU345630 |
|  | EU345631 |
|  | EU345632 |
|  | EU345633 |
|  | EU345634 |
|  | EU345635 |
|  | EU345638 |
|  | EU345640 |
|  | EU345642 |
|  | EU345643 |
|  | EU345644 |
|  | EU345646 |
|  | EU345647 |
|  | EU345648 |
|  | EU345650 |
|  | EU345651 |
|  | EU345653 |
|  | EU345655 |
|  | EU345656 |
|  | EU345657 |
|  | EU345659 |
|  | EU345660 |
|  | EU345661 |
|  | EU345662 |
|  | EU345663 |
|  | EU345664 |
|  | EU345666 |
|  | EU345667 |
|  | EU345668 |
|  | EU345669 |
|  | EU345670 |
|  | EU345671 |
|  | EU345673 |
|  | EU345674 |
|  | EU345675 |
|  | EU345676 |
|  | EU345677 |
|  | EU345678 |
|  | EU345679 |
|  | EU345680 |
|  | EU345681 |
|  | EU345682 |
|  | EU345684 |
|  | EU345685 |
|  | EU345686 |
|  | EU345687 |
|  | EU345689 |
|  | EU345690 |
|  | EU345691 |
|  | EU345692 |
|  | EU345693 |
|  | EU345694 |
|  | EU345701 |
|  | EU345847 |
|  | EU345702 |
|  | EU345703 |
|  | EU345704 |
|  | EU345705 |
|  | EU345706 |
|  | EU345707 |
|  | EU345708 |
|  | EU345709 |
|  | EU345711 |
|  | EU345713 |
|  | EU345714 |
|  | EU345715 |
|  | EU345716 |
|  | EF545108 |
|  | EU345717 |
|  | EU345718 |
|  | EU345719 |
|  | EU345721 |
|  | EU345722 |
|  | EU345852 |
|  | EU345807 |
|  | EU345808 |
|  | EU345809 |
|  | EU345810 |
|  | EU345811 |
|  | EU345812 |
|  | EU345813 |
|  | EU345814 |
|  | EU345815 |
|  | EU345817 |
|  | EU345818 |
|  | EU345819 |
|  | EU345820 |
|  | EU345821 |
|  | EU345822 |
|  | EU345823 |
|  | EU345824 |
|  | EU345825 |
|  | EU345725 |
|  | EU345726 |
|  | EU345727 |
|  | EU345728 |
|  | EU345729 |
|  | EU345730 |
|  | EU345731 |
|  | EU345732 |
|  | EU345733 |
|  | EU345734 |
|  | EU345735 |
|  | EU345736 |
|  | EU345737 |
|  | EU345738 |
|  | EU345739 |
|  | EU345740 |
|  | EU345741 |
|  | EU345743 |
|  | EU345744 |
|  | EU345745 |
|  | EU345746 |
|  | EU345748 |
|  | EU345749 |
|  | EU345750 |
|  | EU345752 |
|  | EU345753 |
|  | EU345754 |
|  | EU345755 |
|  | EU345756 |
|  | EU345758 |
|  | EU345759 |
|  | EU345760 |
|  | EU345761 |
|  | EU345762 |
|  | EU345763 |
|  | EU345764 |
|  | EU345765 |
|  | EU345766 |
|  | EU345767 |
|  | EU345770 |
|  | EU345771 |
|  | EU345773 |
|  | JQ292892 |
|  | EU345799 |
|  | EU345800 |
|  | EU345895 |
|  | EU345801 |
|  | EU345897 |
|  | EU345898 |
|  | EU345899 |
|  | EU345802 |
|  | EU345900 |
|  | EU345901 |
|  | EU345803 |
|  | EU345902 |
|  | EU345903 |
|  | EU345904 |
|  | EU345905 |
|  | AY500393 |
|  | EF545110 |
|  | EF545109 |
|  | HQ896679 |
|  | JQ292895 |
|  | GQ867609 |
|  | GQ867624 |
|  | GQ867628 |
|  | GQ867630 |
|  | GQ867616 |
|  | GQ867632 |
|  | GQ867633 |
|  | GQ867620 |
|  | GQ867627 |
|  | GQ867608 |
|  | GQ867629 |
|  | GQ867631 |
|  | GQ867607 |
|  | GQ867614 |
|  | GQ867612 |
|  | GQ867604 |
|  | GQ867636 |
|  | GQ867626 |
|  | GQ867615 |
|  | GQ867625 |
|  | GQ867623 |
|  | GQ867621 |
|  | GQ867613 |
|  | GQ867597 |
|  | GQ867610 |
|  | GQ867611 |
|  | GQ867599 |
|  | GQ867622 |
|  | GQ867602 |
|  | GQ867601 |
|  | GQ867598 |
|  | GQ867600 |
|  | FJ009713 |
|  | FJ009714 |
|  | FJ009715 |
|  | FJ009716 |
|  | FJ009717 |
|  | FJ009718 |
|  | FJ009719 |
|  | FJ009720 |
|  | FJ009721 |
|  | FJ009722 |
|  | FJ009723 |
|  | FJ009724 |
|  | FJ009725 |
|  | FJ009726 |
|  | FJ009727 |
|  | FJ009728 |
|  | JQ292896 |
|  | FJ009729 |
|  | FJ009730 |
|  | FJ009731 |
|  | JQ292897 |
|  | FJ009732 |
|  | FJ009733 |
|  | FJ009734 |
|  | JQ292898 |
|  | FJ009735 |
|  | FJ009736 |
|  | FJ009737 |
|  | JQ292899 |
|  | FJ009738 |
|  | FJ009739 |
|  | FJ009740 |
|  | FJ009741 |
|  | FJ009742 |
|  | FJ009743 |
|  | FJ009744 |
|  | FJ009745 |
|  | FJ009746 |
|  | FJ009747 |
|  | FJ009748 |
|  | FJ009749 |
|  | FJ009750 |
|  | FJ009751 |
|  | FJ009752 |
|  | FJ009753 |
|  | FJ009754 |
|  | FJ009755 |
|  | FJ009756 |
|  | FJ009757 |
|  | FJ009759 |
|  | FJ009760 |
|  | FJ009761 |
|  | FJ009762 |
|  | FJ009763 |
|  | FJ009764 |
|  | FJ009765 |
|  | FJ009766 |
|  | FJ009767 |
|  | FJ009768 |
|  | FJ009770 |
|  | FJ009772 |
|  | FJ009773 |
|  | FJ009774 |
|  | FJ009758 |
|  | FJ009776 |
|  | FJ009775 |
|  | FJ009777 |
|  | FJ009778 |
|  | FJ009779 |
|  | FJ009783 |
|  | FJ009784 |
|  | FJ009786 |
|  | FJ009789 |
|  | FJ009790 |
|  | FJ009791 |
|  | FJ009792 |
|  | FJ009793 |
|  | FJ009794 |
|  | FJ009795 |
|  | FJ009796 |
|  | FJ009797 |
|  | FJ009798 |
|  | FJ009799 |
|  | FJ009800 |
|  | FJ009801 |
|  | FJ009802 |
|  | FJ009803 |
|  | FJ009804 |
|  | FJ009805 |
|  | FJ009806 |
|  | FJ009807 |
|  | JQ292900 |
|  | FJ009808 |
|  | FJ009809 |
|  | FJ009811 |
|  | FJ009812 |
|  | FJ009813 |
|  | HM345536 |
|  | HM345537 |
|  | HM345538 |
|  | HM345539 |
|  | HM345542 |
|  | HM345543 |
|  | HM345545 |
|  | HM345547 |
|  | HM345548 |
|  | HM345549 |
|  | HM345550 |
|  | FJ822073 |
|  | HM345551 |
|  | FJ822074 |
|  | HM345552 |
|  | HM345554 |
|  | HM345555 |
|  | HM345556 |
|  | HM345557 |
|  | HM345558 |
|  | HM345559 |
|  | HM345560 |
|  | HM345561 |
|  | HM345563 |
|  | FJ822094 |
|  | FJ822095 |
|  | FJ822096 |
|  | FJ822105 |
|  | FJ822120 |
|  | GQ867635 |
|  | JQ292891 |
|  | FJ822055 |
|  | FJ822064 |
|  | FJ822065 |
|  | FJ822066 |
|  | FJ822067 |
|  | FJ822068 |
|  | FJ822069 |
|  | FJ822070 |
|  | FJ822071 |
|  | FJ822072 |
|  | FJ822056 |
|  | FJ822076 |
|  | FJ822082 |
|  | FJ822083 |
|  | FJ822057 |
|  | FJ822084 |
|  | FJ822085 |
|  | FJ822086 |
|  | FJ822087 |
|  | HM345565 |
|  | FJ822091 |
|  | HM345566 |
|  | FJ822092 |
|  | FJ822093 |
|  | HM345568 |
|  | FJ822058 |
|  | HM345569 |
|  | HM345570 |
|  | HM345571 |
|  | FJ822097 |
|  | HM345572 |
|  | FJ822098 |
|  | HM345574 |
|  | FJ822101 |
|  | HM345575 |
|  | FJ822102 |
|  | HM345576 |
|  | FJ822103 |
|  | FJ822059 |
|  | FJ822104 |
|  | HM345578 |
|  | FJ822106 |
|  | HM345579 |
|  | FJ822107 |
|  | FJ822108 |
|  | FJ822109 |
|  | FJ822110 |
|  | FJ822111 |
|  | FJ822113 |
|  | FJ822060 |
|  | FJ822115 |
|  | FJ822116 |
|  | FJ822117 |
|  | FJ822118 |
|  | FJ822119 |
|  | FJ822121 |
|  | FJ822122 |
|  | FJ822123 |
|  | FJ822061 |
|  | FJ822124 |
|  | FJ822125 |
|  | FJ822126 |
|  | FJ822127 |
|  | FJ822128 |
|  | FJ822129 |
|  | FJ822130 |
|  | FJ822131 |
|  | FJ822132 |
|  | FJ822133 |
|  | FJ822062 |
|  | FJ822134 |
|  | FJ822063 |
|  | KF716491 |
|  | KF716492 |
|  | HQ395272 |
|  | HQ395273 |
|  | HQ395274 |
|  | HQ395275 |
|  | HQ412529 |
|  | HQ412531 |
|  | HQ412532 |
|  | HQ412533 |
|  | HQ412534 |
|  | HQ412528 |
|  | HQ412535 |
|  | HQ412536 |
|  | HQ412537 |
|  | HQ412538 |
|  | HQ412517 |
|  | HQ412518 |
|  | HQ412519 |
|  | HQ412520 |
|  | HQ412521 |
|  | HQ412522 |
|  | HQ412523 |
|  | HQ412525 |
|  | HQ412526 |
|  | HQ412524 |
|  | HQ412527 |
|  | HQ424156 |
|  | HQ424158 |
|  | HQ424157 |
|  | KR233365 |
|  | HQ424155 |
|  | HQ449391 |
|  | KR233366 |
|  | HQ449393 |
|  | HQ449395 |
|  | HQ449397 |
|  | HQ449398 |
|  | HQ449399 |
|  | HQ449400 |
|  | HQ449401 |
|  | HQ449402 |
|  | HQ449403 |
|  | KR233367 |
|  | KR233368 |
|  | KR233370 |
|  | KR233371 |
|  | KR233331 |
|  | KR233332 |
|  | KR233333 |
|  | KR233334 |
|  | KR233335 |
|  | KY238318 |
|  | KY238321 |
|  | KY238323 |
|  | KY238326 |
|  | KY238327 |
|  | KY238328 |
|  | KY238331 |
|  | FJ864679 |
|  | KR233326 |
|  | KR233327 |
|  | KR233328 |
|  | KR233329 |
|  | KR233330 |
|  | JQ292893 |
|  | JQ292894 |
|  | HQ161915 |
|  | HQ161916 |
|  | HQ161917 |
|  | HQ161918 |
|  | HQ161920 |
|  | HQ161921 |
|  | HQ161922 |
|  | JN129277 |
|  | JN673405 |
|  | KR233382 |
|  | KR233383 |
|  | KR233384 |
|  | KR233385 |
|  | HQ161923 |
|  | HQ161924 |
|  | HQ161925 |
|  | HQ161926 |
|  | HQ161927 |
|  | HQ161929 |
|  | HQ161928 |
|  | HQ161930 |
|  | KR233372 |
|  | KR233374 |
|  | KR233376 |
|  | KR233377 |
|  | KR233380 |
|  | KR233381 |
|  | KR233395 |
|  | KR233396 |
|  | KR233397 |
|  | KR233398 |
|  | KR233399 |
|  | KR233400 |
|  | KR233401 |
|  | KR233402 |
|  | KR233403 |
|  | KR233404 |
|  | KR233336 |
|  | KR233337 |
|  | KR233338 |
|  | KR233339 |
|  | KR233340 |
|  | KR233341 |
|  | KR233342 |
|  | KR233343 |
|  | KR233344 |
|  | KR233345 |
|  | KR233386 |
|  | KR233387 |
|  | HQ698896 |
|  | KR233390 |
|  | KR233392 |
|  | KR233393 |
|  | KR233394 |
|  | HQ161911 |
|  | KR233346 |
|  | KR233347 |
|  | KR233348 |
|  | KR233349 |
|  | KR233350 |
|  | KR233351 |
|  | KR233352 |
|  | KR233353 |
|  | KR233354 |
|  | KR233355 |
|  | KR233356 |
|  | HQ161912 |
|  | HQ161913 |
|  | HQ161914 |
|  | KR233357 |
|  | KR233358 |
|  | KR233359 |
|  | KR233360 |
|  | KR233361 |
|  | KR233362 |
|  | KR233363 |
|  | KR233364 |
|  | HQ129997 |
|  | HQ129998 |
|  | HQ129996 |
|  | HQ129990 |
|  | HQ129999 |
|  | HQ129989 |
|  | HQ129991 |
|  | HQ129992 |
|  | KJ870322 |
|  | FJ822077 |
|  | FJ822078 |
|  | FJ822079 |
|  | FJ822080 |
|  | JX290229 |
|  | JX290230 |
|  | HQ129993 |
|  | JX290231 |
|  | HQ129994 |
|  | HQ129995 |
|  | JX290232 |
|  | HQ130000 |
|  | JX290208 |
|  | JX290233 |
|  | JX290209 |
|  | JX290210 |
|  | JX290211 |
|  | JX290212 |
|  | JX290213 |
|  | JX290214 |
|  | JX290215 |
|  | JX290216 |
|  | JX290217 |
|  | JX290218 |
|  | JX290219 |
|  | JX290220 |
|  | JX500696 |
|  | JX290221 |
|  | JX290222 |
|  | JX290223 |
|  | JX500695 |
|  | JX290224 |
|  | JX290225 |
|  | JX290227 |
|  | JX290228 |
|  | JX290234 |
|  | JX290235 |
|  | JX290236 |
|  | JX290238 |
|  | KJ870288 |
|  | KJ870290 |
|  | KJ870291 |
|  | KJ870292 |
|  | KJ870293 |
|  | KJ870294 |
|  | KJ870295 |
|  | KJ870280 |
|  | KJ870296 |
|  | KJ870309 |
|  | KJ870314 |
|  | KJ870315 |
|  | KJ870316 |
|  | KJ870317 |
|  | KJ870339 |
|  | KJ870278 |
|  | KJ870279 |
|  | KJ870281 |
|  | KJ870282 |
|  | KJ870284 |
|  | KJ870287 |
|  | KJ870289 |
|  | KJ870323 |
|  | KJ870325 |
|  | KJ870327 |
|  | KJ870328 |
|  | KJ870330 |
|  | KJ870333 |
|  | KJ870341 |
|  | KJ870342 |
|  | KJ870283 |
|  | KJ870285 |
|  | KJ870451 |
|  | KJ870324 |
|  | KJ870321 |
|  | KJ870319 |
|  | KJ870286 |
|  | KJ870306 |
|  | KJ870307 |
|  | KJ870308 |
|  | KJ870269 |
|  | KJ870270 |
|  | KJ870338 |
|  | KJ870268 |
|  | KJ870297 |
|  | KJ870298 |
|  | KJ870299 |
|  | KJ870300 |
|  | KJ870311 |
|  | KJ870312 |
|  | KJ870310 |
|  | KJ870313 |
|  | KJ870348 |
|  | KJ870275 |
|  | KJ870277 |
|  | KJ870272 |
|  | KJ870301 |
|  | KJ870302 |
|  | KJ870466 |
|  | KJ870263 |
|  | KJ870264 |
|  | KJ870575 |
|  | KJ870262 |
|  | KJ870265 |
|  | KJ870266 |
|  | KJ870267 |
|  | KJ870273 |
|  | KJ870274 |
|  | KJ870276 |
|  | KJ870303 |
|  | KJ870304 |
|  | KJ870305 |
|  | KJ870345 |
|  | KJ870346 |
|  | KJ870349 |
|  | KJ870404 |
|  | KJ870405 |
|  | KJ870406 |
|  | KJ870431 |
|  | KJ870433 |
|  | KJ870458 |
|  | KJ870460 |
|  | KJ870462 |
|  | KJ870464 |
|  | KJ870465 |
|  | KF374864 |
|  | KF374865 |
|  | KF374866 |
|  | KF374867 |
|  | KF374868 |
|  | KF374869 |
|  | KF374870 |
|  | KF374871 |
|  | KF374872 |
|  | KF374873 |
|  | KF374874 |
|  | KF374875 |
|  | KF374876 |
|  | KF374914 |
|  | JX500694 |
|  | KF374851 |
|  | KF374852 |
|  | KF374853 |
|  | KF374854 |
|  | KF374855 |
|  | KF374856 |
|  | KF374857 |
|  | KF374858 |
|  | KF374859 |
|  | KF374860 |
|  | KF374861 |
|  | KF374862 |
|  | KF374863 |
|  | KJ870377 |
|  | KJ870378 |
|  | KJ870379 |
|  | KJ870381 |
|  | KJ870382 |
|  | KJ870383 |
|  | KJ870417 |
|  | KJ870418 |
|  | KJ870419 |
|  | KJ870420 |
|  | KJ870421 |
|  | KJ870423 |
|  | KJ870424 |
|  | KJ870394 |
|  | KJ870425 |
|  | KJ870391 |
|  | KJ870385 |
|  | KJ870396 |
|  | KJ870386 |
|  | KJ870387 |
|  | KJ870393 |
|  | KJ870395 |
|  | KJ870435 |
|  | KJ870436 |
|  | KJ870438 |
|  | KJ870392 |
|  | KJ870440 |
|  | KJ870444 |
|  | KJ870445 |
|  | KJ870446 |
|  | KJ870447 |
|  | KJ870448 |
|  | KJ870449 |
|  | KJ870450 |
|  | KJ870452 |
|  | KJ870454 |
|  | KJ870455 |
|  | KJ870456 |
|  | KJ870372 |
|  | KJ870373 |
|  | KJ870401 |
|  | KJ870409 |
|  | KJ870416 |
|  | KJ870427 |
|  | KJ870428 |
|  | KJ870369 |
|  | KJ870469 |
|  | KJ870474 |
|  | KJ870475 |
|  | KJ870476 |
|  | KJ870389 |
|  | KJ870350 |
|  | KJ870360 |
|  | KJ870361 |
|  | KJ870362 |
|  | KJ870363 |
|  | KJ870408 |
|  | KJ870426 |
|  | KJ870429 |
|  | KJ870471 |
|  | KJ870473 |
|  | KJ870351 |
|  | KJ870359 |
|  | KJ870366 |
|  | KJ870390 |
|  | KJ870364 |
|  | KJ870365 |
|  | KJ870479 |
|  | KJ870480 |
|  | KJ870481 |
|  | KJ870482 |
|  | KJ870443 |
|  | KJ870453 |
|  | KJ870442 |
|  | KJ870441 |
|  | KJ870388 |
|  | KJ870437 |
|  | KJ870439 |
|  | KJ870368 |
|  | KJ870399 |
|  | KJ870380 |
|  | KJ870422 |
|  | KJ870430 |
|  | KJ870461 |
|  | KJ870376 |
|  | KJ870352 |
|  | KJ870459 |
|  | KJ870354 |
|  | KJ870463 |
|  | KJ870432 |
|  | KJ870367 |
|  | KJ870472 |
|  | KJ870353 |
|  | KJ870355 |
|  | KJ870375 |
|  | JX141198 |
|  | JX141199 |
|  | JX141201 |
|  | JX141202 |
|  | JX141200 |
|  | JX141203 |
|  | JX141204 |
|  | JX141205 |
|  | JX141206 |
|  | JX141207 |
|  | JX141208 |
|  | MK589458 |
|  | MK589465 |
|  | MK589469 |
|  | MK589645 |
|  | MK589646 |
|  | MK589647 |
|  | MK589648 |
|  | MK589649 |
|  | MK589650 |
|  | MK589651 |
|  | MK589652 |
|  | MK589653 |
|  | MH666355 |
|  | MH666356 |
|  | MH666357 |
|  | MH666358 |
|  | MH666359 |
|  | MH666361 |
|  | MH666362 |
|  | MH666363 |
|  | MH666365 |
|  | MH666366 |
|  | MH666367 |
|  | MH666368 |
|  | MH666369 |
|  | MH666372 |
|  | MH666467 |
|  | MH666491 |
|  | MH666497 |
|  | MH666503 |
|  | MH666504 |
|  | MH666508 |
|  | MH666514 |
|  | MH666516 |
|  | MH666519 |
|  | MH666534 |
|  | MH666560 |
|  | MH666566 |
|  | MH666576 |
|  | MH666577 |
|  | MH666578 |
|  | MH666580 |
|  | MH666581 |
|  | MH666582 |
|  | MH666584 |
|  | MH666585 |
|  | MH666586 |
|  | MH666587 |
|  | MH666588 |
|  | MH666589 |
|  | MH666591 |
|  | MH666592 |
|  | MH666593 |
|  | MH666594 |
|  | MH666595 |
|  | MH666597 |
|  | MH666658 |
|  | MH666677 |
|  | MH666679 |
|  | MH666681 |
|  | MH666683 |
|  | MH666686 |
|  | MH666719 |
|  | MH666720 |
|  | MH666724 |
|  | MH666726 |
|  | MH666739 |
|  | MH666812 |
|  | MH666824 |
|  | MH666828 |
|  | MH666842 |
|  | MH666853 |
|  | MH666863 |
|  | MH666864 |
|  | MH666866 |
|  | MH666870 |
|  | MH666872 |
|  | MH666883 |
|  | MH666891 |
|  | MH666892 |
|  | MH666893 |
|  | MH666918 |
|  | MH666950 |
|  | MH666953 |
|  | MH666981 |
|  | MH666983 |
|  | MH666987 |
|  | MH666991 |
|  | MH666992 |
|  | MH667001 |
|  | MH667018 |
|  | MH667027 |
|  | MH667051 |
|  | MH667104 |
|  | MH667125 |
|  | MH667152 |
|  | MH667159 |
|  | MH667171 |
|  | MH667176 |
|  | MH667252 |
|  | MH667253 |
|  | MH667254 |
|  | MH667255 |
|  | JX141209 |
|  | JX141210 |
|  | JX141211 |
|  | JX141212 |
|  | JX141213 |
|  | JX141214 |
|  | JX141215 |
|  | JX141216 |
|  | JX141217 |
|  | JX141218 |
|  | JX141219 |
|  | KY238337 |
|  | KY238342 |
|  | KY238344 |
|  | KY238346 |
|  | KY238350 |
|  | KJ722070 |
|  | KJ722071 |
|  | KJ722072 |
|  | KJ722073 |
|  | KP659664 |
|  | KP659665 |
|  | KP659666 |
|  | KP659668 |
|  | KP659669 |
|  | KP659670 |
|  | KP659671 |
|  | KP659672 |
|  | KP659673 |
|  | KP659674 |
|  | KP659675 |
|  | KP659677 |
|  | KP659678 |
|  | KP659679 |
|  | KP659680 |
|  | KP659681 |
|  | KP659682 |
|  | KP659683 |
|  | KP659684 |
|  | KP659686 |
|  | KP659687 |
|  | KP659688 |
|  | KP659689 |
|  | KP659690 |
|  | KP659691 |
|  | KP659692 |
|  | KP659693 |
|  | KP659694 |
|  | KP659695 |
|  | KP659696 |
|  | KP659697 |
|  | KP659698 |
|  | KP659699 |
|  | KP659700 |
|  | KP659702 |
|  | KP659703 |
|  | KP659704 |
|  | KP659705 |
|  | KP659706 |
|  | KP659707 |
|  | KP659708 |
|  | KP659709 |
|  | KP659710 |
|  | KP659711 |
|  | KP659712 |
|  | KP659713 |
|  | KP659714 |
|  | KP659715 |
|  | KP659716 |
|  | KP659717 |
|  | KP659718 |
|  | KP659719 |
|  | KP659720 |
|  | KP659721 |
|  | KP659722 |
|  | KP659723 |
|  | KP659724 |
|  | KP659725 |
|  | KP659726 |
|  | KP659727 |
|  | KP659728 |
|  | KP659729 |
|  | KP659730 |
|  | KP659731 |
|  | KP659732 |
|  | KP659733 |
|  | KP659734 |
|  | KP659735 |
|  | JX141227 |
|  | JX141228 |
|  | JX141229 |
|  | JX141230 |
|  | JX141231 |
|  | JX141232 |
|  | JX141233 |
|  | KJ870407 |
|  | KJ870484 |
|  | KJ870509 |
|  | KJ870511 |
|  | KF374880 |
|  | KF374897 |
|  | KF374881 |
|  | KF374882 |
|  | KF374883 |
|  | KF374884 |
|  | KF374885 |
|  | KF374886 |
|  | KF374887 |
|  | KF374877 |
|  | KF374888 |
|  | KF374889 |
|  | KF374890 |
|  | KF374891 |
|  | KF374878 |
|  | KF374879 |
|  | KF374894 |
|  | KF374895 |
|  | KF374896 |
|  | KF374921 |
|  | KF374922 |
|  | KF374923 |
|  | KF374898 |
|  | KF374899 |
|  | KF374900 |
|  | KF374901 |
|  | KF374924 |
|  | KF374925 |
|  | KF374926 |
|  | KF374927 |
|  | KF374902 |
|  | KF374903 |
|  | KF374904 |
|  | KF374905 |
|  | KF374906 |
|  | KF374907 |
|  | KF374909 |
|  | KF374910 |
|  | KF374911 |
|  | KF374912 |
|  | KF374915 |
|  | KF374916 |
|  | KF374917 |
|  | KF374918 |
|  | KF374919 |
|  | KF374920 |
|  | KJ870501 |
|  | KJ870518 |
|  | KJ870545 |
|  | KJ870507 |
|  | KJ870526 |
|  | KJ870527 |
|  | KJ870528 |
|  | KJ870571 |
|  | KJ870572 |
|  | KJ870574 |
|  | KJ870602 |
|  | KJ870603 |
|  | KJ870604 |
|  | KJ870521 |
|  | KJ870523 |
|  | KJ870524 |
|  | KJ870587 |
|  | KJ870588 |
|  | KJ870590 |
|  | KJ870591 |
|  | KJ870592 |
|  | KJ870605 |
|  | KJ870493 |
|  | KJ870494 |
|  | KJ870495 |
|  | KJ870496 |
|  | KJ870497 |
|  | KJ870498 |
|  | KJ870499 |
|  | KJ870500 |
|  | KJ870502 |
|  | KJ870504 |
|  | KJ870505 |
|  | KJ870506 |
|  | KJ870508 |
|  | KJ870510 |
|  | KJ870536 |
|  | KJ870537 |
|  | KJ870538 |
|  | KJ870539 |
|  | KJ870541 |
|  | KJ870543 |
|  | KJ870544 |
|  | KJ870548 |
|  | KJ870549 |
|  | KJ870576 |
|  | KJ870578 |
|  | KJ870579 |
|  | KJ870580 |
|  | KJ870581 |
|  | KJ870503 |
|  | KJ870582 |
|  | KJ870577 |
|  | KJ870583 |
|  | KJ870584 |
|  | KJ870585 |
|  | KJ870542 |
|  | KJ870489 |
|  | KJ870552 |
|  | KJ870553 |
|  | KJ870554 |
|  | KJ870555 |
|  | KJ870556 |
|  | KJ870557 |
|  | KJ870559 |
|  | KJ870560 |
|  | KJ870586 |
|  | KJ870565 |
|  | KJ870593 |
|  | KJ870595 |
|  | KJ870517 |
|  | KJ870525 |
|  | KJ870532 |
|  | KJ870611 |
|  | KJ870599 |
|  | KJ870600 |
|  | KJ870470 |
|  | KJ870562 |
|  | KJ870558 |
|  | KJ870563 |
|  | KJ870677 |
|  | KJ870531 |
|  | KJ870596 |
|  | KJ870551 |
|  | KJ870589 |
|  | KJ870606 |
|  | KJ870522 |
|  | KJ870483 |
|  | KJ870573 |
|  | KJ870601 |
|  | KJ870529 |
|  | KJ870520 |
|  | KC254581 |
|  | KC254582 |
|  | KC254583 |
|  | MH330347 |
|  | KC254584 |
|  | KC254585 |
|  | KC254587 |
|  | KC254615 |
|  | KC254588 |
|  | KC254589 |
|  | KC254590 |
|  | KC254591 |
|  | KC254592 |
|  | KC254593 |
|  | KC254594 |
|  | KC254595 |
|  | KC254596 |
|  | MH330348 |
|  | KC254597 |
|  | MH330349 |
|  | KC254599 |
|  | MH330350 |
|  | KC254600 |
|  | KC254601 |
|  | KC254602 |
|  | MH330351 |
|  | KC254603 |
|  | KC254604 |
|  | KC254605 |
|  | MH330352 |
|  | KC254606 |
|  | KC254607 |
|  | MH330353 |
|  | KC254608 |
|  | MH330354 |
|  | KC254609 |
|  | MH330355 |
|  | KC254610 |
|  | KC254611 |
|  | KC254612 |
|  | MH330356 |
|  | KC254613 |
|  | MH330358 |
|  | MH330359 |
|  | MH330361 |
|  | MH330363 |
|  | MH330364 |
|  | MH330365 |
|  | KC254617 |
|  | KC254618 |
|  | KC254619 |
|  | MH330367 |
|  | KC254620 |
|  | KC254621 |
|  | KC254622 |
|  | KC254623 |
|  | KC254624 |
|  | KC254625 |
|  | KC254643 |
|  | KC254626 |
|  | KC254627 |
|  | KC254628 |
|  | KC254629 |
|  | KC254630 |
|  | KC254631 |
|  | KC254632 |
|  | KC254633 |
|  | KC254634 |
|  | KC254635 |
|  | KC254636 |
|  | KC254637 |
|  | KC254638 |
|  | KC254639 |
|  | KC254640 |
|  | KC254641 |
|  | KC254642 |
|  | MK589447 |
|  | MK589449 |
|  | MK589450 |
|  | MK589453 |
|  | MK589456 |
|  | MK589457 |
|  | MK589460 |
|  | MK589461 |
|  | MK589463 |
|  | MK589464 |
|  | MK589467 |
|  | MK589468 |
|  | MK589485 |
|  | MK589487 |
|  | MK589499 |
|  | MK589502 |
|  | MK589505 |
|  | MK589508 |
|  | MK589512 |
|  | MK589513 |
|  | MK589537 |
|  | MK589545 |
|  | MK589548 |
|  | MK589549 |
|  | MK589550 |
|  | MK589556 |
|  | MK589565 |
|  | MK589576 |
|  | MK589578 |
|  | MK589586 |
|  | MK589588 |
|  | MK589590 |
|  | MK589591 |
|  | MK589594 |
|  | MK589595 |
|  | MK589596 |
|  | MK589597 |
|  | MK589598 |
|  | MK589599 |
|  | MK589600 |
|  | MK589601 |
|  | MK589602 |
|  | MK589603 |
|  | MK589604 |
|  | MK589605 |
|  | MK589606 |
|  | MK589607 |
|  | MK589608 |
|  | MK589609 |
|  | MK589612 |
|  | MK589613 |
|  | MK589614 |
|  | MK589615 |
|  | MK589616 |
|  | MK589617 |
|  | MK589618 |
|  | MK589619 |
|  | MK589620 |
|  | MK589621 |
|  | MK589622 |
|  | MK589623 |
|  | MK589624 |
|  | MK589625 |
|  | MK589626 |
|  | MK589627 |
|  | MK589628 |
|  | MK589629 |
|  | MK589630 |
|  | MK589631 |
|  | MK589632 |
|  | MK589633 |
|  | MK589634 |
|  | MK589635 |
|  | MK589636 |
|  | MK589637 |
|  | MK589638 |
|  | MK589639 |
|  | MK589640 |
|  | MK589641 |
|  | MK589642 |
|  | MK589643 |
|  | MK589644 |
|  | MK589654 |
|  | MK589655 |
|  | MH666360 |
|  | MH666371 |
|  | MH666373 |
|  | MH666374 |
|  | MH666375 |
|  | MH666378 |
|  | MH666379 |
|  | MH666380 |
|  | MH666381 |
|  | MH666382 |
|  | MH666383 |
|  | MH666384 |
|  | MH666386 |
|  | MH666387 |
|  | MH666388 |
|  | MH666389 |
|  | MH666390 |
|  | MH666391 |
|  | MH666392 |
|  | MH666394 |
|  | MH666396 |
|  | MH666397 |
|  | MH666398 |
|  | MH666399 |
|  | MH666400 |
|  | MH666401 |
|  | MH666402 |
|  | MH666403 |
|  | MH666404 |
|  | MH666405 |
|  | MH666406 |
|  | MH666407 |
|  | MH666408 |
|  | MH666409 |
|  | MH666410 |
|  | MH666411 |
|  | MH666412 |
|  | MH666413 |
|  | MH666414 |
|  | MH666415 |
|  | MH666416 |
|  | MH666417 |
|  | MH666419 |
|  | MH666420 |
|  | MH666421 |
|  | MH666422 |
|  | MH666423 |
|  | MH666424 |
|  | MH666425 |
|  | MH666426 |
|  | MH666427 |
|  | MH666430 |
|  | MH666431 |
|  | MH666432 |
|  | MH666434 |
|  | MH666435 |
|  | MH666437 |
|  | MH666438 |
|  | MH666439 |
|  | MH666440 |
|  | MH666442 |
|  | MH666443 |
|  | MH666444 |
|  | MH666445 |
|  | MH666446 |
|  | MH666447 |
|  | MH666450 |
|  | MH666451 |
|  | MH666452 |
|  | MH666453 |
|  | MH666454 |
|  | MH666455 |
|  | MH666456 |
|  | MH666457 |
|  | MH666458 |
|  | MH666459 |
|  | MH666461 |
|  | MH666462 |
|  | MH666463 |
|  | MH666464 |
|  | MH666466 |
|  | MH666468 |
|  | MH666469 |
|  | MH666470 |
|  | MH666471 |
|  | MH666472 |
|  | MH666473 |
|  | MH666475 |
|  | MH666476 |
|  | MH666477 |
|  | MH666478 |
|  | MH666479 |
|  | MH666480 |
|  | MH666481 |
|  | MH666482 |
|  | MH666483 |
|  | MH666484 |
|  | MH666485 |
|  | MH666488 |
|  | MH666489 |
|  | MH666492 |
|  | MH666493 |
|  | MH666494 |
|  | MH666496 |
|  | MH666499 |
|  | MH666500 |
|  | MH666501 |
|  | MH666502 |
|  | MH666506 |
|  | MH666510 |
|  | MH666511 |
|  | MH666512 |
|  | MH666513 |
|  | MH666515 |
|  | MH666517 |
|  | MH666518 |
|  | MH666520 |
|  | MH666521 |
|  | MH666526 |
|  | MH666527 |
|  | KF257851 |
|  | KF257852 |
|  | KF257853 |
|  | KF257854 |
|  | KF257855 |
|  | KF257850 |
|  | KF257856 |
|  | KF257857 |
|  | KF257858 |
|  | KF257859 |
|  | KF257860 |
|  | KF257861 |
|  | KF257862 |
|  | KF257863 |
|  | KF257864 |
|  | KF257865 |
|  | KF257866 |
|  | KF257867 |
|  | KF257868 |
|  | KF257869 |
|  | KF257871 |
|  | KF257872 |
|  | KF257874 |
|  | KF257875 |
|  | KF257876 |
|  | KF257877 |
|  | KC156532 |
|  | KC156533 |
|  | KC208003 |
|  | KC208004 |
|  | KC208005 |
|  | KC509787 |
|  | KC509791 |
|  | KC509827 |
|  | KC509769 |
|  | KC509828 |
|  | KC509830 |
|  | KC509831 |
|  | KC509832 |
|  | KC509771 |
|  | KC509833 |
|  | KC509834 |
|  | KC509772 |
|  | KC509773 |
|  | KC509774 |
|  | KC509835 |
|  | KC509836 |
|  | KC509775 |
|  | KC509776 |
|  | KC509777 |
|  | KC509778 |
|  | KC509779 |
|  | KC509780 |
|  | KC509781 |
|  | KC509782 |
|  | KC509783 |
|  | KC509784 |
|  | KC509785 |
|  | KC509786 |
|  | KC509788 |
|  | KC509789 |
|  | KC509886 |
|  | KC509790 |
|  | KC509792 |
|  | KC509793 |
|  | KC509794 |
|  | KC509795 |
|  | KC509796 |
|  | KC509797 |
|  | KC509798 |
|  | KC509799 |
|  | KC509800 |
|  | KC509801 |
|  | KC509802 |
|  | KC509804 |
|  | KC509806 |
|  | KC509807 |
|  | KC509808 |
|  | KC509809 |
|  | KC509810 |
|  | KC509811 |
|  | KC509812 |
|  | KC509813 |
|  | KC509814 |
|  | KC509815 |
|  | KC509816 |
|  | KC509817 |
|  | KC509818 |
|  | KC509819 |
|  | KC509820 |
|  | KC509821 |
|  | KC509822 |
|  | KC509824 |
|  | KC509825 |
|  | KC509826 |
|  | KC665925 |
|  | KC509837 |
|  | KC509838 |
|  | KC509839 |
|  | KC665926 |
|  | KC509840 |
|  | KC665927 |
|  | KC665928 |
|  | KC509841 |
|  | KC665929 |
|  | KC509842 |
|  | KC665930 |
|  | KC665931 |
|  | KC509843 |
|  | KC509844 |
|  | KC665932 |
|  | KC509845 |
|  | KF177159 |
|  | KC665933 |
|  | KC509846 |
|  | KC665934 |
|  | KC509847 |
|  | KF177160 |
|  | KC509848 |
|  | KC509849 |
|  | KF177161 |
|  | KC509850 |
|  | KC665936 |
|  | KC665937 |
|  | KC665938 |
|  | KC665939 |
|  | KF177162 |
|  | KC665916 |
|  | KC665917 |
|  | KC665918 |
|  | KC665919 |
|  | KC665921 |
|  | KC665943 |
|  | KC665922 |
|  | KF177163 |
|  | KC665923 |
|  | KC665924 |
|  | KC509851 |
|  | KC509852 |
|  | KC509853 |
|  | KC509855 |
|  | KC509860 |
|  | KC509864 |
|  | KF177166 |
|  | KC509872 |
|  | KC509875 |
|  | KC509879 |
|  | KC509880 |
|  | KC665942 |
|  | KC509882 |
|  | KC509883 |
|  | KC509884 |
|  | KC509885 |
|  | KJ499552 |
|  | KJ499553 |
|  | KJ499554 |
|  | KJ499555 |
|  | KJ499556 |
|  | KJ499557 |
|  | KJ499558 |
|  | KJ499559 |
|  | KJ499560 |
|  | KJ499561 |
|  | KJ499562 |
|  | KJ499563 |
|  | KJ499564 |
|  | KJ499565 |
|  | KJ499566 |
|  | KJ499568 |
|  | KJ499569 |
|  | KJ499570 |
|  | KJ499571 |
|  | KJ499572 |
|  | KJ499573 |
|  | KJ499574 |
|  | KJ499575 |
|  | KJ499576 |
|  | KJ499577 |
|  | KJ499578 |
|  | KJ499579 |
|  | KJ499581 |
|  | KJ499582 |
|  | KJ499583 |
|  | KJ499584 |
|  | KJ499585 |
|  | KJ499586 |
|  | KJ499587 |
|  | KJ499588 |
|  | KJ499589 |
|  | KJ499590 |
|  | KJ499591 |
|  | KJ499592 |
|  | KJ499609 |
|  | KJ499610 |
|  | KJ499611 |
|  | KJ499612 |
|  | KJ499614 |
|  | KJ499615 |
|  | KJ499616 |
|  | KJ499617 |
|  | KJ499618 |
|  | KJ722074 |
|  | KJ722075 |
|  | KJ722076 |
|  | KJ722077 |
|  | KJ722078 |
|  | KJ722079 |
|  | KJ722082 |
|  | KJ722137 |
|  | KJ722083 |
|  | KJ722084 |
|  | KJ722085 |
|  | KJ722086 |
|  | KJ722087 |
|  | KJ722088 |
|  | KJ722089 |
|  | KJ722090 |
|  | KJ722091 |
|  | KJ722092 |
|  | KJ722093 |
|  | KJ722094 |
|  | KJ722095 |
|  | KJ722096 |
|  | KJ722097 |
|  | KJ722098 |
|  | KJ722099 |
|  | KJ722100 |
|  | KJ722101 |
|  | KJ722102 |
|  | KJ722103 |
|  | KJ722104 |
|  | KJ722106 |
|  | KJ722107 |
|  | KJ722108 |
|  | KJ722109 |
|  | KJ722113 |
|  | KJ722114 |
|  | KJ722115 |
|  | KJ722116 |
|  | KJ722117 |
|  | KJ722118 |
|  | KJ722119 |
|  | KJ722120 |
|  | KJ722138 |
|  | KJ722121 |
|  | KJ722139 |
|  | KJ722122 |
|  | KJ722123 |
|  | KJ722124 |
|  | KJ722125 |
|  | KJ722126 |
|  | KJ722127 |
|  | KJ722128 |
|  | KJ722129 |
|  | KJ722130 |
|  | KJ722131 |
|  | KJ722133 |
|  | KJ722134 |
|  | KJ722135 |
|  | KJ722136 |
|  | KF971919 |
|  | KF971920 |
|  | KF205384 |
|  | KF971923 |
|  | KF205386 |
|  | KF971926 |
|  | KF971929 |
|  | KF971933 |
|  | KF971936 |
|  | KF205388 |
|  | KF971937 |
|  | KF971938 |
|  | KF205390 |
|  | KF971945 |
|  | JX141220 |
|  | JX141221 |
|  | JX141223 |
|  | JX141224 |
|  | JX141225 |
|  | JX141226 |
|  | KJ461974 |
|  | KJ870663 |
|  | KJ870625 |
|  | KJ870630 |
|  | KJ870668 |
|  | KJ870626 |
|  | KJ870629 |
|  | KJ870628 |
|  | KJ870662 |
|  | KJ870667 |
|  | KJ870684 |
|  | KJ870631 |
|  | KJ870632 |
|  | KJ870633 |
|  | KJ870647 |
|  | KJ870659 |
|  | KJ870660 |
|  | KJ870678 |
|  | KJ870681 |
|  | KJ870683 |
|  | KJ870618 |
|  | KJ870619 |
|  | KJ870620 |
|  | KJ870621 |
|  | KJ870622 |
|  | KJ870658 |
|  | KJ870624 |
|  | KJ870661 |
|  | KJ870665 |
|  | KJ870666 |
|  | KJ870669 |
|  | KJ870656 |
|  | KJ870657 |
|  | KJ870616 |
|  | KJ870655 |
|  | KJ870642 |
|  | KJ870636 |
|  | KJ870643 |
|  | KJ870672 |
|  | KJ870688 |
|  | KJ870689 |
|  | KJ870613 |
|  | KJ870645 |
|  | KJ870641 |
|  | KJ870670 |
|  | KJ870637 |
|  | KJ870614 |
|  | KJ870651 |
|  | KJ870646 |
|  | KJ870648 |
|  | KJ870682 |
|  | KU557639 |
|  | MG902950 |
|  | KU557641 |
|  | KU557642 |
|  | KU557643 |
|  | KU670321 |
|  | MG902951 |
|  | KU645873 |
|  | KU645875 |
|  | KU933331 |
|  | MH330337 |
|  | KU645876 |
|  | MH330338 |
|  | KU645879 |
|  | KU933335 |
|  | MH330340 |
|  | KU645856 |
|  | MH330341 |
|  | KU645881 |
|  | KU645882 |
|  | KU645883 |
|  | KU933336 |
|  | MH330342 |
|  | KU645858 |
|  | MH330343 |
|  | KU933339 |
|  | KU933340 |
|  | KU670325 |
|  | KU670326 |
|  | MH330344 |
|  | KU670327 |
|  | KU670328 |
|  | MH330345 |
|  | KU645861 |
|  | MH330346 |
|  | KU645866 |
|  | KU645868 |
|  | KU645871 |
|  | KF257881 |
|  | KF257882 |
|  | KY514100 |
|  | KY514099 |
|  | KY514105 |
|  | KY514101 |
|  | KY514102 |
|  | KY514106 |
|  | KY514103 |
|  | MK589452 |
|  | MK589455 |
|  | MK589474 |
|  | MK589481 |
|  | MK589488 |
|  | MK589490 |
|  | MK589492 |
|  | MK589495 |
|  | MK589500 |
|  | MK589509 |
|  | MK589511 |
|  | MK589518 |
|  | MK589522 |
|  | MK589535 |
|  | MK589540 |
|  | MK589543 |
|  | MK589544 |
|  | MK589546 |
|  | MK589547 |
|  | MK589551 |
|  | MK589553 |
|  | MK589555 |
|  | MK589557 |
|  | MK589558 |
|  | MK589559 |
|  | MK589560 |
|  | MK589561 |
|  | MK589562 |
|  | MK589563 |
|  | MK589564 |
|  | MK589566 |
|  | MK589567 |
|  | MK589568 |
|  | MK589569 |
|  | MK589570 |
|  | MK589571 |
|  | MK589572 |
|  | MK589573 |
|  | MK589574 |
|  | MK589575 |
|  | MK589577 |
|  | MK589579 |
|  | MK589580 |
|  | MK589581 |
|  | MK589582 |
|  | MK589583 |
|  | MK589584 |
|  | MK589585 |
|  | MK589587 |
|  | MK589589 |
|  | MK589592 |
|  | MK589593 |
|  | MK589610 |
|  | MK589611 |
|  | KF257879 |
|  | KF257880 |
|  | KF257878 |
|  | MH666428 |
|  | MH666429 |
|  | MH666522 |
|  | MH666523 |
|  | MH666524 |
|  | MH666525 |
|  | MH666528 |
|  | MH666529 |
|  | MH666530 |
|  | MH666531 |
|  | MH666532 |
|  | MH666533 |
|  | MH666537 |
|  | MH666538 |
|  | MH666539 |
|  | MH666540 |
|  | MH666541 |
|  | MH666542 |
|  | MH666543 |
|  | MH666544 |
|  | MH666545 |
|  | MH666546 |
|  | MH666547 |
|  | MH666548 |
|  | MH666549 |
|  | MH666550 |
|  | MH666551 |
|  | MH666552 |
|  | MH666553 |
|  | MH666554 |
|  | MH666555 |
|  | MH666556 |
|  | MH666557 |
|  | MH666558 |
|  | MH666559 |
|  | MH666561 |
|  | MH666562 |
|  | MH666563 |
|  | MH666564 |
|  | MH666565 |
|  | MH666567 |
|  | MH666568 |
|  | MH666569 |
|  | MH666570 |
|  | MH666571 |
|  | MH666572 |
|  | MH666574 |
|  | MH666579 |
|  | MH666583 |
|  | MH666590 |
|  | MH666596 |
|  | MH666598 |
|  | MH666599 |
|  | MH666601 |
|  | MH666602 |
|  | MH666603 |
|  | MH666604 |
|  | MH666605 |
|  | MH666606 |
|  | MH666608 |
|  | MH666610 |
|  | MH666611 |
|  | MH666612 |
|  | MH666613 |
|  | MH666614 |
|  | MH666615 |
|  | MH666616 |
|  | MH666617 |
|  | MH666618 |
|  | MH666619 |
|  | MH666620 |
|  | MH666621 |
|  | MH666622 |
|  | MH666623 |
|  | MH666624 |
|  | MH666625 |
|  | MH666626 |
|  | MH666628 |
|  | MH666630 |
|  | MH666631 |
|  | MH666633 |
|  | MH666634 |
|  | MH666635 |
|  | MH666636 |
|  | MH666638 |
|  | MH666639 |
|  | MH666640 |
|  | KX432084 |
|  | KX432085 |
|  | KX432086 |
|  | KX432087 |
|  | KX432088 |
|  | KX432089 |
|  | KX432090 |
|  | KX432091 |
|  | KX432092 |
|  | KX432074 |
|  | KX432075 |
|  | KX432076 |
|  | KX432077 |
|  | MH330370 |
|  | KX432078 |
|  | KX432079 |
|  | KX432080 |
|  | KX432081 |
|  | KX432082 |
|  | KX432094 |
|  | KX432095 |
|  | KX432096 |
|  | KX432097 |
|  | KX432098 |
|  | KX432099 |
|  | KX432100 |
|  | KX432102 |
|  | KX432103 |
|  | KX432104 |
|  | KX432105 |
|  | MH330371 |
|  | KX432107 |
|  | MH330372 |
|  | KX432108 |
|  | KX432109 |
|  | KX432110 |
|  | KX432111 |
|  | MH330373 |
|  | KX432112 |
|  | KX432114 |
|  | KX432115 |
|  | MH330374 |
|  | KX432116 |
|  | MH330375 |
|  | KX432117 |
|  | KX432118 |
|  | KX432119 |
|  | KX432121 |
|  | MH330376 |
|  | KX432122 |
|  | KX432123 |
|  | MH330377 |
|  | KX432124 |
|  | MH330378 |
|  | KX432125 |
|  | KX432126 |
|  | KX432127 |
|  | KJ197216 |
|  | KJ197218 |
|  | KJ197220 |
|  | KJ197223 |
|  | KJ499619 |
|  | KJ499620 |
|  | KJ499621 |
|  | KJ499625 |
|  | KJ499626 |
|  | KJ499628 |
|  | KJ499630 |
|  | KJ499631 |
|  | KJ499632 |
|  | KJ499633 |
|  | KJ499634 |
|  | KJ499635 |
|  | KP659736 |
|  | MF124822 |
|  | MF124823 |
|  | MF124824 |
|  | MF124825 |
|  | MF124827 |
|  | MF124828 |
|  | MF124829 |
|  | MF124830 |
|  | MF124831 |
|  | MF124833 |
|  | MF124835 |
|  | MF124836 |
|  | MF124837 |
|  | MF124838 |
|  | MF124839 |
|  | MF124840 |
|  | MF124841 |
|  | MF124842 |
|  | MF124843 |
|  | MF124845 |
|  | MF124846 |
|  | MF124847 |
|  | MF124848 |
|  | MF124849 |
|  | MF124850 |
|  | MF124851 |
|  | MF124852 |
|  | MF124854 |
|  | MF124857 |
|  | MF124858 |
|  | MF124859 |
|  | MF124860 |
|  | MF124861 |
|  | MF124862 |
|  | MF124863 |
|  | MF124865 |
|  | MF124866 |
|  | MF124867 |
|  | MF124868 |
|  | MF124869 |
|  | MF124871 |
|  | KF257870 |
|  | KF257873 |
|  | KF257884 |
|  | MG211630 |
|  | MG211631 |
|  | KU645872 |
|  | MK510022 |
|  | MK510023 |
|  | MK510016 |
|  | MK510017 |
|  | MK510018 |
|  | MK510077 |
|  | MK510019 |
|  | MK510020 |
|  | KP090089 |
|  | MK589448 |
|  | MK589451 |
|  | MK589459 |
|  | MK589476 |
|  | MK589478 |
|  | MK589480 |
|  | MK589482 |
|  | MK589483 |
|  | MK589484 |
|  | MK589486 |
|  | MK589489 |
|  | MK589491 |
|  | MK589493 |
|  | MK589494 |
|  | MK589496 |
|  | MK589497 |
|  | MK589501 |
|  | MK589503 |
|  | MK589504 |
|  | MK589506 |
|  | MK589507 |
|  | MK589514 |
|  | MK589515 |
|  | MK589516 |
|  | MK589517 |
|  | MK589519 |
|  | MK589520 |
|  | MK589521 |
|  | MK589523 |
|  | MK589524 |
|  | MK589525 |
|  | MK589527 |
|  | MK589528 |
|  | MK589531 |
|  | MK589532 |
|  | MK589533 |
|  | MK589534 |
|  | MK589536 |
|  | MK589538 |
|  | MK589539 |
|  | MK589541 |
|  | KP090077 |
|  | MH666641 |
|  | MH666642 |
|  | MH666643 |
|  | MH666644 |
|  | MH666645 |
|  | MH666646 |
|  | MH666647 |
|  | MH666648 |
|  | MH666649 |
|  | MH666650 |
|  | MH666651 |
|  | MH666652 |
|  | MH666653 |
|  | MH666654 |
|  | MH666655 |
|  | MH666656 |
|  | MH666657 |
|  | MH666659 |
|  | MH666661 |
|  | MH666663 |
|  | MH666664 |
|  | MH666665 |
|  | MH666667 |
|  | MH666668 |
|  | MH666669 |
|  | MH666670 |
|  | MH666671 |
|  | MH666672 |
|  | MH666673 |
|  | MH666674 |
|  | MH666676 |
|  | MH666678 |
|  | MH666680 |
|  | MH666682 |
|  | MH666684 |
|  | MH666685 |
|  | MH666687 |
|  | MH666688 |
|  | MH666689 |
|  | MH666691 |
|  | MH666692 |
|  | MH666694 |
|  | MH666695 |
|  | MH666696 |
|  | MH666697 |
|  | MH666698 |
|  | MH666699 |
|  | MH666700 |
|  | MH666701 |
|  | MH666702 |
|  | MH666703 |
|  | MH666704 |
|  | MH666705 |
|  | MH666706 |
|  | MH666707 |
|  | MH666708 |
|  | MH666709 |
|  | MH666710 |
|  | MH666711 |
|  | MH666712 |
|  | MH666714 |
|  | MH666715 |
|  | MH666718 |
|  | MH666721 |
|  | MH666722 |
|  | MH666723 |
|  | MH666725 |
|  | MH666727 |
|  | MH666728 |
|  | MH666729 |
|  | MH666730 |
|  | MH666731 |
|  | MH666732 |
|  | MH666733 |
|  | MH666734 |
|  | MH666735 |
|  | MH666736 |
|  | MH666737 |
|  | MH666738 |
|  | MH666740 |
|  | MH666741 |
|  | MH666742 |
|  | MH666744 |
|  | MH666745 |
|  | MH666746 |
|  | MH666747 |
|  | MH666748 |
|  | MH666749 |
|  | MH666751 |
|  | MH666752 |
|  | MH666753 |
|  | MH666755 |
|  | MH666756 |
|  | MH666759 |
|  | MH666760 |
|  | MH666761 |
|  | MH666762 |
|  | MH666763 |
|  | MH666764 |
|  | MH666765 |
|  | MH666766 |
|  | MH666768 |
|  | MH666769 |
|  | MH666770 |
|  | MH666771 |
|  | MH666772 |
|  | MH666773 |
|  | MH666774 |
|  | MH666775 |
|  | MH666776 |
|  | MH666777 |
|  | MH666778 |
|  | MH666779 |
|  | MH666780 |
|  | MH666781 |
|  | MH666782 |
|  | MH666783 |
|  | MH666784 |
|  | MH666785 |
|  | MH666786 |
|  | MH666788 |
|  | MH666789 |
|  | MH666790 |
|  | MH666791 |
|  | MH666792 |
|  | MH666795 |
|  | MH666796 |
|  | MH666797 |
|  | MH666798 |
|  | MH666799 |
|  | MH666801 |
|  | MH666802 |
|  | MH666803 |
|  | MH666804 |
|  | MH666805 |
|  | MH666806 |
|  | MH666807 |
|  | MH666808 |
|  | MH666809 |
|  | MH666810 |
|  | MH666811 |
|  | MH666814 |
|  | MH666815 |
|  | MH666816 |
|  | MH666817 |
|  | MH666818 |
|  | MH666819 |
|  | MH666820 |
|  | MH666822 |
|  | MH666825 |
|  | MH666826 |
|  | MH666827 |
|  | MH666829 |
|  | MH666830 |
|  | MH666831 |
|  | MH666832 |
|  | MH666833 |
|  | MH666834 |
|  | MH666835 |
|  | MH666836 |
|  | MH666837 |
|  | MH666838 |
|  | MH666839 |
|  | MH666840 |
|  | MH666841 |
|  | MH666843 |
|  | MH666844 |
|  | MH666845 |
|  | MH666846 |
|  | MH666847 |
|  | KX432128 |
|  | KX432129 |
|  | MH330379 |
|  | MH330380 |
|  | KX432131 |
|  | KX432132 |
|  | KX432135 |
|  | KX432136 |
|  | MH330381 |
|  | KX432137 |
|  | KX432138 |
|  | KX432139 |
|  | KP090073 |
|  | KP090074 |
|  | KP090075 |
|  | KP090076 |
|  | KP090078 |
|  | KP090079 |
|  | KP090080 |
|  | KP090081 |
|  | KP090082 |
|  | KP090083 |
|  | KP090086 |
|  | KP090088 |
|  | KP090092 |
|  | KP090094 |
|  | KP090096 |
|  | KP090099 |
|  | KP090084 |
|  | KP090085 |
|  | KP090066 |
|  | KP090067 |
|  | KP090068 |
|  | KP090069 |
|  | KP090070 |
|  | KP090072 |
|  | KP090087 |
|  | MG763750 |
|  | MG211632 |
|  | MG211633 |
|  | MG211635 |
|  | MG211636 |
|  | MG211637 |
|  | KX147563 |
|  | MK512413 |
|  | KX147558 |
|  | KX147561 |
|  | MK512412 |
|  | KX147562 |
|  | KX147559 |
|  | KX147556 |
|  | MK512414 |
|  | MF668566 |
|  | MF668567 |
|  | MF668568 |
|  | MF668569 |
|  | MF668572 |
|  | MF668573 |
|  | MF668574 |
|  | MF668575 |
|  | MF668576 |
|  | MF668577 |
|  | MF668578 |
|  | MF668579 |
|  | MF668580 |
|  | MF668581 |
|  | MF668582 |
|  | MF668583 |
|  | MF668584 |
|  | MF668585 |
|  | MF668586 |
|  | MF668587 |
|  | MF668588 |
|  | KX147551 |
|  | KY514111 |
|  | KY514112 |
|  | MK512415 |
|  | KY514113 |
|  | KY514114 |
|  | KY514115 |
|  | KY514116 |
|  | KY514117 |
|  | KY514118 |
|  | KY514119 |
|  | KY514120 |
|  | KY514121 |
|  | KY514122 |
|  | KY514123 |
|  | KX147565 |
|  | MK512417 |
|  | KX147564 |
|  | KX147557 |
|  | KX147560 |
|  | KY514110 |
|  | KY514107 |
|  | KY514108 |
|  | KY514109 |
|  | KX147552 |
|  | KX147553 |
|  | KX147555 |
|  | MK510024 |
|  | MK510026 |
|  | MK510027 |
|  | MK510030 |
|  | MK510029 |
|  | KX574399 |
|  | 1 |
|  | KX574400 |
|  | KX574402 |
|  | KX574403 |
|  | KX574404 |
|  | KT121453 |
|  | KT121454 |
|  | KT121455 |
|  | KT121456 |
|  | MK589454 |
|  | MK589462 |
|  | MK589471 |
|  | MK589472 |
|  | MK589475 |
|  | MK589479 |
|  | MK589498 |
|  | MK589529 |
|  | MK589530 |
|  | MK589656 |
|  | MK589657 |
|  | MK589658 |
|  | MK589659 |
|  | MK589660 |
|  | MK589661 |
|  | MK589662 |
|  | MK589663 |
|  | MK589664 |
|  | MK589665 |
|  | MK589666 |
|  | MK589667 |
|  | MK589668 |
|  | MK589669 |
|  | MK589670 |
|  | MK589671 |
|  | MK589672 |
|  | MK589673 |
|  | MK589674 |
|  | MK589675 |
|  | MK589676 |
|  | MK589677 |
|  | MK589678 |
|  | MK589679 |
|  | MK589680 |
|  | MK589681 |
|  | MK589682 |
|  | MK589683 |
|  | MK589684 |
|  | MK589685 |
|  | MK589686 |
|  | MK589690 |
|  | MK589691 |
|  | MK589692 |
|  | MK589693 |
|  | MK589694 |
|  | MH666848 |
|  | MH666849 |
|  | MH666851 |
|  | MH666852 |
|  | MH666854 |
|  | MH666855 |
|  | MH666856 |
|  | MH666857 |
|  | MH666858 |
|  | MH666859 |
|  | MH666860 |
|  | MH666861 |
|  | MH666862 |
|  | MH666865 |
|  | MH666867 |
|  | MH666868 |
|  | MH666869 |
|  | MH666871 |
|  | MH666873 |
|  | MH666875 |
|  | MH666877 |
|  | MH666878 |
|  | MH666879 |
|  | MH666880 |
|  | MH666881 |
|  | MH666882 |
|  | MH666885 |
|  | MH666886 |
|  | MH666887 |
|  | MH666888 |
|  | MH666889 |
|  | MH666890 |
|  | MH666894 |
|  | MH666896 |
|  | MH666897 |
