# Supplementary material for: Origin and evolution of HIV-1 subtype A6
Source: PLoS One. 2021 Dec 13;16(12):e0260604. doi: 10.1371/journal.pone.0260604 (PMC8668117; doi:10.1371/journal.pone.0260604)
Supplement: S3 Table — A1 and A6 sequences were compared using VESPA. A1 and A6 sequences were used as background and query, respectively. (DOCX) [file pone.0260604.s005.docx]

**S3 Table.**

| **Pol** | | | | | | | | |
| --- | --- | --- | --- | --- | --- | --- | --- | --- |
| **Position** | | **Most common AA** | | | | **Occurrence** | | |
|  |  | **A1** | | **A6** | | **A1** | **A6** | |
| **14** | | 0.629 K | | 0.567 R | | 3 E, 1259 K, 4 N, 614 R, 1 T, 104 X, 15 - | 3 E, 746 K, 1 L, 1133 R, 1 S, 113 X, 3 - | |
| **57** | | 0.745 K | | 0.929 R | | 1490 K, 1 N, 466 R, 1 S, 42 X | 91 K, 1858 R, 51 X | |
| **93** | | 0.986 I | | 0.692 L | | 1972 I, 22 L, 5 M, 1 X | 543 I, 1384 L, 1 V, 72 X | |
| **135** | | 0.960 E | | 0.819 D | | 18 A, 35 D, 1920 E, 1 K, 1 Q, 1 R, 1 V, 20 X, 3 - | 18 A, 1638 D, 225 E, 2 G, 2 H, 16 N, 1 S, 1 T, 1 V, 96 X | |
| **138** | | 0.518 T | | 0.638 K | | 119 A, 1 D, 28 E, 26 G, 13 I, 280 K, 31 L, 38 M, 42 N, 20 P, 8 Q, 197 R, 27 S, 1036 T, 133 X, 1 - | 1 A, 22 D, 62 E, 3 G, 1277 K, 13 L, 134 M, 208 N, 15 Q, 45 R, 12 S, 42 T, 166 X | |
| **234** | | 0.562 T | | 0.706 I | | 756 I, 15 K, 6 L, 7 M, 21 R, 1124 T, 23 V, 48 X | 1 A, 1412 I, 3 K, 8 L, 10 M, 19 R, 416 T, 53 V, 78 X | |
| **257** | | 0.965 A | | 0.656 S | | 1931 A, 1 E, 56 S, 10 X, 2 - | 600 A, 2 P, 1313 S, 85 X | |
| **278** | | 0.741 I | | 0.943 V | | 2 D, 1482 I, 5 L, 2 S, 49 T, 390 V, 70 X | 1 E, 1 F, 1 G, 69 I, 3 T, 1886 V, 39 X | |
| **Env** | | | | | | | | |
| **Position** | **Most common AA** | | | | **Occurrence** | | | |
|  | **A1** | | **A6** | | **A1** | | | **A6** |
| 2 | 0.846 R | | 0.991 K | | 1 G, 3 I, 156 K, 909 R, 2 T, 2 X, 2 - | | | 1 E, 697 K, 5 R |
| 3 | 0.882 V | | 0.899 A | | 126 A, 1 L, 948 V | | | 632 A, 71 V |
| 4 | 0.580 M | | 0.920 R | | 1 G, 1 I, 261 K, 623 M, 171 R, 17 T, 1 V | | | 1 G, 30 K, 23 M, 647 R, 2 X |
| 6 | 0.566 I | | 0.919 M | | 1 A, 608 I, 1 K, 35 M, 30 R, 397 T, 2 V, 1 - | | | 5 I, 646 M, 52 T |
| 10 | 0.450 C | | 0.997 Y | | 484 C, 5 F, 1 G, 11 L, 1 R, 124 S, 219 W, 225 Y, 5 - | | | 2 C, 701 Y |
| 14 | 0.533 L | | 0.997 W | | 16 F, 2 G, 573 L, 1 M, 3 S, 1 V, 454 W, 25 - | | | 1 G, 701 W, 1 - |
| 20 | 0.900 I | | 0.716 L | | 3 F, 8 G, 968 I, 12 L, 2 M, 2 T, 80 V | | | 176 F, 24 I, 503 L |
| 21 | 0.980 L | | 0.875 F | | 16 F, 2 I, 1053 L, 2 M, 2 S | | | 1 C, 615 F, 87 L |
| 22 | 0.981 G | | 0.954 W | | 1 A, 2 E, 1055 G, 1 L, 1 V, 12 W, 3 - | | | 4 C, 27 L, 1 M, 671 W |
| 26 | 0.887 I | | 0.873 M | | 50 F, 954 I, 6 L, 43 M, 1 N, 6 T, 15 - | | | 82 I, 1 K, 614 M, 1 T, 5 - |
| 28 | 0.923 S | | 0.987 K | | 2 C, 1 G, 4 K, 64 N, 11 R, 992 S, 1 T | | | 694 K, 9 R |
| 45 | 0.586 K | | 0.930 R | | 37 E, 6 H, 630 K, 4 Q, 397 R, 1 X | | | 48 K, 654 R, 1 X |
| 62 | 0.753 T | | 0.910 K | | 70 A, 8 E, 106 K, 2 L, 23 P, 4 Q, 46 R, 5 S, 810 T, 1 V | | | 640 K, 1 P, 57 R, 5 T |
| 93 | 0.906 N | | 0.629 D | | 98 D, 974 N, 1 S, 1 T, 1 - | | | 442 D, 260 N, 1 - |
| 104 | 0.903 H | | 0.933 Q | | 971 H, 85 N, 18 Q, 1 R | | | 44 H, 2 P, 656 Q, 1 - |
| 131 | 0.370 S | | 0.545 T | | 34 A, 4 D, 14 E, 7 F, 17 G, 126 H, 4 I, 68 K, 2 L, 67 N, 12 Q, 10 R, 398 S, 274 T, 22 V, 1 X, 13 Y, 2 - | | | 213 A, 53 D, 5 I, 2 N, 2 Q, 4 S, 383 T, 41 V |
| 132 | 0.460 N | | 0.593 E | | 92 A, 199 D, 25 E, 1 G, 52 H, 84 K, 495 N, 2 P, 34 Q, 18 S, 37 T, 2 X, 31 Y, 3 - | | | 78 A, 20 D, 417 E, 2 G, 61 K, 73 N, 52 Y |
| 133 | 0.372 V | | 0.896 P | | 89 A, 1 C, 29 D, 3 E, 9 F, 38 H, 181 I, 8 K, 4 L, 105 N, 87 P, 1 R, 10 S, 16 T, 400 V, 3 W, 4 X, 85 Y, 2 - | | | 1 F, 1 I, 68 L, 1 N, 630 P, 1 Q, 1 S |
| 137 | 0.253 N | | 0.821 S | | 111 A, 11 D, 8 E, 26 F, 24 G, 49 H, 29 I, 120 K, 5 L, 272 N, 3 P, 2 Q, 48 R, 83 S, 138 T, 52 V, 1 X, 16 Y, 77 - | | | 5 A, 2 C, 24 G, 2 I, 56 N, 1 P, 1 Q, 19 R, 577 S, 9 T, 4 V, 3 - |
| 138 | 0.316 T | | 0.738 S | | 31 A, 12 D, 4 E, 13 G, 22 I, 8 K, 3 L, 220 N, 5 P, 25 R, 63 S, 340 T, 98 V, 2 X, 15 Y, 214 - | | | 2 A, 1 C, 42 F, 1 G, 24 I, 9 L, 59 N, 5 P, 519 S, 29 T, 5 V, 7 - |
| 153 | 0.979 I | | 0.980 M | | 1 A, 7 C, 4 D, 1 G, 3 N, 2 S, 1 T, 3 V, 1 X, | | | 1 C, 13 S, 689 - |
| 169 | 0.578 Q | | 0.898 K | | 1 E, 4 H, 1 I, 299 K, 1 N, 621 Q, 147 R, 1 T | | | 48 E, 631 K, 1 Q, 20 R, 3 T |
| 170 | 0.843 K | | 0.531 T | | 11 E, 906 K, 12 N, 91 Q, 30 R, 19 S, 6 T | | | 1 A, 52 I, 170 K, 52 L, 50 N, 3 R, 2 S, 373 T |
| 177 | 0.535 R | | 0.950 K | | 1 E, 1 G, 1 I, 491 K, 575 R, 4 S, 1 T, 1 X | | | 1 A, 668 K, 4 N, 30 R |
| 185 | 0.269 E | | 0.721 S | | 49 A, 36 D, 289 E, 144 G, 1 H, 251 K, 211 N, 3 P, 23 Q, 1 R, 57 S, 5 T, 5 - | | | 3 D, 1 E, 6 G, 2 I, 7 K, 142 N, 31 R, 507 S, 2 T, 1 X, 1 - |
| 186 | 0.395 S | | 0.697 N | | 2 A, 25 D, 84 E, 18 G, 5 H, 10 K, 242 N, 11 P, 1 Q, 65 R, 425 S, 18 T, 3 V, 2 X, 2 Y, 162 - | | | 3 D, 20 E, 27 G, 2 K, 490 N, 1 R, 60 S, 26 T, 74 - |
| 191 | 0.576 S | | 0.669 G | | 1 A, 24 D, 6 E, 1 F, 48 G, 44 H, 2 I, 41 K, 1 M, 139 N, 35 Q, 13 R, 619 S, 96 T, 1 Y, 4 - | | | 7 E, 470 G, 1 I, 8 K, 2 N, 111 R, 101 S, 1 T, 1 X, 1 - |
| 192 | 0.384 E | | 0.844 Q | | 1 A, 62 D, 413 E, 4 F, 3 G, 27 H, 2 I, 57 K, 24 L, 99 M, 8 N, 8 P, 242 Q, 11 R, 68 S, 15 T, 2 V, 4 W, 2 X, 19 Y, 4 - | | | 86 E, 3 K, 5 L, 3 P, 593 Q, 7 R, 4 S, 2 T |
| 202 | 0.827 A | | 0.861 T | | 889 A, 5 S, 128 T, 52 V, 1 X | | | 80 A, 1 E, 13 I, 3 S, 605 T, 1 V |
| 203 | 0.973 I | | 0.752 M | | 1046 I, 11 L, 5 M, 4 T, 8 V, 1 X | | | 26 I, 1 L, 529 M, 1 R, 53 T, 93 V |
| 211 | 0.616 S | | 0.873 T | | 1 K, 5 N, 662 S, 407 T | | | 1 A, 1 K, 16 N, 70 S, 614 T, 1 V |
| 234 | 0.613 E | | 0.751 N | | 3 A, 90 D, 659 E, 20 G, 188 K, 2 M, 45 N, 22 Q, 3 R, 9 S, 25 T, 3 V, 6 X | | | 9 D, 58 E, 1 I, 82 K, 1 M, 528 N, 1 Q, 5 S, 10 T, 8 Y |
| 270 | 0.405 G | | 0.617 K | | 8 D, 219 E, 435 G, 203 K, 108 N, 21 Q, 35 R, 13 S, 19 T, 14 - | | | 4 D, 185 E, 29 G, 434 K, 1 N, 48 Q, 1 R, 1 - |
| 281 | 0.760 N | | 0.929 D | | 2 A, 230 D, 5 E, 5 H, 2 I, 817 N, 7 Q, 6 S, 1 - | | | 653 D, 48 N, 2 - |
| 283 | 0.755 A | | 0.595 G | | 812 A, 1 D, 24 G, 12 H, 14 I, 2 K, 1 N, 7 S, 50 T, 151 V, 1 - | | | 7 A, 418 G, 1 H, 5 N, 241 S, 28 T, 1 V, 2 - |
| 285 | 0.465 N | | 0.990 I | | 473 I, 99 L, 2 M, 500 V, 1 X | | | 696 I, 1 L, 6 V |
| 291 | 0.292 N | | 0.744 T | | 152 A, 1 C, 158 D, 1 G, 6 I, 44 K, 1 L, 1 M, 314 N, 10 R, 8 S, 246 T, 129 V, 2 X, 1 Y, 1 - | | | 2 A, 2 D, 114 K, 58 N, 1 Q, 1 S, 523 T, 2 - |
| 295 | 0.159 R | | 0.920 N | | 21 A, 99 E, 53 G, 3 H, 97 I, 120 K, 5 L, 2 M, 137 N, 63 P, 145 Q, 171 R, 19 S, 123 T, 9 V, 2 W, 2 X, 3 Y, 1 - | | | 4 D, 43 K, 647 N, 8 S, 1 - |
| 297 | 0.727 N | | 0.987 T | | 11 D, 17 E, 14 I, 7 K, 781 N, 2 R, 51 S, 189 T, 1 X, 1 Y, 1 - | | | 2 A, 1 I, 5 N, 694 T, 1 - |
| 299 | 0.506 T | | 0.649 I | | 13 A, 72 I, 411 N, 20 P, 9 S, 544 T, 5 V, 1 - | | | 456 I, 55 N, 1 S, 162 T, 28 V, 1 - |
| 302 | 0.745 N | | 0.807 G | | 2 A, 4 D, 169 G, 2 H, 801 N, 7 R, 74 S, 2 T, 13 Y, 1 - | | | 3 D, 567 G, 17 N, 113 S, 2 X, 1 - |
| 307 | 0.580 K | | 0.956 T | | 1 D, 20 E, 623 K, 1 M, 40 Q, 110 R, 279 T, 1 - | | | 11 A, 19 K, 672 T, 1 - |
| 316 | 0.597 A | | 0.853 T | | 642 A, 1 I, 2 K, 3 R, 139 S, 266 T, 18 V, 4 - | | | 102 A, 600 T, 1 X |
| 323 | 0.966 I | | 0.963 V | | 1038 I, 5 M, 1 P, 29 V, 2 - | | | 25 I, 677 V, 1 - |
| 331 | 0.682 H | | 0.518 Y | | 24 F, 733 H, 15 Q, 4 R, 9 S, 1 W, 288 Y, 1 - | | | 5 F, 333 H, 1 S, 364 Y |
| 337 | 0.377 S | | 0.622 A | | 197 A, 20 E, 14 G, 6 H, 1 I, 98 K, 10 L, 4 P, 13 Q, 40 R, 405 S, 252 T, 12 V, 1 X, 2 - | | | 437 A, 46 E, 5 K, 1 P, 27 S, 162 T, 25 V |
| 338 | 0.527 E | | 0.707 A | | 90 A, 114 D, 566 E, 7 G, 8 H, 1 I, 111 K, 8 L, 2 M, 30 N, 57 Q, 12 R, 37 S, 22 T, 7 V, 3 - | | | 497 A, 2 D, 106 E, 36 K, 53 N, 6 T, 1 V, 2 - |
| 346 | 0.967 V | | 0.986 I | | 2 A, 1 E, 29 I, 1 L, 1 T, 1040 V, 1 - | | | 693 I, 1 K, 2 L, 1 M, 1 T, 5 V |
| 347 | 0.567 A | | 0.984 S | | 609 A, 1 E, 14 G, 2 I, 11 K, 1 M, 3 R, 46 S, 15 T, 370 V, 1 X, 1 Y, 1 - | | | 5 G, 6 R, 692 S |
| 353 | 0.471 H | | 0.899 Y | | 5 D, 2 E, 506 H, 28 I, 6 K, 11 L, 12 M, 7 N, 12 Q, 2 R, 3 T, 476 Y, 5 - | | | 1 D, 67 H, 3 Q, 632 Y |
| 362 | 0.324 K | | 0.532 N | | 4 A, 5 D, 46 E, 4 F, 157 G, 8 H, 348 K, 2 M, 71 N, 16 P, 89 Q, 31 R, 15 S, 3 T, 22 V, 1 W, 253 - | | | 8 A, 43 D, 3 E, 5 G, 56 H, 22 K, 374 N, 2 P, 2 Q, 76 S, 3 T, 1 V, 2 Y, 106 - |
| 371 | 0.939 I | | 0.954 V | | 1009 I, 24 L, 3 T, 38 V, 1 - | | | 1 A, 1 E, 30 I, 671 V |
| 388 | 0.908 S | | 0.909 T | | 22 A, 1 E, 3 I, 4 K, 976 S, 68 T, 1 V | | | 60 A, 3 S, 639 T, 1 - |
| 389 | 0.425 G | | 0.519 D | | 99 A, 56 D, 79 E, 457 G, 1 H, 2 I, 46 K, 2 L, 37 N, 1 Q, 21 R, 197 S, 77 T | | | 133 A, 365 D, 64 E, 7 G, 41 K, 87 N, 2 Q, 1 T, 2 V, 1 X |
| 396 | 0.273 N | | 0.612 D | | 5 A, 87 D, 148 E, 8 F, 73 G, 32 H, 29 I, 23 K, 18 L, 9 M, 293 N, 39 P, 26 Q, 63 R, 38 S, 59 T, 58 V, 2 W, 51 Y, 14 - | | | 430 D, 25 E, 91 G, 2 H, 7 K, 1 L, 115 N, 8 R, 7 S, 1 T, 1 V, 1 X, 12 Y, 2 - |
| 397 | 0.251 N | | 0.643 E | | 31 A, 47 D, 64 E, 135 F, 169 G, 31 H, 38 I, 22 K, 33 L, 1 M, 270 N, 28 P, 4 Q, 28 R, 63 S, 24 T, 13 V, 35 W, 1 X, 21 Y, 17 - | | | 1 A, 15 D, 452 E, 78 G, 1 H, 31 K, 45 N, 2 R, 2 S, 73 T, 3 - |
| 401 | 0.196 E | | 0.404 V | | 15 A, 1 C, 22 D, 211 E, 2 F, 32 G, 1 H, 35 I, 61 K, 21 L, 33 M, 103 N, 5 P, 24 Q, 70 R, 81 S, 140 T, 92 V, 17 W, 5 Y, 104 - | | | 33 A, 105 D, 1 E, 1 F, 6 G, 8 I, 4 N, 1 Q, 6 S, 90 T, 284 V, 1 W, 1 X, 162 - |
| 402 | 0.448 S | | 0.714 T | | 8 A, 17 D, 45 E, 6 F, 29 G, 6 H, 6 I, 4 K, 9 L, 12 M, 66 N, 26 P, 102 Q, 93 R, 110 S, 482 T, 39 V, 4 W, 11 - | | | 5 A, 1 D, 4 E, 1 H, 96 I, 1 K, 65 N, 21 Q, 5 S, 502 T, 1 X, 1 - |
| 406 | 0.247 E | | 0.467 Q | | 14 A, 32 D, 265 E, 97 G, 2 H, 4 I, 172 K, 9 L, 8 M, 119 N, 19 P, 23 Q, 13 R, 215 S, 41 T, 36 V, 6 - | | | 22 A, 2 D, 117 E, 78 G, 25 K, 1 L, 3 M, 11 N, 328 Q, 4 R, 16 S, 89 T, 4 V, 2 X, 1 - |
| 407 | 0.455 S | | 0.585 A | | 28 A, 28 D, 100 E, 1 F, 8 G, 1 H, 16 I, 11 K, 64 L, 6 M, 90 N, 118 P, 4 Q, 7 R, 489 S, 88 T, 6 V, 2 X, 2 Y, 6 - | | | 411 A, 62 D, 22 G, 4 I, 28 N, 1 P, 1 R, 4 S, 86 T, 83 V, 1 - |
| 437 | 0.380 Q | | 0.754 K | | 36 A, 113 E, 73 K, 330 P, 408 Q, 107 R, 5 S, 3 T | | | 25 A, 14 E, 1 G, 1 I, 530 K, 13 N, 1 P, 69 Q, 44 R, 5 S |
| 439 | 0.615 V | | 0.481 S | | 19 A, 2 D, 105 E, 83 I, 120 K, 21 L, 3 M, 3 N, 7 P, 6 Q, 9 R, 22 S, 13 T, 661 V, 1 X | | | 1 D, 2 E, 1 H, 161 I, 2 K, 172 N, 1 Q, 338 S, 12 T, 12 V, 1 X |
| 450 | 0.590 I | | 0.997 L | | 634 I, 429 L, 9 M, 2 V, 1 - | | | 2 I, 701 L |
| 458 | 0.359 N | | 0.290 G | | 25 A, 182 D, 51 E, 122 G, 1 H, 21 I, 24 K, 386 N, 6 P, 4 Q, 7 R, 65 S, 38 T, 27 V, 4 X, 112 - | | | 3 A, 11 D, 131 E, 204 G, 1 I, 7 K, 30 N, 115 R, 4 S, 3 T, 1 X, 193 - |
| 469 | 0.854 G | | 0.912 I | | 14 A, 92 E, 918 G, 17 I, 2 Q, 6 S, 12 T, 14 V | | | 4 A, 1 E, 1 G, 641 I, 2 L, 4 Q, 48 T, 2 V |
| 492 | 0.922 L | | 0.994 I | | 80 I, 991 L, 1 P, 3 V | | | 1 E, 699 I, 3 L |
| 498 | 0.431 R | | 0.718 K | | 1 E, 4 G, 24 H, 426 K, 56 Q, 463 R, 100 S, 1 X | | | 505 K, 95 Q, 103 R |
| 511 | 0.906 V | | 0.962 I | | 9 A, 4 F, 62 I, 23 L, 974 V, 3 - | | | 1 F, 676 I, 14 M, 8 V, 4 - |
| 513 | 0.612 I | | 0.910 L | | 1 A, 6 F, 1 G, 658 I, 359 L, 45 M, 4 V, 1 - | | | 3 D, 1 E, 1 G, 7 I, 640 L, 1 R, 46 V, 4 - |
| 516 | 0.960 V | | 0.983 A | | 3 A, 10 F, 2 I, 20 L, 8 M, 1032 V | | | 691 A, 3 L, 1 T, 6 V, 2 X |
| 586 | 0.431 R | | 0.718 K | | 1 E, 4 G, 24 H, 426 K, 56 Q, 463 R, 100 S, 1 X | | | 505 K, 95 Q, 103 R |
| 627 | 0.885 L | | 0.987 M | | 2 I, 951 L, 100 M, 18 Q, 3 R, 1 V | | | 5 I, 3 L, 694 M, 1 Q |
| 642 | 0.172 S | | 0.728 D | | 1 A, 1 C, 109 D, 45 E, 109 G, 14 H, 126 K, 1 L, 2 M, 139 N, 25 Q, 89 R, 185 S, 129 T, 1 V, 1 X, 98 Y | | | 2 A, 512 D, 69 E, 7 G, 1 K, 107 N, 3 Q, 1 R, 1 T |
| 646 | 0.767 E | | 0.526 K | | 5 A, 36 D, 824 E, 4 I, 174 K, 6 N, 18 Q, 2 R, 5 V, 1 X | | | 293 E, 1 I, 370 K, 38 Q, 1 X |
| 715 | 0.903 F | | 0.922 L | | 971 F, 102 L, 1 S, 1 - | | | 54 F, 648 L, 1 X |
| 721 | 0.792 N | | 0.967 H | | 26 A, 3 D, 2 E, 1 G, 21 H, 3 I, 1 K, 1 L, 851 N, 1 Q, 153 S, 5 T, 5 V, 2 - | | | 680 H, 17 N, 1 P, 1 R, 2 S, 2 T |
| 723 | 0.483 R | | 0.986 E | | 4 A, 190 E, 301 G, 5 K, 9 Q, 519 R, 44 T, 2 V, 1 - | | | 3 A, 693 E, 3 G, 2 K, 1 Q, 1 R |
| 725 | 0.813 L | | 0.994 P | | 1 F, 1 I, 874 L, 189 P, 6 R, 3 V, 1 - | | | 1 L, 699 P, 2 T, 1 - |
| 732 | 0.854 E | | 0.817 K | | 5 A, 9 D, 918 E, 86 G, 48 K, 4 N, 3 R, 1 V, 1 - | | | 43 E, 1 G, 574 K, 72 N, 2 R, 11 S |
| 785 | 0.815 H | | 0.916 R | | 876 H, 1 L, 13 Q, 167 R, 1 S, 17 - | | | 58 H, 644 R, 1 - |
| 802 | 0.541 W | | 0.929 G | | 2 E, 408 G, 78 K, 1 R, 1 V, 582 W, 3 - | | | 1 E, 653 G, 42 R, 6 W, 1 X |
| 806 | 0.634 L | | 0.669 G | | 125 A, 1 C, 1 D, 3 E, 5 G, 34 I, 682 L, 8 Q, 11 R, 37 S, 163 V, 4 W, 1 - | | | 95 E, 470 G, 3 K, 128 R, 1 S, 6 V |
| 814 | 0.475 I | | 0.855 S | | 1 D, 4 F, 511 I, 47 L, 247 N, 124 S, 134 T, 4 V, 2 X, 1 - | | | 1 G, 5 I, 23 K, 72 N, 601 S, 1 X |
| 820 | 0.406 L | | 0.819 I | | 8 C, 154 F, 1 H, 95 I, 436 L, 1 S, 358 V, 1 X, 20 Y, 1 - | | | 576 I, 28 L, 98 V, 1 - |
| 841 | 0.530 I | | 0.731 F | | 8 A, 41 F, 570 I, 369 L, 1 R, 1 S, 1 T, 82 V, 2 - | | | 27 A, 1 C, 514 F, 18 I, 105 L, 35 V, 1 X, 2 - |
| 842 | 0.579 G | | 0.905 C | | 6 A, 417 C, 6 F, 622 G, 4 I, 1 L, 14 V, 1 W, 3 Y, 1 - | | | 636 C, 2 F, 63 G, 1 Y, 1 - |
| 847 | 0.678 H | | 0.993 N | | 1 A, 1 F, 1 G, 729 H, 1 I, 331 N, 1 R, 3 Y, 7 - | | | 3 H, 698 N, 1 S, 1 - |
| 858 | 0.977 R | | 0.888 K | | 1 A, 1 E, 4 G, 1 I, 6 K, 1050 R, 3 S, 1 T, 4 V, 4 - | | | 4 I, 624 K, 1 N, 73 R, 1 X |
| 861 | 0.965 L | | 0.993 Q | | 12 I, 1037 L, 13 Q, 3 V, 10 - | | | 5 L, 698 Q |
